# Tracking the Antibody Immunome in Sporadic Colorectal Cancer by Using Antigen Self-Assembled Protein Arrays

**DOI:** 10.3390/cancers13112718

**Published:** 2021-05-31

**Authors:** María González-González, José María Sayagués, Luis Muñoz-Bellvís, Carlos Eduardo Pedreira, Marcello L. R. de Campos, Jacinto García, José Antonio Alcázar, Patrick F. Braz, Breno L. Galves, Luis Miguel González, Oscar Bengoechea, María del Mar Abad, Juan Jesús Cruz, Lorena Bellido, Emilio Fonseca, Paula Díez, Pablo Juanes-Velasco, Alicia Landeira-Viñuela, Quentin Lecrevisse, Enrique Montalvillo, Rafael Góngora, Oscar Blanco, José Manuel Sánchez-Santos, Joshua LaBaer, Alberto Orfao, Manuel Fuentes

**Affiliations:** 1 Department of Medicine and General Cytometry Service-Nucleus, CIBERONC ISCIII, Cancer Research Center (IBMCC/CSIC/USAL/IBSAL), 37007 Salamanca, Spain; mariagg@usal.es (M.G.-G.); ppmari@usal.es (J.M.S.); pauladg@usal.es (P.D.); pablojuanesvelasco@usal.es (P.J.-V.); alavi29@usal.es (A.L.-V.); quentin@usal.es (Q.L.); emontalvillo@usal.es (E.M.); rgongora@usal.es (R.G.); 2 Proteomics Unit, Cancer Research Center (IBMCC/CSIC/USAL/IBSAL), 37007 Salamanca, Spain; 3 Department of General & Gastrointestinal Surgery, Salamanca University Hospital-IBSAL, 37007 Salamanca, Spain; luismb@usal.es (L.M.-B.); jgarcia@usal.es (J.G.); jaalcazar@usal.es (J.A.A.); lgfcir@gmail.com (L.M.G.); 4 Systems and Computing Department (COPPE-PESC), Universidade Federal do Rio de Janeiro (UFRJ), Rio de Janeiro 21941-914, Brazil; pedreira@cos.ufrj.br; 5 Engineering Graduate Program, Polytechnique School and COPPE, UFRJ/Federal University of Rio de Janeiro, Rio de Janeiro 21941-972, Brazil; campos@smt.ufrj.br (M.L.R.d.C.); patrickfbraz@poli.ufrj.br (P.F.B.); breno.galves@poli.ufrj.br (B.L.G.); 6 Pathology Service, Salamanca University Hospital-IBSAL, 37007 Salamanca, Spain; oscarbengo@usal.es (O.B.); marabad@usal.es (M.d.M.A.); ojblanco@saludcastillayleon.es (O.B.); 7 Medical Oncology Service, Hospital Universitario de Salamanca-IBSAL, 37007 Salamanca, Spain; jjcruz@usal.es (J.J.C.); lbellido@saludcastillayleon.es (L.B.); efonseca@usal.es (E.F.); 8 Statistics Department, University of Salamanca, 37008 Salamanca, Spain; jose@usal.es; 9 Virginia G. Piper Center for Personalized Diagnostics, Biodesign Institute, School of Molecular Sciences, Arizona State University, Tempe, AZ 85281, USA; jlabaer@asu.edu

**Keywords:** metastases, colorectal cancer, auto-antibody profiling, tumor-associated antigen proteins, NAPPArrays, protein antigen array, immunomics

## Abstract

**Simple Summary:**

Immunome in Sporadic Colorectal Cancer as source for biomarkers. Hence, a self-assembled protein array has been designed and developed to perform a serum screening to determined specific immune response against tumor antigens proteins as potential diagnostics biomarker panel.

**Abstract:**

Sporadic Colorectal Cancer (sCRC) is the third leading cause of cancer death in the Western world, and the sCRC patients presenting with synchronic metastasis have the poorest prognosis. Genetic alterations accumulated in sCRC tumor cells translate into mutated proteins and/or abnormal protein expression levels, which contribute to the development of sCRC. Then, the tumor-associated proteins (TAAs) might induce the production of auto-antibodies (aAb) via humoral immune response. Here, Nucleic Acid Programmable Protein Arrays (NAPPArray) are employed to identify aAb in plasma samples from a set of 50 sCRC patients compared to seven healthy donors. Our goal was to establish a systematic workflow based on NAPPArray to define differential aAb profiles between healthy individuals and sCRC patients as well as between non-metastatic (*n* = 38) and metastatic (*n* = 12) sCRC, in order to gain insight into the role of the humoral immune system in controlling the development and progression of sCRC. Our results showed aAb profile based on 141 TAA including TAAs associated with biological cellular processes altered in genesis and progress of sCRC (e.g., FSCN1, VTI2 and RPS28) that discriminated healthy donors vs. sCRC patients. In addition, the potential capacity of discrimination (between non-metastatic vs. metastatic sCRC) of 7 TAAs (USP5, ML4, MARCKSL1, CKMT1B, HMOX2, VTI2, TP53) have been analyzed individually in an independent cohort of sCRC patients, where two of them (VTI2 and TP53) were validated (AUC ~75%). In turn, these findings provided novel insights into the immunome of sCRC, in combination with transcriptomics profiles and protein antigenicity characterizations, wich might lead to the identification of novel sCRC biomarkers that might be of clinical utility for early diagnosis of the tumor. These results explore the immunomic analysis as potent source for biomarkers with diagnostic and prognostic value in CRC. Additional prospective studies in larger series of patients are required to confirm the clinical utility of these novel sCRC immunomic biomarkers.

## 1. Introduction

Sporadic colorectal cancer (sCRC) is the third leading cause of cancer death in the Western world [1]. To a large extent, this is due to the delayed development of symptoms and thus delayed diagnosis at the relatively advanced stages of the disease. In fact, early disease diagnosis leads to significantly higher cure rates due to smaller tumour sizes and less tumour spread. Overall, 15–25% sCRC patients have metastatic disease at diagnosis (e.g., synchronous metastasis) [2], most frequently involving the liver. Currently, complete tumour resection provides the most effective treatment for early-stage sCRC, whereas complementary chemotherapy and/or local radiotherapy is the only effective approaches in a specific subset of the patients, including non-metastatic and a subset of metastatic sCRC patients [3,4].

Current diagnostic approaches for sCRC include invasive approaches (e.g., colonoscopy and classical histopathology), non-wide accessible imaging techniques (e.g., computerized tomography-scans (CT-scan) and magnetic resonance imaging (MRI), and molecular genetic techniques, all of which are not well-suited for population-wide screening for early diagnosis. In contrast, alternative cost-effective approaches based on fecal occult blood testing, measurement of carcinoembryonic antigen (CEA) serum levels, and/or testing for KRAS point mutations in liquid biopsies and/or circulating tumoral DNA (ctDNA) have been adopted or considered for current and future population-based sCRC screening programs. However, their actual benefit is still a controversial topic, mainly due to the relatively high rate of both false positive and negative results [5]. Consequently, the search for an alternative, complementary cost-effective and efficient approaches, suitable for the diagnostic screening of sCRC patients, still remains a challenge.

Previous studies have shown that different tumor types are associated with (humoral) auto-immune [6] responses against tumor-associated antigens (TAA) frequently located in proteins that show altered expression levels, mutations, unique degradation profiles, misfolding or different post-translational modifications (PTM) (i.e., p53 is acetylated, phosphorylated, etc.), as well as ectopic locations inside the cell [7,8]. Even more, recent studies have shown the presence of antibodies against TAA several years before the onset of the symptoms related to the tumor [9,10,11]. In line with these findings, Barderas et al. have found similar humoral response profiles in a murine model of sCRC [11]. In such model, activation of the immune system triggers the first clinical symptoms, which is directly associated with the presence and/or increment of auto-antibody (aAb) serum levels [11]. Since auto-antibodies can be detected at early cancer stages, they can be exploited to increase the percentage of CRC patients diagnosed early. Therefore, the detection of tumor-associated aAb in the serum/plasma represents an attractive and potentially useful strategy for (early) diagnostic screening of sCRC, both in suspected patients and in population-wide screening programs, whenever large panels of aAb markers are simultaneously assessed.

The humoral immune response has been proven to play an important role in CRC. Indeed, TAAs targeted to autoantibodies cancer patients have been identified by protein-microarrays-based proteomic techniques [12,13,14]. Their main advantages include the simultaneous evaluation of aAb against hundreds to thousands of different proteins using a minimally invasive approach in a small volume of sample (e.g., few microliters of plasma) [15,16]. Unfortunately, the set of proteins that can be found in commercial microarray are not tailored for TAAs. Therefore, here, it is proposed to design and develop a customized protein array with the main aim of extending the number of identified TAAs. The development of in situ cell-free protein expression microarrays such as the Nucleic Acid Programmable Protein Arrays (NAPPArray), with improved capture and stability of the proteins linked to the microarray surface, allows analysis of full-length functional human proteins; therefore, the discovery of new biomarkers using high-throughput formats has become feasible [17].

Here, we evaluated the potential utility of the NAPPArray technology for fast and efficient screening of aAb against 2023 (potential) TAAs (as human full-length recombinant proteins) present in the plasma of sCRC patients. Our results pointed out the existence of novel of TAAs (correlated with transcriptomic prognosis), that hold potential value for the diagnostic screening of sCRC and the discrimination between patients with the metastatic and non-metastatic disease.

## 2. Materials and Methods

### 2.1. Patients and Samples

Overall, 57 plasma samples from 7 healthy adults and 50 patients diagnosed with sCRC between January 2008 and December 2010 (17 males and 33 females; median age of 72 years, ranging from 27 to 85 years) were prospectively analyzed. All of patients are näive for any oncotherapy, free of other immunopathologies, no alimentary intolerance and common allergies. All patients were diagnosed and classified according to the WHO criteria at the Departments of Surgery and Pathology of the University Hospital of Salamanca (HUS, Salamanca, Spain). Informed consent was given by each individual prior to entering the study and the study was approved by the local ethics committee of the HUS. In all cases, peripheral blood (PB) samples (10 mL/case) were obtained in K3-EDTA coated tubes, prior to any therapy was administered to the patients. Immediately after collection, PB samples were centrifuged, and the plasma was stored at −80 °C until analyzed.

Around one-fourth (12/50; 24%) of the patients presented synchronic metastases, while the remaining 38 cases (76%) had non-metastatic tumors. The most relevant clinical and laboratory patient data recorded at diagnosis are summarized in Table 1. At the time of closing this study, the median follow-up was 45 months (range: 0 to 101 months).

### 2.2. Nucleic Acid Programmable Protein Array (NAPPArray) Performance and Serum Screening Conditions

The NAPPArray designed here contained 2023 unique cDNAs encoding for an identical number of (antigenic) human proteins (full-length recombinant proteins verified by sequencing) related to cancer and selected from the Medgene (http://medgene.med.harvard.edu/MEDGENE/, accessed on 18 April 2014) and Biogene (http://biogene.med.harvard.edu/BIOGENE/databases, accessed on 18 April 2014) (Table S1) as they include information about potential TAAs. In addition, multiple negative and positive controls have been included such as GST, human IgG, EBNA, empty cDNA, among others (Table S1) to decipher any patients subgroups. For quality assurance, the constructed NAPPArray also contained positive and negative controls. These arrays were designed and prepared following standard operating procedures (SOPs) at the Biodesign Institute of Arizona (Tempe, AZ, USA) according to the techniques described by Ramachandran et al. (17) and adapted by Manzano-Roman et al. [18] and Henjes et al. [12] (see Supplementary Materials and Section 2).

Quality control (QC) evaluations of the NAPPArrays were performed to ensure high reproducibility and robustness of the proteins displayed on the array via in vitro transcription/translation (IVTT) [9,10,18]. Therefore, the overall cDNA linked to each well in the array was assessed by Picogreen staining (Invitrogen, Paisley, UK) following the instructions of the manufacturer; in turn, in situ protein expression levels were evaluated by the TNT T7 Coupled Reticulocyte Lysate cell-free expression system (Promega, Madison, WI, USA), as described elsewhere [9,10,18] (Supplementary Materials and Section 2).

For the detection of aAb, NAPPArrays were incubated overnight at 4 °C with the 1/600 (*v*/*v*) diluted plasma samples with continuous gentle mixing [9,12,14]. To reveal the aAb-protein conjugation, the NAPPArrays were incubated with anti-human-IgG antibodies coupled to horseradish peroxidase protein (HRP), followed by the amplification of the signal using the tyramide signal amplification (TSA) reagent (PerkinElmer, Shelton, CT, USA), following the manufacturer’s instructions (Figure S1). Finally, these arrays were scanned for image visualization using a Genepix 400B (Axon Instruments, Sunnyvale, CA, USA) scanner.

### 2.3. Identification of Differential aAb Serum Profiles Across the Analyzed Samples

Scanned NAPPArray images were analyzed using the Genepix Pro 6.0 (Axon Instruments, Sunnyvale, CA, USA) image analysis software. During analysis, data intensity at 532 nm was recorded. Raw data were normalized as follows: (1) firstly, a background correction was used to eliminate unspecific fluorescence for each spot in the array, background levels were estimated as the first quartile of the values obtained for the empty spots (nonspot) and then (2) the value for each spot was normalized by calculating the ratio between each corrected value (from step 1) and the median of all spots that contained the empty pANT7_cGST vector.

Then, the normalized data sets were employed to decipher h for the potentially different aAb plasma profiles, considering the following sample groups: healthy donors vs. sCRC, and non-metastatic sCRC vs. metastatic sCRC. Firstly, we identified the aAbs presented in the plasma samples analyzed, considering them as positive aAbs if their mean normalized value was higher than (mean value + 3SD) empty pANT7_cGST vector normalized value. After that, these positive aAbs were evaluated by using the non-parametric Mann-Whitney U (MW) test (statistical significance set at *p* < 0.05), via the MultiExperiment Viewer 4.5.0 (MeV) (software available at www.tm4.org, accessed on 13 November 2009), in order to identify a set of aAbs that might discriminate between sCRC patients vs. healthy donors. In addition, the immunome of these sCRC patients was defined based on the presence of aAbs accomplishing the following four criteria: (i) to show distinct aAb profiles (*p* < 0.05) in the univariate analysis (Mann–Whitney U test), (ii) no presence in the healthy-donor group, (iii) to be detected in ≥2 sCRC individual samples, and (iv) the changes observed in the normalized fluorescence intensity data of aAb for the sCRC patients (group 2) included in the study were higher than the corresponded normalized signal in the healthy donors (group 1), the threshold was established in fold change (FC) values >1, calculated FC for each TAAs and case as follows:

(1) $$\mathrm{FC}=\log2\left[\left(\frac{\mathrm{group}\;2}{\mathrm{median}\;\mathrm{group}\;1}\right)+1\right]$$

Finally, in order to identify the differential aAb profile—that might distinguish between sCRC patient sub-groups at diagnosis (non-metastatic vs. metastatic sCRC)—a non-parametric Mann-Whitney U (MW) test (*p* < 0.05) was applied to this set of aAbs previously defined and included as part of the sCRC immunome. Additionally, the changes observed in the normalized fluorescence intensity data of aAbs for the non-metastatic sCRC patients (group 1) vs. metastatic sCRC (group 2) were calculated according to the FC described above.

### 2.4. Functional In Silico Analysis

A protein network analysis was performed by using STRING tool (version 10.5) in order to identify the associations of all the TAAs identified and that could be included in this sCRC immunome. Moreover, the TAA candidates were compared in the Auto-antigen Atlas data Base AAgAtlas (version 1.0) to check if any of these TAAs have been previously reported in other solid tumors or other pathologies [19].

The role in biological processes and subcellular localization of this set of TAAs (defining the sCRC immunome) were further screened by a functional enrichment analysis (FEA) performed using the DAVID functional annotation bioinformatics tool for microarrays analysis (Bioinformatics Resources, version 6.7) and the Gene Ontology (GO)—annotation spaces for biological processes (GOTERM_BP), cellular components (GOTERM_CC) and molecular functions (GOTERM_MF)—databases; the stringency was set at medium to generate the final report. Those TAAs presenting a relevant role (e.g., highly significant in the gene ontology analysis) were further reviewed in the existing databases to depict their involvement in the sCRC. In order to generate and visualize the networks between the GO terms resulted of FEA, the Cytoscape software (version 3.3.0) were employed.

### 2.5. Feature Selection Algorithms and Linear Models

A regularized version of the logistic regression, called LASSO [20], was used to model the relation between proteins and patients classification in groups (e.g., metastatic versus non-metastatic). The key idea behind the LASSO family methods is to include in the cost function, apart from the regression term, a penalization expressed as a function of the values of the parameters. Intuitively, by means of this penalization, one charges a price for the ‘amount of used’ parameters (associated with the proteins). Accordingly, the parameters relative importance is minimized in the cost function. The algorithms search for optimal coefficients, each associated with one protein. The higher the value of a coefficient, the more important the associated protein is in explaining the outcome.

In this context, each patient is represented as a point in a linear vector space, aAbs are the variables in the linear regression model, and we seek a hyperplane to divide the two groups, i.e., metastatic and non-metastatic patients. The LASSO algorithm was the chosen constrained least squares algorithm used in order to determine which variables, or aAbs, should be considered to contain most of the information necessary to separate the samples.

For each patient, the data set provides a categorical variable mapped as +1 for metastatic and −1 for non-metastatic clinical condition. We calculated the error between the categorical variable and the outcome of the linear regression model, which is a function of the aAb concentrations. The chosen algorithms are known to provide a sparse set of coefficients of the separating hyperplane. Due to the small number of samples available, we used leave-one-out cross-validation, where in each iteration a single subject with metastasis was set aside for validation, and the training was carried out with the remaining eleven metastatic subjects, and randomly selected eleven non-metastatic patients. Validation used the training parameters obtained during training but tested it on the left-out patients not used for training, i.e., the patient with metastasis left out and the 28 non-metastatic patients. This procedure was repeated for all metastatic patients; therefore, performance metrics were calculated for twelve rounds of validation.

### 2.6. Data Visualization

Clustering analysis is performed by Ward Method with 2 within the pathology (metastatic vs. non-metastatic) vs. healthy donors; all pathologies (all of them in one single group) vs. healthy donors (as controls). In all the data analysis, several biostatistical approaches (camberra distance, silhouette distance, k-mean cluster, canonical biplots, logistic regression, random forest based multiclass, receiver operating characteristics curve (ROC)) were studied with R-studio interface.

### 2.7. Validation of a Featured Panel of TAAs

To confirm the diagnostic value of our results a total of 6 (USP5, MARCKSL1, CKMT1B, HMOX2, VIT2, TP53) out of 141 TAAs included in the sCRC immunome as well as a positive control (EBNA) were validated by ELISA assays performed according to the techniques described by Henjes et al. [12]. A total of 57 plasma samples from 7 healthy adults (negative colonoscopy) and 50 patients diagnosed with sCRC included in this validation were provided by Spanish National DNA Bank Carlos III (BNADN, University of Salamanca, Salamanca, Spain). Prior to entering the study, patients gave their written informed consent to participate according to the Declaration of Helsinki; the study protocol was approved by the External Ethical Committee of the Spanish National DNA Bank Carlos III.

## 3. Results

### 3.1. Performance of the NAPPArray for sCRC-Associated Plasma aAb Screening

**Figure 1:**
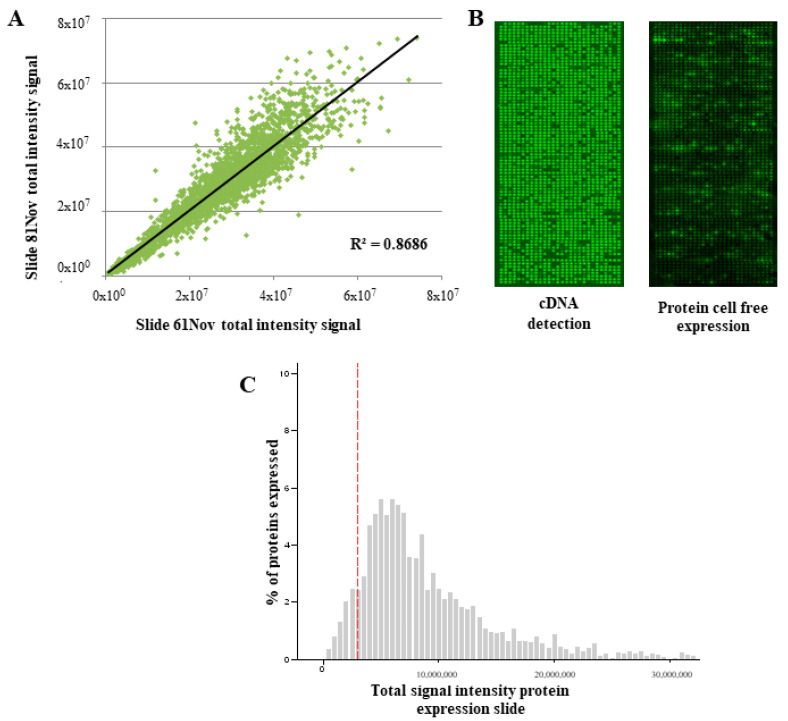
Overall performance of customized NAPPArray. (**A**) displays scatter plot of raw data (total signal intensity) correlation of two different arrays of cDNA stained assessment that illustrate the reproducibility of assays. (**B**) corresponds to high-density NAPPArray displaying 2164 antigenic tumoral proteins included in this study. cDNA stained at described in Section 2; IVTT protein cell-free expression was detected by the α-GST monoclonal antibody. (**C**) shows the relationship between cDNA and protein expressed in NAPPArray platform represented by the histogram of expression total signal intensity and the cell-free protein expression frequency, the red line corresponds to the median of total intensity values of negative controls (nonspot) + 2SD.

Prior to plasma screening, QC assays of NAPPArray were performed in order to assess the reproducibility and robustness of the NAPPAarray platform in the screening of aAb presented in sCRC plasma samples. Detection of printed cDNAs (encoding full-length recombinant C-terminus GST-tagged proteins) containing TAAs (as it is described in the material and methods section) was accomplished to verify the presence of all cDNAs deposited on the NAPPArray. Spots containing cDNA displayed significantly higher-intensity values vs. negative control spots (e.g., those without cDNA) which showed no signal. Of note, a high correlation (R2 > 0.85) was observed for the stained cDNA on the NAPPArrays assayed, further confirming the reproducibility and robustness of the printed microarrays (Figure 1A).

After IVTT protein expression, the presence of in situ expressed carboxy-GST tagged recombinant proteins was confirmed by the tag detection with an anti-GST tag monoclonal mouse antibody (Figure 1B). Thus, in situ expressed (human) proteins were detected in >92% of the spots containing cDNA (encoding human recombinant proteins); in contrast, the negative control spots (Figure 1C) showed no protein expression as expected. In summary, these set of NAPPArrays, containing >2000 TAAs, showed a high reproducibility both within individual arrays and among different arrays with low variations (CV < 5%) (e.g., “day-to-day” or “zone”).

### 3.2. Identification of aAb Profiles in Healthy Donors vs. sCRC Plasma Samples

**Figure 2:**
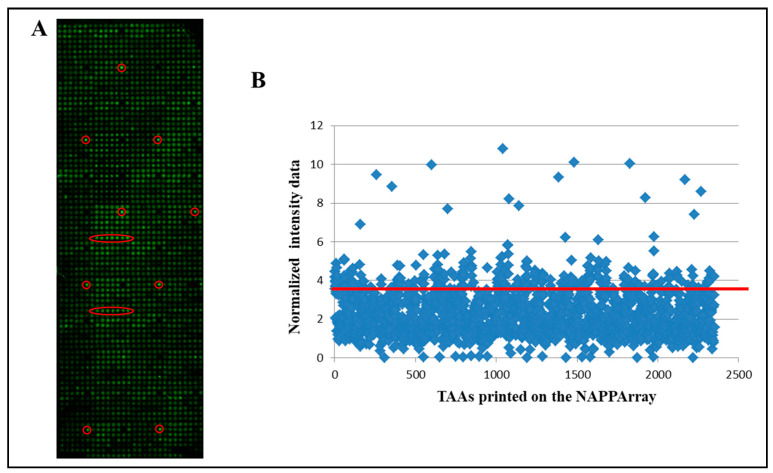
Detection of aAbs on NAPPAarray. (**A**) Illustrates a representative slide image showing the antibody reactivity against TAAs included on the NAPPArray. aAbs against tumor antigens are highlighted in red. (**B**) Corresponds to scatter plot of the total intensity of aAbs against all the TAA contained in the NAPPArray. Red line corresponds to mean of the normalized values of empty pANT7_cGST vector spots + 3SD and the positive aAbs defined taking into account the normalization values (>mean of the normalized values of empty pANT7_cGST vector spots + 3SD).

A cohort of 57 plasma samples from 7 healthy donors (with negative colonoscopy) and 50 sCRC patients were screened for aAb directed against the IVTT expressed proteins displayed on the NAPPArray. Overall, the detection of plasma aAbs-against the displayed TAAs- was feasible by the workflow with NAPPArray technology depicted in Figure 2. The aAbs were defined considering the normalized signal values (>mean of the normalized values of empty pANT7_cGST vector spots + 3SD).

**Figure 3:**
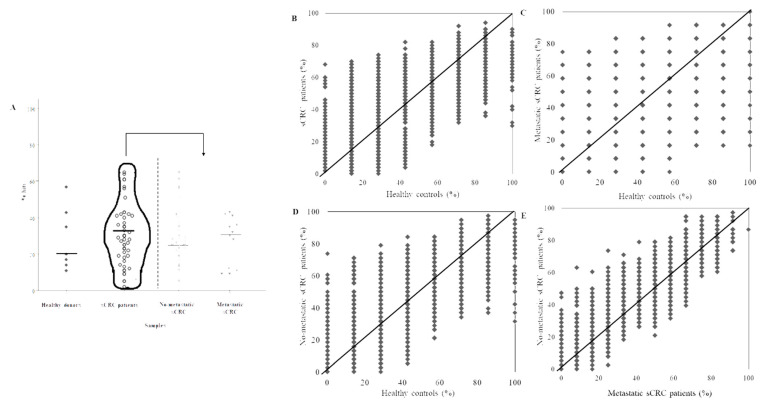
Distribution of positive aAbs (*n* = 1928). (**A**): Samples’ immunoreactivity represented by the percentage of all positive hits detected in the NAPPArray for each group of samples. (**B**–**E**): The figures show the pair-wise group comparisons of the percentage of reactivities samples per antigen, healthy controls vs. sCRC patients (**B**), healthy controls vs. metastatic sCRC patients (**C**), healthy controls vs. non-metastatic sCRC patients (**D**), metastatic sCRC vs. no-metastatic sCRC (**E**).

Thus, a total of 1928 out of the 2023 TAAs displayed in the array showed immunoreactivity with plasma aAb in at least one analyzed case in this study. The global number of aAbs identified above the threshold ranged from 48 (2%) to 1288 (64%) aAbs (Table 2). In addition, a great variability of unique TAAs was detected for each patient group and healthy donors. Accordingly, the number of immunoreactivity to TAAs hits per patient was slightly higher in sCRC patients (median = 29%) vs. healthy donors (median = 20%) (Figure 3A). Furthermore, metastatic sCRC was the group with the largest number of immunoreactivity TAAs hits (median = 31%) vs. non-metastatic sCRC (median = 27%) (Figure 3A). Considering the distribution of those TAAs by sCRC patients/healthy donor groups, our results showed similar immunoreactivity ratios between healthy controls vs. sCRC patients (Figure 3B), healthy controls vs. metastatic sCRC patients (Figure 3C), healthy controls vs. non-metastatic sCRC patients (Figure 3D) as well as non-metastatic vs. metastatic sCRC patients (Figure 3E).

### 3.3. Determination of Differential aAb Profiles

**Figure 4:**
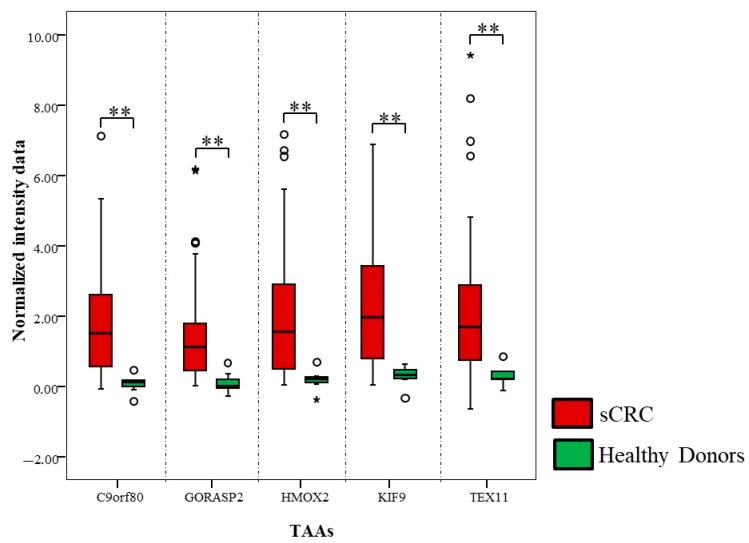
Normalized intensity data of aAbs against TAAs with statistical significance (*p* < 0.001) from healthy controls (*n* = 7) and sCRC patients (*n* = 50) are represented by double asterisk (**). Notched-boxes extend from the 25th to 75th percentile values; the lines in the middle and vertical lines correspond to the median values and the 10th and 90th percentiles, respectively. Outlier cases, identified as cases between 1.5–3 and/or >3 fold the interquartile range, are represented by circles and star, respectively.

Bearing in mind the aAb heterogeneity within analyzed groups of samples, the Mann-Whitney test was performed (as conventional biostatistics analysis) to distinguish aAb profiles that might discriminate healthy controls vs. sCRC patients, as well as in non-metastatic vs. metastatic sCRC patients. In this sense, our results showed that 141 aAbs accomplished the four criteria described in the material and methods section resulting in the sCRC immunome (Table S2). A total of 67 out of these 141 aAbs (Table 3) had statistical significances with a value of lower than 0.01, where aAbs against corresponding the highest statistic values (*p* < 0.01), being aAbs against C9orf80, GORASP2, HMOX2, KIF9 and TEX11 (Figure 4) the most significant (*p* < 0.001). The aAbs constituting the sCRC immunome presented mean FC values > 1.5, with more than 78% of the sCRC patients showing FC values > 1 (Table S3).

**Figure 5:**
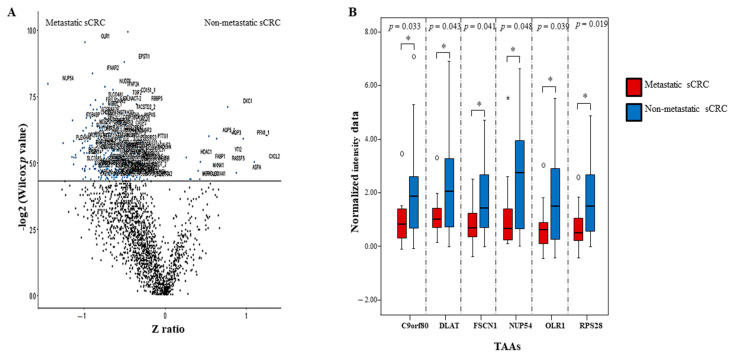
Identification differential aAbs profile between metastatic vs. non-metastatic sCRC. (**A**) Shows volcano plot of all TAAs presented (*n* = 141) in the sCRC analyzed samples. (**B**) Represents normalized intensity data of aAbs against TAAs with statistical significance (*p* < 0.05) from metastatic sCRC (*n* = 12) and no-metastatic sCRC patients (*n* = 38). Notched-boxes extend from the 25th to 75th percentile values; the lines in the middle and vertical lines correspond to median values and the 10th and 90th percentiles, respectively. Outlier cases, identified as cases between 1.5–3 and/or > 3 times the interquartile range, are represented by circles and stars, respectively.

To determine the aAbs that might discriminate between non-metastatic and metastatic sCRC patients, we analysed the distribution of the sCRC immunome (141 aAbs) by non-metastatic and metastatic sCRC patients identifying similar immunoreactivity ratios for both sCRC groups (metastasic and non-metastatic) (Figure 5A). Bearing in mind these results, those 141 aAbs were included in the Mann-Whitney test in order to identify the differential aAb profile of non-metastatic and metastatic sCRC. A small set of aAbs (such as NUP54, Corf80, FSCN1, OLR1, DLAT, RPS28, among others) presented statistical significance (*p* < 0.05) and might potentially distinguish non-metastatic sCRC vs. metastatic sCRC (Figure 5B). Of note, these aAbs presented FC values lower than 1 and nearly half of the metastatic sCRC samples showed FC values > 1 (Table S4).

### 3.4. Functional In Silico Analysis of the Differential aAb Profiles Against TAAs in sCRC Patients

According to the results described above, the content of sCRC immunome might be defined by 141 TAAs (screened with NAPPAarrays); thus, any functional relation between them could suggest novel therapeutic targets. In fact, 56% of TAAs (79 out of 141) have been related between them through a different kind of interaction as reflected from FEA analysis (Table 4).

The comparisons between 141 TAAs that generated sCRC immunome and the auto-antigens included in the AAgAtlas database revealed that a small groups of TAAs (PIXT3, YBX1, DRD2, SCARB1 SERPING1, TPTE, DLAT, and SPP1) were found to be overlapping between the lists, furthermore, only four, SERPING1, TPTE, DLAT, and SPP1, were associated with oncologic disorders [21,22,23].

The evaluation of functional in silico analysis showed that top five subcellular localization of the 141 aAbs included in the sCRC immunome (Table S2) and revealed that these TAA proteins were mainly located in three distinct cellular compartments (Table 4): extracellular compartment, cytoplasm, and membrane; most frequently presented in the extracellular location (46 TAAs) and cytoplasm (42 TAAs) compared to membrane (18 TAAs). The results of functional enrichment analysis for the biological functions of these TAA proteins revealed that 7% (10 out of 141) of TAAs (Table 4) were mainly related to the different steps of translational process, where most of them belong to the ribosomal protein family (Table 4). In this line, the most relevant molecular function of these TAAs were protein binding, that involved 61% (86 out of 141) of unique TAAs, vs. cell–cell adhesion 6% (8 out of 141) of TAAs, both clearly involved in the tumoral microenvironment. Taking into consideration the analysis of protein function and subcellular localization, it is appropriate to highlight that 55 out of 109 unique TAAs have been previously reported as proteins associated with sCRC [24,25,26,27].

Concerning the differential TAAs proteins which can discriminate between non-metastatic and metastatic sCRC (Table 4), the subcellular localization is quite heterogeneous, where most of them are located at the nucleus (NUP54 and C9orf80) and cytosol (FSCN1 and RPS28). Regarding biological process, none of the TAA proteins were involved in the same or related procedure (Table 4). Conversely, four (C9orf80, FSCN1, OLR1, and RPS28) out of six TAA proteins were related to protein binding (Table 4).

### 3.5. External Evaluation of a Potential Panel of aAbs as sCRC Useful Biomarkers

**Figure 6:**
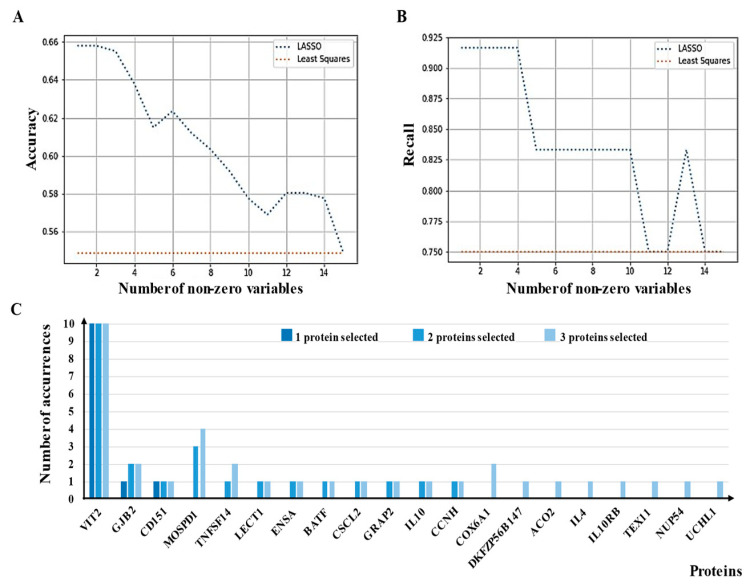
In silico prediction of a potential panel of TAAs as biomarkers for sCRC. (**A**)—Accuracy evolution of the lasso algorithm versus number of proteins selected. (**B**)—Recall evolution of the lasso algorithm versus number of proteins. (**C**)—Histogram of proteins chosen by the lasso algorithm.

As an alternative attempt to quantify the importance of individual protein markers for discriminating between metastatic and non-metastatic sCRC patients, we employed linear mapping techniques mentioned with feature selection constraints [19,20,28]. Figure 6A,B shows the progression of accuracy and recall, respectively, averaged for the validation process, as a function of the increasing number of variables in the active set, i.e., the set of aAbs the algorithms deemed relevant and given non-zero weights. Although accuracy provides information about the overall performance of the classification, recall counts only the performance for metastatic patients. A large recall indicates a small miss probability. Although the performance of the algorithm was important to establish reliability and applicability for the given data set, one goal of the study was to establish the usefulness of variables, or protein markers. In this aspect, if a voting rule should guide our measure of biomarker relevance, clearly VTI2 (Vesicle transport through interaction with t-SNAREs homolog 1B)-as TAAs- stands out (Figure 6C). This result could not be obtained from standard statistical correlation measures, such as *p*-value, and can be a useful asset for scientists interested in a more comprehensive evaluation of diagnostic and prognostic biomarkers for CRC or biomarkers to discriminate +/− metastatic CRC.

Proper tuning of the hyper-parameters of the algorithms yields a different number of variables with non-zero weights. Figure 6C shows TAAs singled out by the algorithm when the parameters were tuned for selecting three, two, or only one protein. The number of correct and incorrect classifications obtained by the algorithm selected by one protein (VTI2) is summarized in Table S5.

**Figure 7:**
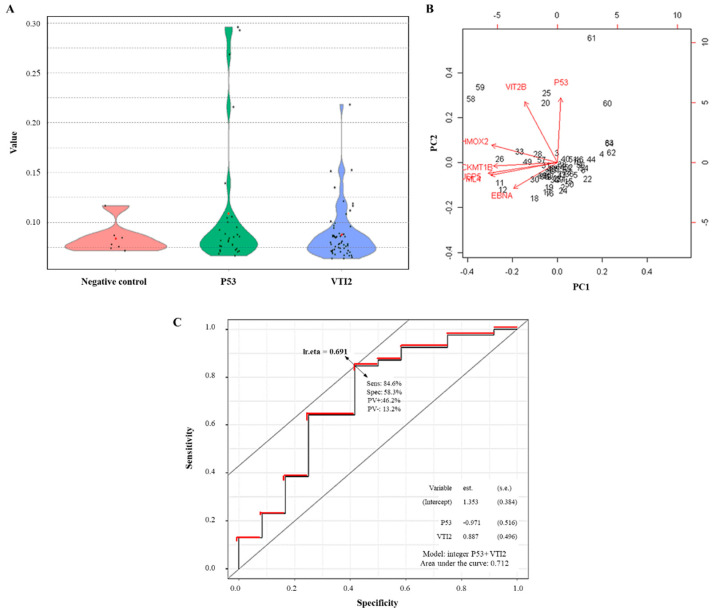
Validation of potential TAAs candidates in metastatic vs. non-metastatic sCRC. (**A**) Results obtained by NAPPA-ELISA in the validation cohort of sCRC patients (described in Section 2). (**B**) Canonical biplot of the 8 TAAs potential biomarker panels. (**C**) ROC analysis of dataset corresponding to validation cohort of sCRC patients (described in Section 2).

The NAPPA-ELISA assay for VTI2 with an independent cohort of sCRC confirm these findings and applicability of these lasso algorithms in TAAs discovery (Figure 7A,B). Additionally, as p53 has been previously reported as TAAs in these tumors, it was also included in order to test the increased discrimination performance of VTI2 and p53 as a biomarker panel for metastatic vs. non-metastatic sCRC. P53 and VTI2 proteins are differently reported in crapome databases (Table S6), which reports a low rate of false negative detections in VTI2; then, these previous observations help to add more value to these findings. The cooperative discrimination capacity of this TAAs proteins were explored by canonical biplots and ROC analysis (Figure 7B,C); where it is differentially observed that both proteins are displayed in the similar profile as TAAs with a promising AUC value > 70%.

## 4. Discussion

sCRC is one of the most prevalent tumors in the Western world with relatively high mortality rates, mainly due to delayed diagnosis of the disease. Current clinical guidelines include the detection of fecal occult blood (FOB), using the guaiac-based test or immunochemical methods as the most suitable early diagnostic method for sCRC [5,29,30]. However, several studies have proved the ineffectiveness of FOB due to its high variability and lack of sensitivity and specificity as other diverse conditions could also lead to fecal blood [30,31].

In order to develop a more efficient diagnostic screening approach for early diagnosis of sCRC, multiple attempts have focused on the identification of potential biomarkers in liquid biopsies and/or ctDNA, particularly in PB, serum and plasma, that could be easily incorporated in the diagnostic phase [31]. Thus, identification and quantitation of PB-circulating tumor cells and/or ctDNA have been proposed as a promising tool to monitor sCRC patients and to evaluate their response to therapy [32,33]. Determination of free circulating tumoral DNA (ctDNA) present in the serum of sCRC represents another alternative promising strategy, particularly when based on the identification of KRAS and BRAF mutations, which could be complementary with immunome in order to better understand the pathology and to increase the accuracy and precision of the diagnosis and prognosis [34,35].

Profiling the sCRC immunome, the aAb profile against sCRC TAA present in serum or plasma, has also emerged as a powerful method to discover potentially useful biomarkers for the early diagnosis of multiple human solid tumors; mainly due to: (i) the production of such aAb is a consequence of the activation of tumor-specific humoral immune responses at the earliest stages of the disease (even prior to its first symptoms and signs), (ii) the stability of the immunoglobulins in plasma and, (iii) the relatively simplicity of the immuno-assays commonly used to assess their specificity in clinical laboratories [36,37,38]. In this regard, NAPPArrays have become a powerful tool in the identification of differential TAA profiles by the detection of aAb in different tumoral disorders and several pathologies that could produce functional proteins in the moment of the assays [10,12,14].

Here, a customized NAPPArray platform, containing 2023 potential TAA proteins, was used to define the immunome of 50 sCRC patients (12 metastatic sCRC and 38 non-metastatic sCRC) and 7 healthy donors (negative colonoscopy)). Highly variable numbers of aAb were detected per case, also including heterogeneous TAA proteins associated to different aAb profiles, as might be expected due to the high genetic (inter and intra-tumoral) heterogeneity of sCRC tumors [10,37,38,39].

In this study, the results showed that 7% (141 out of 2023) of unique TAAs displayed in the NAPPArray allowed discrimination between healthy donors and sCRC patients. The functional in silico analysis of the 141 TAAs that generate the sCRC immunome, showed that the extracellular compartment was the most enriched localization. This might be expected because tumor cells try to modify the cellular microenvironment secreting different molecules with the aim of facilitating the growth and the invasion [39,40]; however, the secreted proteins might become auto-antigens and activate the immune system with the production of specific aAbs [7,8,38,40]. In a similar study, Barderas et al. found TOLLIP, MARCKSL1, and FSCN1 in the secretome of sCRC [41] molecules implicated in carcinogenesis and progression of cancer [42,43,44]. FSCN1, especially, has been reported as a potential prognostic biomarker in sCRC patients [45,46,47,48], supporting our results.

It should be noted that intracellular signaling related to translational–transcriptional processes were detected as the principal biological processes related to the TAAs of the sCRC immunome, most frequently involving ribosomal proteins. These proteins are essential for protein biosynthesis as well as RNA splicing and modification, cell growth, and proliferation, regulation of apoptosis and development of tumor cells [49,50,51] via signaling pathways usually altered in sCRC like Id-1/PI3K/Akt/NF-κB or p53 signaling pathway [49,51,52]. In accordance with our results, Coronell et al. have recently defined the immunome of colon cancer employing protein microarrays that include aAbs against ribosomal proteins (RPS9 and RPL18) [53]. Additionally, Garranzo-Asensio et al. recently reported a set of 31 proteins with altered expression at mRNA level in a serum characterization by protein microarrays [54].

Besides the gene expression function, a total of 86 out of 141 immunome TAAs show protein binding functions involved in the stabilization of the cellular structures, where 22% (19 out of 86) of them are located at plasma membrane [54,55]. Among these proteins, alterations in the FSCN1 expression levels have been associated with a more aggressive phenotype and invasion in sCRC mainly due to its involvement in the stability of actin-based structures that aid in cell motility [56,57,58]. ANXA9 and ANXA13 belong to a protein binding family located in the plasma membrane and are involved in different biological processes such as vesicle transport, calcium signaling, cell growth, and apoptosis [59]. High expression levels of these proteins have been associated with cell invasion and metastasis in sCRC as well as adverse prognosis [24,25,60]. Alterations in the expression levels of these binding proteins might cause instability of the cellular structures and become auto-antigens in sCRC that have been detected in several studies [41,53,61]; in this line, Cha et al. have identified 163 pairs of antibody peptides and possible antigenic peptides that belonged to aberrant proteins [62].

In order to determine the importance of 141 TAA as antigens in other tumors, the updated version of open AAgAtlas database was consulted to find four TAAs to have been previously reported as auto-antigens related to solid tumors. Among them, only two, SERPING1 and SPP1, were associated with gastrointestinal cancers. AAgAtlas stated the relation of aAb against of SERPING1 with the response to treatment of auto-immune disease angioedema with gastrointestinal affection [61].

Regarding the aAb profile that might discriminate between non-metastatic and metastatic sCRC patients, NUP54, C9orf80, FSCN1, OLR1, DLAT, RPS28, VTI2, and p53 proteins were detected. As an oncogenic protein with high number of point mutations and altered expression level, p53 has been previously reported and well-documented as TAA in sCRC [9,62]; even with different PTMs have been previously reported (such as acetylated, phosphorylated, etc.) which is related to the modifications of HLA presentation and TCR recognition of these p53 as TAA [9]. FSCN1 has been associated with metastatic processes in sCRC as described above [47,58]; as well as OLR1, membrane receptor for oxidized low-density lipoprotein, that is considered as a risk factor to sCRC [63] since the interaction of these molecules increases the formation of reactive oxygen species, via NF-kB signaling pathway, strongly contributing to oxidative DNA damage, carcinogenesis development [63,64,65], and progression of sCRC disease [66]. Furthermore, Murdocca et al. have postulated this lipoprotein receptor as a potential drug target for sCRC [67]. However, OLR1 has not been yet reported in the latest version of Pathology Atlas from the Human Proteome Atlas.

On the other hand, NUP54, c90rf80, RSP28, DLAT have been previously related with sCRC carcinogenesis and metastatic processes; however, their role as TAAs in sCRC have not been reported before. Since, TAAs are usually highly dysregulated at protein level in CRC tissue, we have explored in well-established bioinformatics databases if those proteins might show any dysregulation in tissue and whether they show any diagnostic or prognostic ability at transcriptomic and genomic level (i.e., mRNA, copy number variation, SNPs, etc.). NUP54 is a component of the nuclear pore complex required for the trafficking across the nuclear membrane, being quite relevant to protein transportation, mRNA translocation and transportation. NUP54 expression level has been observed to be dysregulated in sCRC as reported by OncoMX, TCGA in cBioPortal, CPTAC and UALCAN data bases [20], which is also correlated with the infiltration of immune cells. Similarly, C9orf80 (INIP) is also a nuclear protein related to protein synthesis and transportation, etc., as interacting partner with NABP and NST3 proteins. It is also up-regulated in CRC in comparison with normal tissue surrounding the tumor cells [55]. Another protein involved in RNA binding and protein synthesis as part of the ribosome structure is RPS28; which is mainly located at endoplasmic reticulum and cytoplasm. Furthermore, its expression is also dysregulated in sCRC tumor cells as described in OncoMX, TCGA in cBioPortal, UALCAN and CPTAC [19,28].

In contrast with these proteins mentioned above that were not previously described as the target of aAbs, DLAT (E2 component of pyruvate dehydrogenase complex) has been previously reported as an aAb in patients with liver disease, in particular in primary biliary cirrhosis which manifest with inflammatory obliteration of intra-hepatic bile duct, leading to liver cell damage and cirrhosis [28]. Additionally, DLAT was found to be a CRC prognosis marker in the Pathology Atlas from the Human Proteome Atlas (Figure S3). In the same manner, NUP54, C9orf80 and VTI2 transcripts are listed also as CRC prognosis markers in the Pathology Atlas from the Human Proteome Atlas (Figure S3). Moreover, OLR1 and RPS28 are also described as CRC prognosis marker but with a different trend (Figure S3) because low expression level is reported in the Pathology Atlas in comparison with DLAT, NUP54, C9orf80 and VTI2 (which displayed a high-expression level) [28].

This panel of eight detected aAbs targeting the TAAs in sCRC, has been identified and appear in <10% of agarose-based immunoprecipitation experiments registered in CRAPome (Table S6). Bearing this meta-analysis, CRAPome might be quite useful to remove false positive aAbs targeting the corresponding TAAs. All of the eight proteins have been detected ranging from the well-documented p53 as TAAs (52/411 experiments) and RPS28 (182/411 experiments) to VTI2 with very low detection ratio (1/411 experiments).

Therefore, collectively these data show that the identified proteins that are potential targets of aAbs in sCRC are dysregulated at a genetic and/or protein level in CRC.

Bearing in mind the low number of MS assays detecting VTI2 (as described in www.crapome.org, accessed on 1 March 2020), and in order to confirm VTI2 as TAAs in sCRC, an external validation has been performed to evaluate aAbs against VTI2 in an independent cohort of sCRC; compared with p53 as well-known TAAs in sCRC. As depicted in Figure 7, VTI2 as aAbs could discriminate between healthy vs. CRC and also between sCRC patients (metastatic vs. non-metastatic) as p53 aAbs. Furthermore, their dysregulation seems to play an important role in the alteration of the molecular pathways and cellular functions involved in the pathology. P53 is well-known and well-characterized as tumor-antigens and the aAbs against this protein have been identified in CRC and other solid tumors [62]. Regarding VTI2, it is a protein reported as highly expressed on digestive mucosa and intestinal epithelium; despite of this, it is the first time, so far, that it is reported to be a target of aAbs in sCRC, which could open the potential as a biomarker candidate. The combination of both aAbs, VTI2 and p53, have reported an area under the curve above 70%, which is quite promising for further studies and open the potential to explore the aAbs as a suitable source of biomarkers.

## 5. Conclusions

In summary, herein we have described the application of the NAPPArray technology to identify the immunome of sCRC patients as well as to discover potential aAbs which might be considered as early diagnostic biomarkers for the sCRC disease, including both metastatic and non-metastatic conditions; or compatible with diagnostic and/or prognostic biomarkers in liquid biopsy or ctDNA. In this line, our results identified sCRC immunome that included 141 aAbs against TAAs as well as aAbs that might distinguish non-metastatic vs. metastatic sCRC patients. A panel of TAAs (p53, VTI2, NUP54, RPS28, DLAT, C9orf80) was identified that could be a potential biomarker candidates for early diagnostics and prognostic evaluation in sCRC. These promising results, in particular p53 and VTI2 as TAAs, belong to the discovery phase; therefore, further studies are still required to confirm, validate and verify the potential use of this panel as early diagnostic biomarker in sCRC.

Collectively, these results highlight the usefulness of the presented approach to identify TAAs with significant diagnostic ability. In addition, these results suggest that the here defined CRC TAAs might be included in a sCRC blood-based biomarker panel to get a clinically useful blood-based diagnostic signature for sCRC detection.

## Figures and Tables

**Table 1:** Clinical and biological characteristics of non-metastatic (*n* = 38) vs. metastatic (*n* = 12) sporadic colorectal cancer (sCRC) patients.

Clinical Characteristics	Metastatic sCRC (*n* = 12)	Non-Metastatic sCRC (*n* = 38)	*p*-Value	Total Cases (*n* = 50)
Age (years)	63 (28–76)	71 (27–85)	NS	72 (28–85)
Gender				
Female	6 (50%)	27 (72%)	NS	33 (67%)
Male	6 (50%)	11 (28%)		17 (33%)
Tumor Localization				
Rectum	6 (50%)	23 (61%)	NS	29 (59%)
Left colon	2 (33%)	1 (3%)		3 (6%)
Right colon	4 (17%)	14 (36%)		18 (35%)
Histological grade				
Well-differentiated	3 (53%)	1 (3%)	*p* = 0.03	4 (8%)
Moderate- differentiated	2 (28%)	10 (26%)		12 (40%)
Poorly- differentiated	2 (29%)	12 (31%)		14 (47%)
Lymph node status (TNM)				
pN0	4 (33%)	18 (46%)	NS	22 (43%)
pN1	6 (50%)	16 (44%)		22 (45%)
pN2	2 (17%)	4 (10%)		6 (12%)
Tumor Size (cm)	5 (2–11)	3,5 (1,7–7)	NS	4.7 (1.7–11)
Serum CEA (ng/mL) *	11.8 (1.2–344)	4.9 (0.9–90)	NS	5.5 (0.9–344)
N. of Deaths	9 (75%)	9 (23%)	*p* = 0.001	18 (35%)
Median OS (months) *	14 (0–64)	22 (0–40)	*p* = 0.001	Not reached

Results expressed as number of cases (percentage) or * median (range); NS: statistically not significant (*p* > 0.05); F: female; M: male; CEA: carcinoembryonic antigen; OS: overall survival.

**Table 2:** aAb against unique TAA proteins (*n* = 2023) included in the NAPPArray identified in each plasma samples included in the study from 7 healthy donors and 50 sCRC patients.

Sample ID	Diagnosis	N° of aAb	% of aAb
1	sCRC	862	43
2	sCRC	846	42
3	sCRC	822	41
4	sCRC	191	9
5	sCRC	110	5
6	sCRC	466	23
7	sCRC	609	30
8	sCRC	832	41
9	sCRC	704	35
10	sCRC	285	14
11	sCRC	497	25
12	sCRC	851	42
13	sCRC	249	12
14	sCRC	717	35
15	sCRC	227	11
16	sCRC	646	32
17	sCRC	1040	51
18	sCRC	447	22
19	sCRC	593	29
20	sCRC	174	9
21	sCRC	1228	61
22	sCRC	1288	64
23	sCRC	471	23
24	sCRC	1225	61
25	sCRC	615	30
26	sCRC	458	23
27	sCRC	521	26
28	sCRC	864	43
29	sCRC	266	13
30	sCRC	1150	57
31	sCRC	813	40
32	sCRC	608	30
33	sCRC	671	33
34	sCRC	1141	56
35	sCRC	700	35
36	sCRC	381	19
37	sCRC	1310	65
38	sCRC	217	11
39	sCRC	327	16
40	sCRC	562	28
41	sCRC	541	27
42	sCRC	755	37
43	sCRC	557	28
44	sCRC	415	21
45	sCRC	720	36
46	sCRC	93	5
47	sCRC	363	18
48	sCRC	505	25
49	sCRC	48	2
50	sCRC	381	19
51	Healthy donor	715	35
52	Healthy donor	398	20
53	Healthy donor	880	43
54	Healthy donor	1149	57
55	Healthy donor	229	11
56	Healthy donor	278	14
57	Healthy donor	345	17

**Table 3:** aAbs profile against 67 TAAs with statistical significance (*p* < 0.01) that might discriminate healthy donors (negative colonoscopy) (*n* = 7) vs. sCRC patients (*n* = 50). None of these TAAs have been detected as positive aAb in healthy donors.

TAA ID	*p*-Value (U-Mann-Whitney)	N° sCRC (*n* = 50) with aAb Positive	Fold Change (FC)	
			Median FC sCRC	% sCRC > FC1
TEX11	0.0005	8/50	2.6	90
GORASP2	0.0007	2/50	3.1	82
C9orf80	0.0007	3/50	3.9	92
HMOX2	0.0008	13/50	3.0	92
KIF9	0.0008	14/50	2.7	92
ICAM2	0.0012	16/50	2.4	94
MARCKSL1	0.0012	10/50	2.4	88
RB1	0.0012	2/50	2.6	92
SPP1	0.0012	3/50	2.8	86
STC2	0.0013	10/50	2.0	84
BHMT2	0.0014	10/50	2.2	86
D21S2056E	0.0016	10/50	2.2	84
DLAT	0.0016	2/50	2.3	86
CKMT1B	0.0017	27/50	2.6	86
GTF2H1	0.0017	8/50	2.3	82
ALDOA	0.0019	8/50	2.0	88
COX11	0.0020	14/50	2.4	88
RPL11	0.0020	2/50	2.4	86
ASB3	0.0020	8/50	2.3	84
PRCP	0.0024	21/50	1.9	86
PDEF	0.0024	10/50	2.2	86
ANP32A	0.0024	2/50	2.3	82
GNAI3	0.0026	5/50	2.5	86
HBG1	0.0026	2/50	3.5	84
ARHI	0.0028	11/50	1.9	86
RCV1	0.0028	3/50	2.9	86
RAB8A	0.0028	5/50	2.4	84
GDEP	0.0030	11/50	2.2	82
PLAC1	0.0030	7/50	2.0	84
HSD17B3	0.0030	2/50	2.3	80
SH3BP1	0.0033	3/50	2.0	84
USP5	0.0033	2/50	2.1	82
TCEAL1	0.0033	5/50	2.5	84
KPNA6	0.0035	18/50	2.3	84
Progranulin	0.0038	4/50	1.8	86
CHODL	0.0038	4/50	2.2	86
KCNE2	0.0041	13/50	2.0	82
SERPINA5	0.0041	5/50	2.2	80
SLCO4A1	0.0041	4/50	2.5	80
SDPR	0.0041	10/50	1.8	86
JUP	0.0041	3/50	2.5	82
FRK	0.0041	6/50	2.1	84
DDX39	0.0044	2/50	2.1	80
PSAP	0.0048	2/50	2.1	84
SCARB1	0.0048	2/50	2.6	84
AMY2A	0.0052	9/50	2.1	84
BECN1	0.0056	16/50	2.0	84
ST14	0.0056	16/50	1.7	80
LDHB	0.0056	8/50	2.3	84
SNX10	0.0056	2/50	2.1	82
PSTPIP1	0.0060	17/50	2.1	84
SLC6A1	0.0065	6/50	2.0	78
HM13	0.0070	10/50	1.7	84
RHOH	0.0070	7/50	1.9	82
KIF22	0.0075	5/50	2.4	78
SYTL1	0.0075	4/50	2.1	80
Mage3	0.0075	10/50	1.9	82
HIST1H3D	0.0075	4/50	2.3	82
RWDD1	0.0080	9/50	2.1	80
RILP	0.0080	6/50	2.0	76
RPL35	0.0080	4/50	1.9	82
PLAGL1	0.0086	21/50	1.6	82
DDX56	0.0086	9/50	1.7	84
CTNNA1	0.0086	9/50	2.2	82
BET1	0.0086	4/50	2.0	78
HLA-DOB	0.0093	28/50	1.6	80
HSPC047	0.0093	4/50	1.9	80

**Table 4:** Functional Enrichment Analysis of detected TAAs with statistical significance (*p* > 0.05) between healthy donors vs. sCRC; non-metastatic sCRC vs. synchronic metastasis sCRC.

Healthy Donors vs. Pathological sCCR					
GO Term	Function	*p*-Value	TAAs Number	% TAA	TAAs ID
GO:0006974	Response to DNA damage stimulus	0.0011	7	14.6	SUMO1, UBE2A, CCNH, GTF2H4, MLH1, GTSE1, RAD17
GO:0006281	DNA repair	0.0020	6	12.5	SUMO1, UBE2A, CCNH, GTF2H4, MLH1, RAD17
GO:0033554	Cellular response to stress	0.0087	7	14.6	SUMO1, UBE2A, CCNH, GTF2H4, MLH1, GTSE1, RAD17
GO:0006259	DNA metabolic process	0.0051	7	14.6	SUMO1, UBE2A, CCNH, GTF2H4, MLH1, IGF1, RAD17
GO:0006950	Response to stress	0.0053	13	27.1	UBE2A, CCNH, GTF2H4, LYZ, MLH1, IGF1, SMAD1, GTSE1, SUMO1, DARC, CA2, ENTPD2, RAD17
GO:0051716	Cellular response to stimulus	0.0143	8	16.7	SUMO1, UBE2A, CCNH, GTF2H4, MLH1, SMAD1, GTSE1, RAD17
GO:0009056	Catabolic process	0.0158	10	20.8	ALDOA, SUMO1, UBE2A, CCNH, LYZ, GTF2H4, MLH1, SAE1, PSME3, ENTPD2
GO:0006367	Transcription initiation from RNA polymerase II promoter	0.098	3	6.3	MED4, CCNH, GTF2H4
GO:0044265	Cellular macromolecule catabolic process	0.0266	7	14.6	SUMO1, UBE2A, CCNH, GTF2H4, MLH1, SAE1, PSME3
GO:0042770	DNA damage response, signal transduction	0.0268	3	6.3	MLH1, GTSE1, RAD17
GO:0016070	RNA metabolic process	0.0278	8	16.7	MED4, CCNH, GTF2H4, MLH1, WBP11, SMAD1, SCGB1A1, RPS7
GO:0006352	Transcription initiation	0.0287	3	6.3	MED4, CCNH, GTF2H4
GO:0009892	Negative regulation of metabolic process	0.0362	7	14.6	RPS26, SUMO1, MLH1, PSME3, SMAD1, SCGB1A1, RAD17
GO:0010605	Negative regulation of macromolecule metabolic process	0.0280	7	14.6	RPS26, SUMO1, MLH1, PSME3, SMAD1, SCGB1A1, RAD17
GO:0009057	Macromolecule catabolic process	0.0364	7	14.6	SUMO1, UBE2A, CCNH, GTF2H4, MLH1, SAE1, PSME3
GO:0006414	Translational elongation	0.0411	3	6.3	RPS26, RPL35, RPS7
GO:0044248	Cellular catabolic process	0.0418	8	16.7	SUMO1, UBE2A, CCNH, GTF2H4, MLH1, SAE1, PSME3, ENTPD2
GO:0014902	Myotube differentiation	0.0498	2	4.2	CAST, IGF1
GO:0030901	Midbrain development	0.0498	2	4.2	SMAD1, PITX3
**Non-metastatic sCRC vs. Synchronic Metastasis sCRC**					
**GO term**	**Function**	***p*-value**	**Aab number**	**% Aab**	**Aab ID**
GO:0007548	Sex differentiation	0.0209	3	13.0	HSD17B3, EIF2B2, TEX11
GO:0045137	Development of primary sexual characteristics	0.0151	3	13.0	HSD17B3, EIF2B2, TEX11
GO:0006833	Water transport	0.0236	2	8.7	AQP5, AQP3
GO:0042044	Fluid transport	0.0250	2	8.7	AQP5, AQP3
GO:0048513	Organ development	0.0366	7	30.4	PFN1, CRIP2, FHL3, HSD17B3, EIF2B2, TEX11, TIMP1

## Data Availability

Available at https://gredos.usal.es/handle/10366/3676 (accessed on 26 March 2021).

## Supplementary Materials

The following are available online at https://www.mdpi.com/article/10.3390/cancers13112718/s1, Figure S1: Schematic description of experimental workflow of serum screening, Figure S2: Reproducibility NAPPArray performance, Figure S3: The Human Protein Atlas [28] showed that VTI2 (**A**), p53 (**B**), MARCKSL1 (**C**), HMOX2 (**D**), DLAT (**E**), RSP28 (**F**) have been described as a CRC prognosis biomarkers, Table S1: List of TAA proteins displayed in the NAPPArrays used in this study, Table S2: Positive antibodies for EBNA spots (*n* = 14) defined taking into account the normalization values (> mean of the normalized values of pANT7_cGST spots + 3SD), Table S3: aAbs profile against TAAs that exhibited implication to discriminate healthy donors (*n* = 7) vs. sCRC patients (*n* = 50). None of these TAAs have been identified as positive aAb in healthy donors, Table S4: aAbs profile against TAAs with statistical significance (*p* < 0.01) that might distinguish metastatic sCRC (*n* = 12) vs. Non-metastatic sCRC (*n* = 38). None of these TAAs have been identified as positive aAb in healthy donors, Table S5: Results from leave-one-out validation when the lasso algorithm selects only one variable, Table S6: Identified proteins as potential biomarker panel of aAbs in sCRC that appear in < 10% of agarose-based immunoprecipitation registered experiments in CRAPome.
