# Supplementary material for: Tracking the Antibody Immunome in Sporadic Colorectal Cancer by Using Antigen Self-Assembled Protein Arrays
Source: Cancers (Basel). 2021 May 31;13(11):2718. doi: 10.3390/cancers13112718 (PMC8198956; doi:10.3390/cancers13112718)
Supplement: Supplementary file 1 [file cancers-13-02718-s001.zip › cancers-1179288-supplementary.pdf]

Supplementary Material: Tracking the Antibody Immunome in Sporadic Colorectal Cancer by Using Antigen Self-Assembled Protein Arrays

María González-González, José María Sayagués, Luis Muñoz-Bellvís, Carlos Eduardo Pedreira, Marcello L.R. de Campos, Jacinto García, José Antonio Alcázar, Patrick F. Braz, Breno L. Galves, Luis Miguel González, Oscar Bengoechea, María del Mar Abad, Juan Jesús Cruz, Lorena Bellido, Emilio Fonseca, Paula Díez, Pablo Juanes-Velasco, Alicia Landeira-Viñuela, Quentin Lecrevisse, Enrique Montalvillo, Rafael Góngora, Oscar Blanco, José Manuel Sanchez-Santos, Joshua LaBaer, Alberto Orfao and Manuel Fuentes

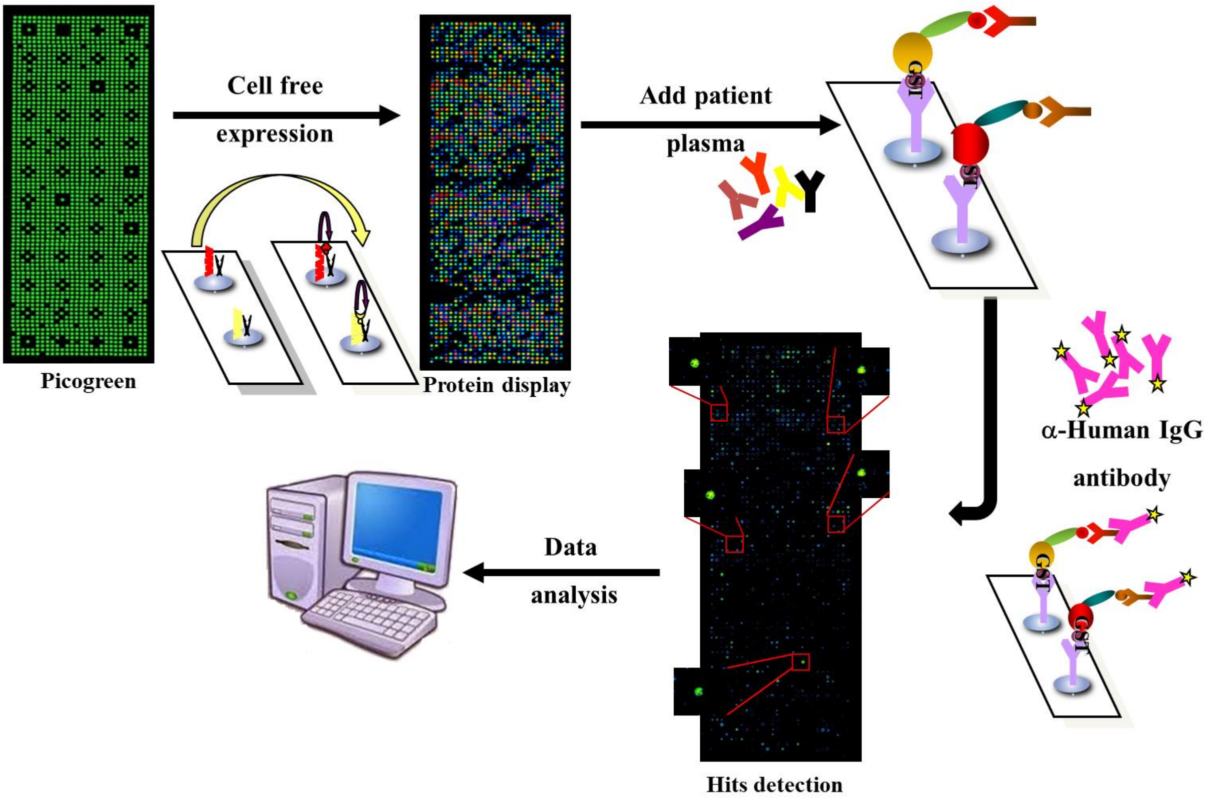

Figure S1. Schematic description of experimental workflow of serum screening.

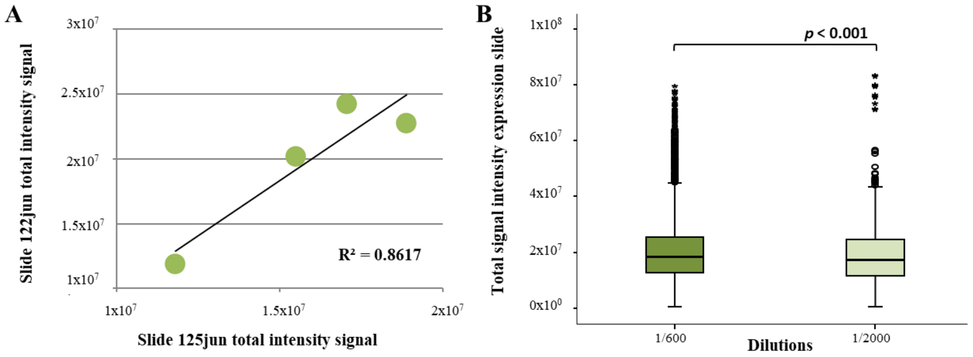

Figure S2. Reproducibility NAPPArray performance.

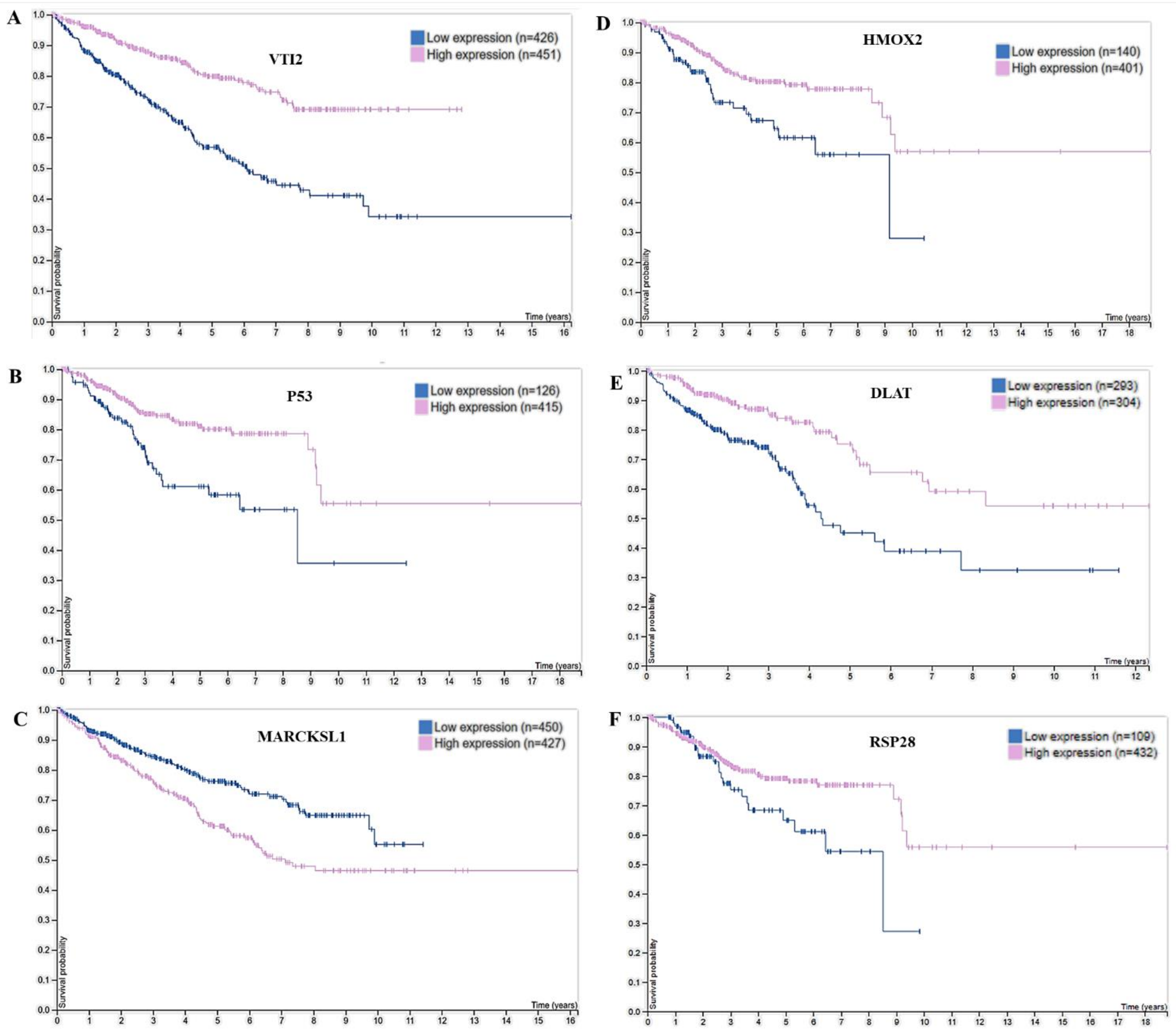

Figure S3. The Human Protein Atlas [28] showed that VTI2 (**A**), p53 (**B**), MARCKSL1 (**C**), HMOX2 (**D**), DLAT (**E**), RSP28 (**F**) have been described as a CRC prognosis biomarkers.

Table S1. List of tumoral antigen human proteins included in NAPPA

Clone name	Start Position	Stop Position	Chromosomal Localization	Description
76P	43661419	43699293	15q15.3	Gamma-tubulin complex component 4
A4GALT	43088127	43117304	22q13.2	Lactosylceramide 4-alpha-galactosyltransferase
AARS	70286198	70323446	16q22.1	Alanine--tRNA ligase, cytoplasmic
AATF	35306175	35414171	17q12	Protein AATF
ABAT	8768422	8878432	16p13.2	4-aminobutyrate aminotransferase, mitochondrial
ABCD4	74752126	74769759	14q24.3	ATP-binding cassette sub-family D member 4
ABCF1	30539153	30564956	6p21.33	ATP-binding cassette sub-family F member 1
ABCF2	150904923	150924316	7q36.1	ATP-binding cassette sub-family F member 2
ABCG2	89011416	89152474	4q22.1	ATP-binding cassette sub-family G member 2
ABHD11	73150424	73153197	7q11.23	Alpha/beta hydrolase domain-containing protein 11
ABHD5	43731605	43775863	3p21.33	1-acylglycerol-3-phosphate O-acyltransferase ABHD5
ACAA2	47309869	47340330	18q21.1	3-ketoacyl-CoA thiolase, mitochondrial
ACAD8	134123389	134135749	11q25	Isobutyryl-CoA dehydrogenase, mitochondrial
ACAD9	128598439	128634910	3q21.3	Acyl-CoA dehydrogenase family member 9, mitochondrial
ACADM	76190036	76253260	1p31.1	Medium-chain specific acyl-CoA dehydrogenase, mitochondrial
ACO2	41865129	41924993	22q13.2	Aconitate hydratase, mitochondrial
ACPP	132036211	132087142	3q22.1	Prostatic acid phosphatase
ACSL5	114133776	114188138	10q25.2	Long-chain-fatty-acid--CoA ligase 5
ACTA1	229566992	229569845	1q42.13	Actin, alpha skeletal muscle
ACTG2	74119441	74146992	2p13.1	Actin, gamma-enteric smooth muscle
ACTN1	69340860	69446157	14q24.1	Alpha-actinin-1
ACTN4	39138289	39222223	19q13.2	Alpha-actinin-4
ACTR1A	104238986	104262482	10q24.32	Alpha-centractin
ACTR1B	98272431	98280570	2q11.2	Beta-centractin
ACTRT2	2938046	2939465	1p36.32	Actin-related protein T2
ACY1	52009066	52023199	3p21.2	Aminoacylase-1
ADA	43248163	43280874	20q13.12	Adenosine deaminase
ADAM2	39601254	39695808	8p11.22	Disintegrin and metalloproteinase domain-containing protein 2
ADAT1	75630879	75657198	16q23.1	tRNA-specific adenosine deaminase 1
ADCK4	41197434	41224112	19q13.2	AarF domain-containing protein kinase 4
ADFP	19108373	19149288	9p22.1	Perilipin-2
ADH5	99992132	100009952	4q23	Alcohol dehydrogenase class-3
ADORA3	112025970	112106584	1p13.2	Adenosine receptor A3
ADRBK1	67033881	67054027	11q13.2	Beta-adrenergic receptor kinase 1
AGA	178351924	178363657	4q34.3	N(4)-(beta-N-acetylglucosaminyl)-L-asparaginase
AGER	32148745	32152101	6p21.32	Advanced glycosylation end product-specific receptor
AGMAT	15898848	15911605	1p36.21	Agmatinase, mitochondrial
AGPAT5	6565878	6617184	8p23.1	1-acyl-sn-glycerol-3-phosphate acyltransferase epsilon
AGTR2	115301975	115306225	Xq23	Type-2 angiotensin II receptor
AGTRAP	11796141	11814859	1p36.22	Type-1 angiotensin II receptor-associated protein
AIPL1	6297013	6338519	17p13.2	Aryl-hydrocarbon-interacting protein-like 1
AK1	130628759	130640022	9q34.11	Adenylate kinase isoenzyme 1
AK3L1	4711155	4742043	9p24.1	GTP:AMP phosphotransferase AK3, mitochondrial
AKAP10	19807615	19881656	17p11.2	A-kinase anchor protein 10, mitochondrial
AKR1B1	134127102	134144036	7q33	Aldose reductase

AKR1B10	134212344	134226160	7q33	Aldo-keto reductase family 1 member B10
AKR1C4	5237425	5260912	10p15.1	Aldo-keto reductase family 1 member C4
AKT1	105235686	105262088	14q32.33	RAC-alpha serine/threonine-protein kinase
AKT3	243651535	244014381	1q43	RAC-gamma serine/threonine-protein kinase
ALB	74262831	74287129	4q13.3	Serum albumin
ALDH1A2	58245622	58790065	15q21.3	Retinal dehydrogenase 2
ALDH3A2	19551449	19580911	17p11.2	Fatty aldehyde dehydrogenase
ALDH3B1	67776048	67796744	11q13.2	Aldehyde dehydrogenase family 3 member B1
ALDH4A1	19197926	19229275	1p36.13	Delta-1-pyrroline-5-carboxylate dehydrogenase, mitochondrial
ALDOA	30064411	30081778	16p11.2	Fructose-bisphosphate aldolase A
ALF	38462442	38471278	1p34.3	Four and a half LIM domains protein 3
ALG2	101978708	101984238	9q22.33	Alpha-1,3/1,6-mannosyltransferase ALG2
ALG3	183960089	183967336	3q27.1	Dol-P-Man:Man(5)GlcNAc(2)-PP-Dol alpha-1,3-mannosyltransferase
ALG9	111652919	111742305	11q23.1	Alpha-1,2-mannosyltransferase ALG9
AMY2A	104159999	104168402	1p21.1	Pancreatic alpha-amylase
ANGPTL7	11249398	11256038	1p36.22	Angiopoietin-related protein 7
ANKH	14704910	14871887	5p15.2	Progressive ankylosis protein homolog
ANKRA2	72848160	72861511	5q13.2	Ankyrin repeat family A protein 2
ANKRD1	92671853	92681033	10q23.31	Ankyrin repeat domain-containing protein 1
ANP32A	69070874	69113236	15q23	Acidic leucine-rich nuclear phosphoprotein 32 family member A
ANXA1	75766673	75785309	9q21.13	Annexin A1
ANXA11	81910645	81965328	10q22.3	Annexin A11
ANXA13	124693034	124749647	8q24.13	Annexin A13
ANXA5	122589110	122618268	4q27	Annexin A5
ANXA8	48255253	48279200	10q11.22	Annexin A8
ANXA9	150954493	150968110	1q21.3	Annexin A9
AP2A2	924894	1012239	11p15.5	AP-2 complex subunit alpha-2
AP2B1	33905065	34053436	17q12	AP-2 complex subunit beta
AP3S1	115177178	115249778	5q22.3	AP-3 complex subunit sigma-1
AP3S2	90373831	90437574	15q26.1	AP-3 complex subunit sigma-2
APBB2	40812044	41218731	4p13	Amyloid beta A4 precursor protein-binding family B member 2
APEH	49711435	49721396	3p21.31	Acylamino-acid-releasing enzyme
APEX1	20923350	20925927	14q11.2	DNA-(apurinic or apyrimidinic site) lyase
APEX2	55026790	55035490	Xp11.21	DNA-(apurinic or apyrimidinic site) lyase 2
APOA1	116706467	116708666	11q23.3	Apolipoprotein A-I
APOA2	161192082	161193421	1q23.3	Apolipoprotein A-II
APOD	195295573	195311076	3q29	Apolipoprotein D
APOE	45409011	45412650	19q13.32	Apolipoprotein E
APOH	64208151	64252643	17q24.2	Beta-2-glycoprotein 1
APOL2	36622256	36636000	22q12.3	Apolipoprotein L2
APPBP1	66836778	66907159	16q22.1	NEDD8-activating enzyme E1 regulatory subunit
AQP2	50344524	50352664	12q13.12	Aquaporin-2
AQP3	33441152	33447609	9p13.3	Aquaporin-3
AQP5	50355653	50359464	12q13.12	Aquaporin-5
AQP5	50355653	50359464	12q13.12	Aquaporin-5
AQP9	58430368	58478110	15q21.3	Aquaporin-9
ARAF	47420516	47431307	Xp11.23	Serine/threonine-protein kinase A-Raf
ARAF1	47420516	47431307	Xp11.23	Serine/threonine-protein kinase A-Raf
ARC	143692405	143696833	8q24.3	Activity-regulated cytoskeleton-associated protein
ARFGAP3	43192508	43254112	22q13.2	ADP-ribosylation factor GTPase-activating protein 3
ARFIP2	6496910	6502666	11p15.4	Arfaptin-2

ARG1	131894284	131905472	6q23.2	Arginase-1
ARHA	49396578	49450431	3p21.31	Transforming protein RhoA
ARHB	20646835	20649200	2p24.1	Rho-related GTP-binding protein RhoB
ARHC	113243728	113250056	1p13.2	Rho-related GTP-binding protein RhoC
ARHG	3848208	3862213	11p15.4	Rho-related GTP-binding protein RhoG
ARHGDIB	15094951	15114662	12p12.3	Rho GDP-dissociation inhibitor 2
ARHGEF1	42387228	42434302	19q13.2	Rho guanine nucleotide exchange factor 1
ARHGEF5	144052381	144077725	7q35	Rho guanine nucleotide exchange factor 5
ARHI	68511645	68517314	1p31.3	GTP-binding protein Di-Ras3
ARL1	101786898	101801598	12q23.2	ADP-ribosylation factor-like protein 1
ARL3	104433488	104474164	10q24.32	ADP-ribosylation factor-like protein 3
ARL5A	152645498	152685006	2q23.3	ADP-ribosylation factor-like protein 5A
ARL5B	18948334	18970568	10p12.31	ADP-ribosylation factor-like protein 5B
ARMCX2	100910267	100914876	Xq22.1	Armadillo repeat-containing X-linked protein 2
ARMCX3	100877787	100882833	Xq22.1	Armadillo repeat-containing X-linked protein 3
ARPM1	169484709	169487683	3q26.2	Actin-related protein T3
ARRB2	4613784	4624794	17p13.2	Beta-arrestin-2
ART3	76932337	77033955	4q21.1	Ecto-ADP-ribosyltransferase 3
ASB3	53759810	54087170	2p16.2	Ankyrin repeat and SOCS box protein 3
ASCL1	103351464	103354294	12q23.2	Achaete-scute homolog 1
ASGR1	7076750	7082883	17p13.1	Asialoglycoprotein receptor 1
ASGR2	7004641	7019019	17p13.1	Asialoglycoprotein receptor 2
ASNS	97481430	97501854	7q21.3	Asparagine synthetase [glutamine-hydrolyzing]
ASPA	3375668	3406713	17p13.2	Aspartoacylase
ASPSCR1	79934683	79975282	17q25.3	Tether containing UBX domain for GLUT4
ATF3	212738676	212794119	1q32.3	Cyclic AMP-dependent transcription factor ATF-3
ATF4	39915700	39918691	22q13.1	Cyclic AMP-dependent transcription factor ATF-4
ATG10	81267844	81572676	5q14.1	Ubiquitin-like-conjugating enzyme ATG10
ATG3	112251356	112280893	3q13.2	Ubiquitin-like-conjugating enzyme ATG3
ATG4C	63249806	63331184	1p31.3	Cysteine protease ATG4C
ATIC	216176540	216214487	2q35	Bifunctional purine biosynthesis protein PURH
ATOH7	69990386	69991871	10q21.3	Protein atonal homolog 7
ATP5B	57031959	57039798	12q13.3	ATP synthase subunit beta, mitochondrial
ATP5G2	54026510	54071192	12q13.13	ATP synthase F(0) complex subunit C2, mitochon-drial
ATP6V0D1	67471917	67515140	16q22.1	V-type proton ATPase subunit d 1
ATXN10	46067678	46241187	22q13.31	Ataxin-10
AUH	93976097	94124195	9q22.31	Methylglutaconyl-CoA hydratase, mitochondrial
AURKA	54944445	54967393	20q13.2	Aurora kinase A
AVPI1	99437181	99447080	10q24.2	Arginine vasopressin-induced protein 1
AYP1	38059189	38069245	14q21.1	Hepatocyte nuclear factor 3-alpha
B3GNT1	66112843	66115163	11q13.2	Beta-1,4-glucuronyltransferase 1
B4-2	89790470	89794879	6q15	Proline-rich nuclear receptor coactivator 1
B4GALT3	161141100	161147287	1q23.3	Beta-1,4-galactosyltransferase 3
B4GALT7	177027101	177037348	5q35.3	Beta-1,4-galactosyltransferase 7
BAAT	104122699	104145801	9q31.1	Bile acid-CoA:amino acid N-acyltransferase
BACE1	117156402	117186975	11q23.3	Beta-secretase 1
BAD	64037302	64052176	11q13.1	Bcl2-associated agonist of cell death
BAG1	33247818	33264761	9p13.3	BAG family molecular chaperone regulator 1
BAG3	121410882	121437331	10q26.11	BAG family molecular chaperone regulator 3
BAIAP2	79008948	79091232	17q25.3	Brain-specific angiogenesis inhibitor 1-associated protein 2
BAK1	33540329	33548019	6p21.31	Bcl-2 homologous antagonist/killer
BANP	87982850	88110924	16q24.2	Protein BANP
BAP1	52435029	52444366	3p21.1	Ubiquitin carboxyl-terminal hydrolase BAP1

BASP1	17065707	17276943	5p15.1	Brain acid soluble protein 1
BAT4	31629006	31634060	6p21.33	G patch domain and ankyrin repeat-containing protein 1
BATF	75988768	76013358	14q24.3	Basic leucine zipper transcriptional factor ATF-like
BAX	49458072	49465055	19q13.33	Apoptosis regulator BAX
BBOX1	27062272	27149356	11p14.2	Gamma-butyrobetaine dioxygenase
BBS2	56500748	56554195	16q12.2	Bardet-Biedl syndrome 2 protein
BBS4	72978527	73030817	15q24.1	Bardet-Biedl syndrome 4 protein
BC-2	59062933	59066491	19q13.43	Charged multivesicular body protein 2a
BCAS2	115110178	115124260	1p13.2	Pre-mRNA-splicing factor SPF27
BCAT2	49298319	49314286	19q13.33	Branched-chain-amino-acid aminotransferase, mitochondrial
BCL10	85731931	85742773	1p22.3	B-cell lymphoma/leukemia 10
BCL2	60790579	60987361	18q21.33	Apoptosis regulator Bcl-2
BCL2A1	80253231	80263788	15q25.1	Bcl-2-related protein A1
BCL2L1	30252255	30311792	20q11.21	Bcl-2-like protein 1
BCL2L13	18111621	18213388	22q11.21	Bcl-2-like protein 13
BECN1	40962152	40985367	17q21.31	Beclin-1
BET1	93592074	93633694	7q21.3	BET1 homolog
BGN	152760397	152775012	Xq28	Biglycan
BHMT2	78365540	78385289	5q14.1	S-methylmethionine--homocysteine S-methyltransferase BHMT2
BIK	43506754	43525718	22q13.2	Bcl-2-interacting killer
BIRC5	76210267	76221717	17q25.3	Baculoviral IAP repeat-containing protein 5
BIRC7	61867235	61871859	20q13.33	Baculoviral IAP repeat-containing protein 7
BLZF1	169337208	169365778	1q24.2	Golgin-45
BMP10	69092613	69098649	2p13.3	Bone morphogenetic protein 10
BMP5	55618443	55740362	6p12.1	Bone morphogenetic protein 5
BMP7	55743804	55841685	20q13.31	Bone morphogenetic protein 7
BMPR1A	88516407	88692595	10q23.2	Bone morphogenetic protein receptor type-1A
BNIP2	59951345	59981733	15q22.2	BCL2/adenovirus E1B 19 kDa protein-interacting protein 2
BOK	242498136	242513546	2q37.3	Bcl-2-related ovarian killer protein
BPIL1	31595406	31611515	20q11.21	BPI fold-containing family B member 2
BRE	28112808	28561768	2p23.2	BRCA1-A complex subunit BRE
BTBD1	83685174	83736106	15q25.2	BTB/POZ domain-containing protein 1
BTBD14A	138898383	138987131	9q34.3	Nucleus accumbens-associated protein 2
BTF3	72794233	72801460	5q13.2	Transcription factor BTF3
BTG1	92536286	92539673	12q21.33	Protein BTG1
BUD13	116618886	116643704	11q23.3	BUD13 homolog
BZW1	201675317	201688569	2q33.1	Basic leucine zipper and W2 domain-containing protein 1
BZW2	16685756	16746148	7p21.1	Basic leucine zipper and W2 domain-containing protein 2
C10orf33	100143322	100174941	10q24.2	Pyridine nucleotide-disulfide oxidoreductase domain-containing protein 2
C11orf64	60383209	60454622	11q12.2	Putative uncharacterized protein encoded by LINC00301
C11orf73	86013253	86056969	11q14.2	Protein Hikeshi
C12orf24	110906169	110928190	12q24.11	Protein FAM216A
C15orf48	45,430,529	45,448,761	15q21.1	Chromosome 15 Open Reading Frame 48
C19orf28	3538259	3574288	19p13.3	Major facilitator superfamily domain-containing protein 12
C19orf43	12841454	12845589	19p13.2	Uncharacterized protein C19orf43
C19orf6	1009647	1021117	19p13.3	Membralin

C1orf128	24104895	24114722	1p36.11	PITH domain-containing protein 1
C1orf162	112016414	112021134	1p13.2	Transmembrane protein C1orf162
C1orf182	156307105	156316786	1q22	TSSK6-activating co-chaperone protein
C1orf48	212899495	212965124	1q32.3	Kinetochore-associated protein NSL1 homolog
C1orf62	109358520	109506106	1p13.3	Protein AKNAD1
C1QB	22979255	22988031	1p36.12	Complement C1q subcomponent subunit B
C1QTNF1	77018896	77045870	17q25.3	Complement C1q tumor necrosis factor-related protein 1
C1QTNF2	159774758	159797648	5q33.3	Complement C1q tumor necrosis factor-related protein 2
C1QTNF6	37576207	37595425	22q12.3	Complement C1q tumor necrosis factor-related protein 6
C21orf66	34106210	34144169	21q22.11	PAX3- and PAX7-binding protein 1
C2orf15	99757948	99939204	2q11.2	Uncharacterized protein C2orf15
C2orf30	54014181	54045956	2p16.2	Endoplasmic reticulum lectin 1
C2orf4	32090129	32236299	2p22.3	Protein MEMO1
C3AR1	8210898	8219067	12p13.31	C3a anaphylatoxin chemotactic receptor
C3orf37	128997671	129025029	3q21.3	Embryonic stem cell-specific 5-hydroxymethylcytosine-binding protein
C4BPA	207277607	207318317	1q32.2	C4b-binding protein alpha chain
C5AR1	47793280	47825323	19q13.32	C5a anaphylatoxin chemotactic receptor 1
C5orf15	133,955,502	133,968,787	5q31.1	Chromosome 5 Open Reading Frame 15
C6	41142336	41261540	5p13.1	Complement component C6
C6orf55	142468367	142545826	6q24.1	Vacuolar protein sorting-associated protein VTA1 homolog
C9orf116	138387027	138393580	9q34.3	UPF0691 protein C9orf116
C9orf156	100666771	100684852	9q22.33	Nef-associated protein 1
C9orf45	123,109,494	123,115,477	9q33.3	Chromosome 9 Open Reading Frame 45
C9orf80	115446206	115480516	9q32	SOSS complex subunit C
CA12	63613577	63674360	15q22.2	Carbonic anhydrase 12
CA2	86376081	86393722	8q21.2	Carbonic anhydrase 2
CABP1	121078355	121105127	12q24.31	Calcium-binding protein 1
CACNG4	64961026	65029514	17q24.2	Voltage-dependent calcium channel gamma-4 subunit
CACNG6	54495542	54515923	19q13.42	Voltage-dependent calcium channel gamma-6 subunit
CAGE1	7326889	7389976	6p24.3	Cancer-associated gene 1 protein
CALM2	47387221	47403740	2p21	Calmodulin
CALML3	5566924	5568225	10p15.1	Calmodulin-like protein 3
CALU	128379346	128411861	7q32.1	Calumenin
CAMK1D	12391481	12877545	10p13	Calcium/calmodulin-dependent protein kinase type 1D
CAMKK1	3763609	3798185	17p13.2	Calcium/calmodulin-dependent protein kinase kinase 1
CAMKK2	121675497	121736111	12q24.31	Calcium/calmodulin-dependent protein kinase kinase 2
CAMKV	49895421	49907655	3p21.31	CaM kinase-like vesicle-associated protein
CAPN1	64948037	64979477	11q13.1	Calpain-1 catalytic subunit
CAPN10	241526133	241557122	2q37.3	Calpain-10
CART	71014990	71016875	5q13.2	Cocaine- and amphetamine-regulated transcript protein
CASP2	142985308	143004789	7q34	Caspase-2
CASP3	185548850	185570663	4q35.1	Caspase-3
CASP4	104813593	104840163	11q22.3	Caspase-4
CASP6	110609785	110624739	4q25	Caspase-6

CASQ1	160160285	160171676	1q23.2	Calsequestrin-1
CASQ2	116242628	116311402	1p13.1	Calsequestrin-2
CAST	95860971	96115299	5q15	Calpastatin
CBLB	105374305	105588396	3q13.11	E3 ubiquitin-protein ligase CBL-B
CBR1	37442239	37445464	21q22.12	Carbonyl reductase [NADPH] 1
CBR1	37442239	37445464	21q22.12	Carbonyl reductase [NADPH] 1
CBR1	37442239	37445464	21q22.12	Carbonyl reductase [NADPH] 1
CBR3	37507210	37518864	21q22.12	Carbonyl reductase [NADPH] 3
CBX6	39257455	39268319	22q13.1	Chromobox protein homolog 6
CCDC102A	57546090	57570511	16q21	Coiled-coil domain-containing protein 102A
CCDC104	55,519,595	55,545,879	2p16.1	Cilia And Flagella Associated Protein 36
CCDC107	35658301	35661508	9p13.3	Coiled-coil domain-containing protein 107
CCDC16	33288549	33290205	17q12	Zinc finger protein 830
CCDC47	61822610	61853711	17q23.3	Coiled-coil domain-containing protein 47
CCDC72	48481667	48485616	3p21.31	Translation machinery-associated protein 7
CCDC86	60609544	60618554	11q12.2	Coiled-coil domain-containing protein 86
CCL11	32612687	32615353	17q12	Eotaxin
CCL13	32683471	32685629	17q12	C-C motif chemokine 13
CCL20	228678558	228682272	2q36.3	C-C motif chemokine 20
CCL27	34661877	34664045	9p13.3	C-C motif chemokine 27
CCL5	34198495	34207797	17q12	C-C motif chemokine 5
CCND1	69455855	69469242	11q13.3	G1/S-specific cyclin-D1
CCND2	4382938	4414516	12p13.32	G1/S-specific cyclin-D2
CCND3	41902671	42018095	6p21.1	G1/S-specific cyclin-D3
CCNE1	30302805	30315215	19q12	G1/S-specific cyclin-E1
CCNH	86687311	86708836	5q14.3	Cyclin-H
CCNI	77968311	77997158	4q21.1	Cyclin-I
CCR6	167525295	167553184	6q27	C-C chemokine receptor type 6
CCS	66360292	66373490	11q13.2	Copper chaperone for superoxide dismutase
CCT3	156278759	156337664	1q22	T-complex protein 1 subunit gamma
CCT5	10250033	10266524	5p15.2	T-complex protein 1 subunit epsilon
CCT7	73460548	73480149	2p13.2	T-complex protein 1 subunit eta
CD151	832843	839831	11p15.5	CD151 antigen
CD160	145695798	145715614	1q21.1	CD160 antigen
CD19	28943260	28950667	16p11.2	B-lymphocyte antigen CD19
CD207	71057347	71062952	2p13.3	C-type lectin domain family 4 member K
CD2BP2	30362087	30366682	16p11.2	CD2 antigen cytoplasmic tail-binding protein 2
CD34	208057594	208084747	1q32.2	Hematopoietic progenitor cell antigen CD34
CD36	79998891	80308593	7q21.11	Platelet glycoprotein 4
CD37	49838428	49846592	19q13.33	Leukocyte antigen CD37
CD40	44746911	44758502	20q13.12	Tumor necrosis factor receptor superfamily member 5
CD44	35160417	35253949	11p13	CD44 antigen
CD48	160648536	160681641	1q23.3	CD48 antigen
CD59	33719807	33757991	11p13	CD59 glycoprotein
CD63	56119107	56123491	12q13.2	CD63 antigen
CD69	9905082	9913497	12p13.31	Early activation antigen CD69
CD7	80272744	80275478	17q25.3	T-cell antigen CD7
CD74	149781200	149792492	5q32	HLA class II histocompatibility antigen gamma chain
CD82	44585977	44641913	11p11.2	CD82 antigen
CDC2	50796310	50883179	14q21.3	Cyclin-dependent kinase-like 1
CDC20	43824626	43828874	1p34.2	Cell division cycle protein 20 homolog
CDC27	45195069	45266788	17q21.32	Cell division cycle protein 27 homolog
CDC34	531714	542097	19p13.3	Ubiquitin-conjugating enzyme E2 R1

CDC42	22379120	22419437	1p36.12	Cell division control protein 42 homolog
CDC42EP1	37956454	37965412	22q13.1	Cdc42 effector protein 1
CDC42EP2	65082289	65089900	11q13.1	Cdc42 effector protein 2
CDC45L	19466982	19508135	22q11.21	Cell division control protein 45 homolog
CDC5L	44355262	44418163	6p21.1	Cell division cycle 5-like protein
CDCA1	163236366	163325554	1q23.3	Kinetochore protein Nuf2
CDCA4	105475910	105487485	14q32.33	Cell division cycle-associated protein 4
CDCA5	64833772	64851636	11q13.1	Sororin
CDH15	89238175	89261900	16q24.3	Cadherin-15
CDIPT	29869678	29875057	16p11.2	CDP-diacylglycerol--inositol 3-phosphatidyltransfe-rase
CDK2	56360553	56366568	12q13.2	Cyclin-dependent kinase 2
CDK4	58141510	58149796	12q14.1	Cyclin-dependent kinase 4
CDK5	150750899	150755617	7q36.1	Cyclin-dependent-like kinase 5
CDK6	92234235	92465908	7q21.2	Cyclin-dependent kinase 6
CDKN1A	36644305	36655116	6p21.2	Cyclin-dependent kinase inhibitor 1
CDKN1B	12867992	12875305	12p13.1	Cyclin-dependent kinase inhibitor 1B
CDKN2A	21967751	21995300	9p21.3	Cyclin-dependent kinase inhibitor 2A
CDKN2B	22002902	22009362	9p21.3	Cyclin-dependent kinase 4 inhibitor B
CDKN2C	51426417	51440305	1p32.3	Cyclin-dependent kinase 4 inhibitor C
CDSN	31127873	31133179	6p22.1	Corneodesmosin
CEACAM8	43084393	43099207	19q13.2	Carcinoembryonic antigen-related cell adhesion molecule 8
CEBPG	33864236	33873592	19q13.11	CCAAT/enhancer-binding protein gamma
CENPH	68485375	68506184	5q13.2	Centromere protein H
CENPK	64813593	64858998	5q12.3	Centromere protein K
CES1	55836763	55867249	16q12.2	Liver carboxylesterase 1
CES2	66968347	66978999	16q22.1	Cocaine esterase
CFB	31895475	31919861	6p21.33	Complement factor B
CFHR1	196788887	196801319	1q31.3	Complement factor H-related protein 1
CFHR2	196788898	196928356	1q31.3	Complement factor H-related protein 2
CFP	47483612	47489704	Xp11.23	Properdin
CH25H	90965694	90967071	10q23.31	Cholesterol 25-hydroxylase
CHEK1	125495036	125546150	11q24.2	Serine/threonine-protein kinase Chk1
CHEK2	29083731	29138410	22q12.1	Serine/threonine-protein kinase Chk2
CHMP2A	59062933	59066491	19q13.43	Charged multivesicular body protein 2a
CHMP5	33264940	33281977	9p13.3	Charged multivesicular body protein 5
CHODL	19273580	19639690	21q21.1	Chondrolectin
CHRAC1	141521397	141527236	8q24.3	Chromatin accessibility complex protein 1
CHRM1	62676151	62689279	11q12.3	Muscarinic acetylcholine receptor M1
CHRNB1	7348380	7361026	17p13.1	Acetylcholine receptor subunit beta
CHST12	2443223	2474242	7p22.3	Carbohydrate sulfotransferase 12
CHST8	34112861	34264414	19q13.11	Carbohydrate sulfotransferase 8
CIB1	90773207	90777279	15q26.1	Calcium and integrin-binding protein 1
CILP	65488337	65503826	15q22.31	Cartilage intermediate layer protein 1
CITED1	71521488	71527037	Xq13.1	Cbp/p300-interacting transactivator 1
CITED2	139693393	139695757	6q24.1	Cbp/p300-interacting transactivator 2
CKAP1	36605191	36616849	19q13.12	Tubulin-folding cofactor B
CKLF	66586466	66600154	16q21	Chemokine-like factor
CKMT1B	43885252	43897099	15q15.3	Creatine kinase U-type, mitochondrial
CKN1	60169658	60240900	5q12.1	DNA excision repair protein ERCC-8
CKS1B	154947129	154951725	1q21.3	Cyclin-dependent kinases regulatory subunit 1
CLCC1	109472130	109506111	1p13.3	Chloride channel CLIC-like protein 1
CLCF1	67131639	67141648	11q13.2	Cardiotrophin-like cytokine factor 1
CLDN1	190023490	190040264	3q28	Claudin-1

CLEC1A	10222153	10264226	12p13.2	C-type lectin domain family 1 member A
CLEC2B	10005583	10022735	12p13.31	C-type lectin domain family 2 member B
CLIC1	31698358	31707540	6p21.33	Chloride intracellular channel protein 1
CLIC2	154505500	154563966	Xq28	Chloride intracellular channel protein 2
CLIC5	45868045	46048132	6p21.1	Chloride intracellular channel protein 5
CLNS1A	77225981	77348850	11q14.1	Methylosome subunit pICln
CLPP	6361463	6368919	19p13.3	ATP-dependent Clp protease proteolytic subunit, mitochondrial
CLSTN1	9789084	9884584	1p36.22	Calsyntenin-1
CLU	27454434	27472548	8p21.1	Clusterin
CMKOR1	237476430	237491001	2q37.3	Atypical chemokine receptor 3
CNIH4	224544552	224567161	1q42.11	Protein cornichon homolog 4
CNN2	1026298	1039068	19p13.3	Calponin-2
CNN3	95362507	95392834	1p21.3	Calponin-3
CNOT10	32726637	32815367	3p22.3	CCR4-NOT transcription complex subunit 10
CNP	40118759	40129749	17q21.2	2',3'-cyclic-nucleotide 3'-phosphodiesterase
CNTROB	7835419	7853236	17p13.1	Centrobin
COG1	71189129	71204646	17q25.1	Conserved oligomeric Golgi complex subunit 1
COMT	19929130	19957498	22q11.21	Catechol O-methyltransferase
COPB1	14464986	14521573	11p15.2	Coatomer subunit beta
COPE	19010323	19030206		Coatomer subunit epsilon
COPS5	67955314	67996018	8q13.1	COP9 signalosome complex subunit 5
COPS6	99686577	99689823	7q22.1	COP9 signalosome complex subunit 6
COPZ2	46103533	46115392	17q21.32	Coatomer subunit zeta-2
COQ2	84182689	84206067	4q21.23	4-hydroxybenzoate polyprenyltransferase, mito-chondrial
COQ6	74416629	74430373	14q24.3	Ubiquinone biosynthesis monooxygenase COQ6, mitochondrial
CORO1B	67205519	67211292	11q13.2	Coronin-1B
COX11	53029263	53046146	17q22	Cytochrome c oxidase assembly protein COX11, mi-tochondrial
COX4I1	85832239	85840650	16q24.1	Cytochrome c oxidase subunit 4 isoform 1, mito-chondrial
COX5B	98262503	98264846	2q11.2	Cytochrome c oxidase subunit 5B, mitochondrial
COX6A1	120875893	120878545	12q24.31	Cytochrome c oxidase subunit 6A1, mitochondrial
COX6A1	120875893	120878545	12q24.31	Cytochrome c oxidase subunit 6A1, mitochondrial
COX6A2	31439052	31439967	16p11.2	Cytochrome c oxidase subunit 6A2, mitochondrial
COX6B1	36139125	36149763	19q13.12	Cytochrome c oxidase subunit 6B1
COX7C	85913721	85916779	5q14.3	Cytochrome c oxidase subunit 7C, mitochondrial
CP110	19535133	19564730	16p12.3	Centriolar coiled-coil protein of 110 kDa
CPSF1	145618444	145634753	8q24.3	Cleavage and polyadenylation specificity factor subunit 1
CPT1C	50194155	50216988	19q13.33	Carnitine O-palmitoyltransferase 1, brain isoform
CRABP2	156669398	156675608	1q23.1	Cellular retinoic acid-binding protein 2
CRADD	94071151	94288616	12q22	Death domain-containing protein CRADD
CREB1	208394461	208468155	2q33.3	Cyclic AMP-responsive element-binding protein 1
CREB3L1	46299212	46342972	11p11.2	Cyclic AMP-responsive element-binding protein 3-like protein 1
CREG1	167498914	167523004	1q24.2	Protein CREG1
CRIP1	105952654	105955284	14q32.33	Cysteine-rich protein 1
CRIP2	105939299	105946499	14q32.33	Cysteine-rich protein 2
CRK	1323983	1366456	17p13.3	Adapter molecule crk
CRMP1	5749811	5894785	4p16.2	Dihydropyrimidinase-related protein 1
CRTAP	33155471	33189265	3p22.3	Cartilage-associated protein
CRYAB	111779289	111794446	11q23.1	Alpha-crystallin B chain

CSDA	10851683	10875911	12p13.2	Y-box-binding protein 3
CSF1	110452864	110473614	1p13.3	Macrophage colony-stimulating factor 1
CSF3	38171614	38174066	17q21.1	Granulocyte colony-stimulating factor
CSH2	61949372	61951126	17q23.3	Chorionic somatomammotropin hormone 2
CSNK1A1L	37677398	37679803	13q13.3	Casein kinase I isoform alpha-like
CSNK1E	38686697	38794527	22q13.1	Casein kinase I isoform epsilon
CSNK2A2	58191811	58231824	16q21	Casein kinase II subunit alpha'
CSNK2B	31633013	31638120	6p21.33	Casein kinase II subunit beta
CSRP2BP	18118762	18169031	20p11.23	Cysteine-rich protein 2-binding protein
CST3	23608534	23619110	20p11.21	Cystatin-C
CST6	65779312	65780976	11q13.1	Cystatin-M
CSTF1	54967427	54979518	20q13.2	Cleavage stimulation factor subunit 1
CSTF2	100075384	100095921	Xq22.1	Cleavage stimulation factor subunit 2
CTBP1	1205236	1243741	4p16.3	C-terminal-binding protein 1
CTGF	132269316	132272513	6q23.2	Connective tissue growth factor
CTNNA1	137946656	138270723	5q31.2	Catenin alpha-1
CTNS	3539762	3564836	17p13.2	Cystinosin
CTSC	88026760	88070955	11q14.2	Dipeptidyl peptidase 1
CTSG	25042728	25045466	14q12	Cathepsin G
CTSH	79213400	79241916	15q25.1	Pro-cathepsin H
CTSL	90340434	90346308	9q21.33	Cathepsin L1
CTSZ	57570240	57582302	20q13.32	Cathepsin Z
CUL4B	119658464	119709649	Xq24	Cullin-4B
CXADR	18884700	18965897	21q21.1	Coxsackievirus and adenovirus receptor
CXCL1	74735110	74736959	4q13.3	Growth-regulated alpha protein
CXCL10	76942273	76944650	4q21.1	C-X-C motif chemokine 10
CXCL11	76954835	76962568	4q21.1	C-X-C motif chemokine 11
CXCL2	74962752	74965010	4q13.3	C-X-C motif chemokine 2
CXCL5	74861359	74864496	4q13.3	C-X-C motif chemokine 5
CXCL6	74702214	74714781	4q13.3	C-X-C motif chemokine 6
CXCR3	70835766	70838367	Xq13.1	C-X-C chemokine receptor type 3
CXorf20	18181051	18239024	Xp22.13	BEN domain-containing protein 2
CXXC5	139026884	139063467	5q31.2	CXXC-type zinc finger protein 5
CYBA	88709691	88717560	16q24.3	Cytochrome b-245 light chain
CYC1	145149930	145152428	8q24.3	Cytochrome c1, heme protein, mitochondrial
CycA1				
CycB1				
CycB2				
CYCD1				
CycE2	95891998	95908906	8q22.1	G1/S-specific cyclin-E2
CYCS	25159710	25164980	7p15.3	Cytochrome c
CYFIP2	156693089	156822606	5q33.3	Cytoplasmic FMR1-interacting protein 2
CYP11A1	74630100	74660081	15q24.1	Cholesterol side-chain cleavage enzyme, mitochondrial
CYP1A1	75011883	75017951	15q24.1	Cytochrome P450 1A1
CYP2C8	96796530	96829254	10q23.33	Cytochrome P450 2C8
CYP4B1	47223510	47285085	1p33	Cytochrome P450 4B1
CYP4F11	16023177	16045677	19p13.12	Phylloquinone omega-hydroxylase CYP4F11
CYR61	86046444	86049645	1p22.3	Protein CYR61
D21S2056E	45209394	45225174	21q22.3	Ribosomal RNA processing protein 1 homolog A
DAD1	23033805	23058175	14q11.2	Dolichyl-diphosphooligosaccharide--protein glycosyltransferase subunit DAD1
DAPP1	100737990	100791311	4q23	Dual adapter for phosphotyrosine and 3-phosphotyrosine and 3-phosphoinositide
DARC	159173097	159176290	1q23.2	Atypical chemokine receptor 1

DC2	52558386	52569070	3p21.1	5'-nucleotidase domain-containing protein 2
DCBLD2	98514785	98620533	3q12.1	Discoidin, CUB and LCCL domain-containing protein 2
DCC1	120846216	120868250	8q24.12	Sister chromatid cohesion protein DCC1
DCK	71858255	71896631	4q13.3	Deoxycytidine kinase
DCN	91539025	91576900	12q21.33	Decorin
DCP1A	53317447	53381654	3p21.1	mRNA-decapping enzyme 1A
DCT	95089558	95131936	13q32.1	L-dopachrome tautomerase
DCTN3	34613548	34620515	9p13.3	Dynactin subunit 3
DDOST	20978270	20988000	1p36.12	Dolichyl-diphosphooligosaccharide--protein glycosyltransferase 48 kDa subunit
DDT	24313554	24322660	22q11.23	D-dopachrome decarboxylase
DDX21	70715884	70744829	10q22.1	Nucleolar RNA helicase 2
DDX23	49223547	49246625	12q13.12	Probable ATP-dependent RNA helicase DDX23
DDX24	94517266	94547591	14q32.12	ATP-dependent RNA helicase DDX24
DDX39	14519631	14530192	19p13.12	ATP-dependent RNA helicase DDX39A
DDX50	70661034	70706603	10q22.1	ATP-dependent RNA helicase DDX50
DDX56	44605016	44614650	7p13	Probable ATP-dependent RNA helicase DDX56
DES	220283099	220291461	2q35	Desmin
DGAT1	145539954	145550573	8q24.3	Diacylglycerol O-acyltransferase 1
DHCR24	55315306	55352891	1p32.3	Delta(24)-sterol reductase
DHFR	79922047	79950802	5q14.1	Dihydrofolate reductase
DHRS3	12627939	12677737	1p36.22	Short-chain dehydrogenase/reductase 3
DHRSX	2137557	2420846	Xp22.33	Dehydrogenase/reductase SDR family member on chromosome X
DHX16	30620896	30640814	6p21.33	Putative pre-mRNA-splicing factor ATP-dependent RNA helicase DHX16
DHX32	127524906	127585005	10q26.2	Putative pre-mRNA-splicing factor ATP-dependent RNA helicase DHX32
DIABLO	122692210	122712081	12q24.31	Diablo homolog, mitochondrial
DIDO1	61509090	61569304	20q13.33	Death-inducer obliterator 1
DIRAS3	68511645	68517314	1p31.3	GTP-binding protein Di-Ras3
DKC1	153991031	154005964	Xq28	H/ACA ribonucleoprotein complex subunit 4
DKFZP564B147	134184962	134186226	Xq26.3	Protein FAM127B
DKK3	11984653	12031316	11p15.3	Dickkopf-related protein 3
DLAT	111895538	111935114	11q23.1	Dihydrolipoyllysine-residue acetyltransferase component of pyruvate dehydrogenase complex
DMAP1	44679127	44686353	1p34.1	DNA methyltransferase 1-associated protein 1
DNAJB1	14625582	14640582	19p13.12	DnaJ homolog subfamily B member 1
DNAJB4	78444859	78483648	1p31.1	DnaJ homolog subfamily B member 4
DNAJB9	108210012	108215294	7q31.1	DnaJ homolog subfamily B member 9
DNAJC7	40128451	40173394	17q21.2	DnaJ homolog subfamily C member 7
DNASE1	3661729	3730144	16p13.3	Deoxyribonuclease-1
DNASE1L3	58177984	58200424	3p14.3	Deoxyribonuclease gamma
DNM1L	32832134	32898486	12p11.21	Dynamin-1-like protein
DNPEP	220238268	220264744	2q35	Aspartyl aminopeptidase
DNTT	98064085	98098321	10q24.1	DNA nucleotidylexotransferase
DOC-1R	67,506,490	67,508,728	11q13.2	Cyclin-Dependent Kinase 2 Associated Protein 2
DOK4	57505863	57521239	16q21	Docking protein 4
DOM3Z	31937587	31940069	6p21.33	Decapping and exoribonuclease protein
DPM2	130697378	130700763	9q34.11	Dolichol phosphate-mannose biosynthesis regulatory protein
DPP7	140004994	140009629	9q34.3	Dipeptidyl peptidase 2
DPP9	4675236	4724685	19p13.3	Dipeptidyl peptidase 9
DPPA4	109044988	109056419	3q13.13	Developmental pluripotency-associated protein 4

DRD2	113280318	113346413	11q23.2	D(2) dopamine receptor
DRD5	9783258	9785632	4p16.1	D(1B) dopamine receptor
DRG1	31795509	31924726	22q12.2	Developmentally-regulated GTP-binding protein 1
DSCR2	40546695	40555777	21q22.2	Proteasome assembly chaperone 1
DSTN	17550508	17590564	20p12.1	Destrin
DTX2	76090993	76135312	7q11.23	Probable E3 ubiquitin-protein ligase DTX2
DTYMK	242615157	242626406	2q37.3	Thymidylate kinase
DUSP11	73989311	74007284	2p13.1	RNA/RNP complex-1-interacting phosphatase
DUSP12	161719548	161727028	1q23.3	Dual specificity protein phosphatase 12
DUSP13	76854192	76868979	10q22.2	Dual specificity protein phosphatase 13 isoform A
DUSP14	35849937	35873603	17q12	Dual specificity protein phosphatase 14
DUSP19	183943287	183964733	2q32.1	Dual specificity protein phosphatase 19
DUSP22	291630	351355	6p25.3	Dual specificity protein phosphatase 22
DUSP6	89741009	89747048	12q21.33	Dual specificity protein phosphatase 6
DUT	48623208	48635570	15q21.1	Deoxyuridine 5'-triphosphate nucleotidohydrolase, mitochondrial
DVL3	183873176	183891398	3q27.1	Segment polarity protein dishevelled homolog DVL-3
DYNLT1	159057506	159065771	6q25.3	Dynein light chain Tctex-type 1
DYRK1B	40315990	40324841	19q13.2	Dual specificity tyrosine-phosphorylation-regulated kinase 1B
DYRK4	4671370	4723325	12p13.32	Dual specificity tyrosine-phosphorylation-regulated kinase 4
EBAG9	110551940	110578225	8q23.2	Receptor-binding cancer antigen expressed on SiSo cells
EBI2	99946784	99959659	13q32.3	G-protein coupled receptor 183
EBP	48379546	48387104	Xp11.23	3-beta-hydroxysteroid-Delta(8),Delta(7)-isomerase
ECGF1	50964181	50968485	22q13.33	Thymidine phosphorylase
ECM1	150480538	150486265	1q21.3	Extracellular matrix protein 1
ECRG4	106679702	106694615	2q12.2	Augurin
EDEM1	5229331	5261642	3p26.1	ER degradation-enhancing alpha-mannosidase-like protein 1
EDF1	139756571	139760738	9q34.3	Endothelial differentiation-related factor 1
EDG1	101702444	101707074	1p21.2	Sphingosine 1-phosphate receptor 1
EDG3	91606362	91619925	9q22.1	Sphingosine 1-phosphate receptor 3
EEF1A1	74225473	74233520	6q13	Elongation factor 1-alpha 1
EEF1A2	62119366	62130505	20q13.33	Elongation factor 1-alpha 2
EEFSEC	127872297	128127485	3q21.3	Selenocysteine-specific elongation factor
EFEMP1	56093102	56151274	2p16.1	EGF-containing fibulin-like extracellular matrix protein 1
EFNB1	68048840	68061990	Xq13.1	Ephrin-B1
EFTUD2	42927311	42977030	17q21.31	116 kDa U5 small nuclear ribonucleoprotein component
EHD2	48216600	48246391	19q13.33	EH domain-containing protein 2
EHD4	42190950	42264776	15q15.1	EH domain-containing protein 4
EI24	125439112	125454575	11q24.	Etoposide-induced protein 2.4 homolog
EIF1	39845137	39848920	17q21.2	Eukaryotic translation initiation factor 1
EIF2A	150264465	150302029	3q25.1	Eukaryotic translation initiation factor 2A
EIF2B2	75469614	75476292	14q24.3	Translation initiation factor eIF-2B subunit beta
EIF2S3	24072833	24096088	Xp22.11	Eukaryotic translation initiation factor 2 subunit 3
EIF3S2	32687529	32697205	1p35.1	Eukaryotic translation initiation factor 3 subunit I
EIF3S3	117654369	117779164	8q23.3	Eukaryotic translation initiation factor 3 subunit H
EIF3S6	109213445	109447562	8q23.1	Eukaryotic translation initiation factor 3 subunit E
EIF3S7	36906897	36925483	22q12.3	Eukaryotic translation initiation factor 3 subunit D
EIF3S8	28390900	28415200	16p11.2	Eukaryotic translation initiation factor 3 subunit C

EIF4A1	7476024	7482323	17p13.1	Eukaryotic initiation factor 4A-I
EIF4E	99792835	99851788	4q23	Eukaryotic translation initiation factor 4E
EIF4ENIF1	31832963	31892094	22q12.2	Eukaryotic translation initiation factor 4E transporter
EIF5A	7210318	7215774	17p13.1	Eukaryotic translation initiation factor 5A-1
ELA3A	22303514	22325135	1p36.12	Chymotrypsin-like elastase family member 3B
ELAC1	48494361	48514491	18q21.2	Zinc phosphodiesterase ELAC protein 1
ELAVL2	23690102	23826335	9p21.3	ELAV-like protein 2
ELF3	201977073	201986316	1q32.1	ETS-related transcription factor Elf-3
ELF5	34500340	34535352	11p13	ETS-related transcription factor Elf-5
ELK1	47494920	47510003	Xp11.23	ETS domain-containing protein Elk-1
ELL2	95220802	95297775	5q15	RNA polymerase II elongation factor ELL2
ELP4	31531297	31805546	11p13	Elongator complex protein 4
EML1	100204030	100408397	14q32.2	Echinoderm microtubule-associated protein-like 1
EMP1	13349650	13369708	12p13.1	Epithelial membrane protein 1
ENG	130577291	130617035	9q34.11	Endoglin
ENO1	8921061	8939308	1p36.23	Alpha-enolase
ENO3	4851387	4860426	17p13.2	Beta-enolase
ENPP2	120569326	120685693	8q24.12	Ectonucleotide pyrophosphatase/phosphodiesterase family member 2
ENSA	150573327	150602088	1q21.3	Alpha-endosulfine
ENTPD2	139942550	139948497	9q34.3	Ectonucleoside triphosphate diphosphohydrolase 2
EPB49	21906506	21940038	8p21.3	Dematin
EPHA2	16450832	16482582	1p36.13	Ephrin type-A receptor 2
EPHX1	225997794	226033260	1q42.12	Epoxide hydrolase 1
EPHX2	27348296	27403081	8p21.1	Bifunctional epoxide hydrolase 2
EPS8	15773092	16035263	12p12.3	Epidermal growth factor receptor kinase substrate 8
EPSTI1	43460524	43566407	13q14.11	Epithelial-stromal interaction protein 1
ERAL1	27181956	27188085	17q11.2	GTPase Era, mitochondrial
ERCC1	45910591	45982086	19q13.32	DNA excision repair protein ERCC-1
ERCC5	103497194	103528345	13q33.1	DNA repair protein complementing XP-G cells
EREG	75230860	75254468	4q13.3	Proepiregulin
ERF	42751724	42759309	19q13.2	ETS domain-containing transcription factor ERF
ESAM	124622026	124632186	11q24.2	Endothelial cell-selective adhesion molecule
ET	128,458,761	128,587,558	11q24.3	V-Ets Avian Erythroblastosis Virus E26 Oncogene Homolog 1
ETR101	13261229	13265722	19p13.2	Immediate early response gene 2 protein
ETS1	128328656	128457453	11q24.3	Protein C-ets-1
ETS2	40177231	40196879	21q22.2	Protein C-ets-2
ETV3	157090983	157108266	1q23.1	ETS translocation variant 3
EXDL1	41474923	41522941	15q15.1	Exonuclease 3'-5' domain-containing protein 1
EXOD1	20791515	20911671	16p12.3	ERI1 exoribonuclease 2
EXOSC3	37766975	37801434	9p13.2	Exosome complex component RRP40
F2R	76011868	76031606	5q13.3	Proteinase-activated receptor 1
F3	94994781	95007356	1p21.3	Tissue factor
FABP1	88422510	88427635	2p11.2	Fatty acid-binding protein, liver
FABP3	31838472	31849697	1p35.2	Fatty acid-binding protein, heart
FABP7	123100620	123105219	6q22.31	Fatty acid-binding protein, brain
FAIM	138327448	138352218	3q22.3	Fas apoptotic inhibitory molecule 1
FAM103A1	83654959	83659809	15q25.2	RNMT-activating mini protein
FAM107A	58549844	58613337	3p14.2	Protein FAM107A
FAM13C1	61005890	61122939	10q21.1	Protein FAM13C
FAM48A	37583449	37633850	13q13.3	Transcription factor SPT20 homolog
FAM76B	95502106	95523573	11q21	Protein FAM76B
FAP	163027194	163101661	2q24.2	Prolyl endopeptidase FAP

FARS1	5261277	5771813	6p25.1	Phenylalanine--tRNA ligase, mitochondrial
FAS	90750414	90775542	10q23.31	Tumor necrosis factor receptor superfamily member 6
FASLG	172628154	172636014	1q24.3	Tumor necrosis factor ligand superfamily member 6
FATE1	150884507	150891666	Xq28	Fetal and adult testis-expressed transcript protein
FBL	40325098	40337054	19q13.2	rRNA 2'-O-methyltransferase fibrillarin
FBXL13	102453308	102715286	7q22.1	F-box/LRR-repeat protein 13
FBXL5	15606162	15683302	4p15.32	F-box/LRR-repeat protein 5
FBXL8	67193834	67198473	16q22.1	F-box/LRR-repeat protein 8
FBXO2	11708424	11715842	1p36.22	F-box only protein 2
FBXO5	153291664	153304714	6q25.2	F-box only protein 5
FBXW11	171288553	171433877	5q35.1	F-box/WD repeat-containing protein 11
FBXW2	123514256	123555690	9q33.2	F-box/WD repeat-containing protein 2
FCGR2A	161475220	161493803	1q23.3	Low affinity immunoglobulin gamma Fc region receptor II-a
FCN1	137801431	137809809	9q34.3	Ficolin-1
FCN3	27695603	27701315	1p36.11	Ficolin-3
FCRL3	157644111	157670647	1q23.1	Fc receptor-like protein 3
FCRLM1	161676762	161684142	1q23.3	Fc receptor-like A
FDPS	155278539	155290457	1q22	Farnesyl pyrophosphate synthase
FEM1C	114856608	114880591	5q22.3	Protein fem-1 homolog C
FEN1	61560109	61564716	11q12.2	Flap endonuclease 1
FEV	219845809	219850379	2q35	Protein FEV
FFAR3	35849362	35851387	19q13.12	Free fatty acid receptor 3
FGD2	36973422	36996846	6p21.2	FYVE, RhoGEF and PH domain-containing protein 2
FGF10	44303646	44389808	5p12	Fibroblast growth factor 10
FGF13	137713735	138304939	Xq26.3	Fibroblast growth factor 13
FGF16	76709648	76712769	Xq21.1	Fibroblast growth factor 16
FGF18	170846660	170884627	5q35.1	Fibroblast growth factor 18
FGF21	49258816	49261587	19q13.33	Fibroblast growth factor 21
FGF7	49715293	49780972	15q21.2	Fibroblast growth factor 7
FGFR1	38268656	38326352	8p11.22	Fibroblast growth factor receptor 1
FGFR1OP	167412670	167466201	6q27	FGFR1 oncogene partner
FGFR4	176513887	176525145	5q35.2	Fibroblast growth factor receptor 4
FGFRL1	1003724	1020685	4p16.3	Fibroblast growth factor receptor-like 1
FHIT	59735036	61237133	3p14.2	Bis(5'-adenosyl)-triphosphatase
FHL2	105974169	106054970	2q12.1	Four and a half LIM domains protein 2
FHL3	38462442	38471278	1p34.3	Four and a half LIM domains protein 3
FHL5	97010424	97064512	6q16.1	Four and a half LIM domains protein 5
FKBP10	39968932	39979465	17q21.2	Peptidyl-prolyl cis-trans isomerase FKBP10
FKBP14	30050203	30066300	7p14.3	Peptidyl-prolyl cis-trans isomerase FKBP14
FKBP3	45584803	45604522	14q21.2	Peptidyl-prolyl cis-trans isomerase FKBP3
FKBP4	2904119	2914576	12p13.33	Peptidyl-prolyl cis-trans isomerase FKBP4
FKBP5	35541362	35696360	6p21.31	Peptidyl-prolyl cis-trans isomerase FKBP5
FKBP8	18642561	18654887	19p13.11	Peptidyl-prolyl cis-trans isomerase FKBP8
FKBPL	32096484	32098068	6p21.33	FK506-binding protein-like
FKHL18	30432103	30433420	20q11.21	Forkhead box protein S1
FKRP	47249303	47280245	19q13.32	Fukutin-related protein
FLI1	128556430	128683162	11q24.3	Friend leukemia integration 1 transcription factor
FLOT2	27206353	27224697	17q11.2	Flotillin-2
FLRT3	14303634	14318262	20p12.1	Leucine-rich repeat transmembrane protein FLRT3
FMO3	171060018	171086959	1q24.3	Dimethylaniline monooxygenase [N-oxide-forming] 3
FN1	216225163	216300895	2q35	Fibronectin

FOLH1	49168187	49230222	11p11.12	Glutamate carboxypeptidase 2
FOLR1	71900602	71907345	11q13.4	Folate receptor alpha
FOS	75745477	75748933	14q24.3	Proto-oncogene c-Fos
FOSB	45971253	45978437	19q13.32	Protein fosB
FOSL1	65659520	65668044	11q13.1	Fos-related antigen 1
FOSL2	28615315	28640179	2p23.2	Fos-related antigen 2
FRAT2	99092255	99094458	10q24.1	GSK-3-binding protein FRAT2
FRK	116252312	116381921	6q22.1	Tyrosine-protein kinase FRK
FSCN1	5632439	5646286	7p22.1	Fascin
FST	52776239	52782964	5q11.2	Follistatin
FTH1	61727190	61735132	11q12.3	Ferritin heavy chain
FTL	49468558	49470135	19q13.33	Ferritin light chain
FTSJ3	61896793	61907372	17q23.3	pre-rRNA processing protein FTSJ3
FUBP1	78409740	78444794	1p31.1	Far upstream element-binding protein 1
FUT11	75532049	75540009	10q22.2	Alpha-(1,3)-fucosyltransferase 11
FXYD2	117671559	117699413	11q23.3	Sodium/potassium-transporting ATPase subunit gamma
FXYD7	35634154	35645204	19q13.12	FXYD domain-containing ion transport regulator 7
G0S2	209848765	209849733	1q32.2	G0/G1 switch protein 2
G1P2	948803	949920	1p36.33	Ubiquitin-like protein ISG15
G3BP	151150606	151192346	5q33.1	Ras GTPase-activating protein-binding protein 1
GABARAP	7143333	7146089	17p13.1	Gamma-aminobutyric acid receptor-associated protein
GABRA3	151334706	151619830	Xq28	Gamma-aminobutyric acid receptor subunit alpha-3
GADD45A	68150744	68154021	1p31.3	Growth arrest and DNA damage-inducible protein GADD45 alpha
GAGE2	49354132	49361430	Xp11.23	G antigen 2A
GAGE4	-	-	Xp11.2-p11.4	G antigen 4
GAGEB1	49452053	49460596	Xp11.23	P antigen family member 1
GAL3ST1	30950622	30970574	22q12.2	Galactosylceramide sulfotransferase
GALC	88304164	88460009	14q31.3	Galactocerebrosidase
GALE	24122089	24127271	1p36.11	UDP-glucose 4-epimerase
GALNACT-2	43633934	43680756	10q11.21	Chondroitin sulfate N-acetylgalactosaminyltransferase 2
GALNT6	51745031	51786651	12q13.13	Polypeptide N-acetylgalactosaminyltransferase 6
GAP43	115342171	115440337	3q13.31	Neuromodulin
GAPDH	6643093	6647537	12p13.31	Glyceraldehyde-3-phosphate dehydrogenase
GARS	30634297	30673649	7p14.3	Glycine--tRNA ligase
GAS7	9813926	10101868	17p13.1	Growth arrest-specific protein 7
GATA3	8095567	8117161	10p14	Trans-acting T-cell-specific transcription factor GATA-3
GBAS	56019486	56067874	7p11.2	Protein NipSnap homolog 2
GBL	27265232	27293490	2p23.3	Cytosolic carboxypeptidase-like protein 5
GBP5	89724633	89738544	1p22.2	Guanylate-binding protein 5
GC20	40351175	40353915	3p22.1	Eukaryotic translation initiation factor 1b
GCG	162999392	163008914	2q24.2	Glucagon
GCH1	55308726	55369570	14q22.2	GTP cyclohydrolase 1
GCLM	94350761	94374966	1p22.1	Glutamate--cysteine ligase regulatory subunit
GDB	138824815	138853226	9q34.3	Ubiquitin-associated domain-containing protein 1
GDBR1				
GDEP	79,827,471	79,877,770	4q21.21	Prostate Cancer Associated Transcript 4
GDF3	7842378	7848372	12p13.31	Growth/differentiation factor 3
GDNF	37812779	37839788	5p13.2	Glial cell line-derived neurotrophic factor
GENX-3414	77172886	77232752	4q21.1	Starch-binding domain-containing protein 1
GFI1	92940319	92952433	1p22.1	Zinc finger protein Gfi-1

GGA2	23474863	23533316	16p12.2	ADP-ribosylation factor-binding protein GGA2
GGN	38874905	38878722	19q13.2	Gametogenetin
GGT1	24979718	25024972	22q11.23	Gamma-glutamyltranspeptidase 1
GH2	61957578	61959295	17q23.3	Growth hormone variant
GIF	59596741	59612974	11q12.1	Gastric intrinsic factor
GIPC1	14588572	14606944	19p13.12	PDZ domain-containing protein GIPC1
GJA1	121756838	121770873	6q22.31	Gap junction alpha-1 protein
GJA5	147228332	147245484	1q21.2	Gap junction alpha-5 protein
GJB1	70435044	70445366	Xq13.1	Gap junction beta-1 protein
GJB2	20761609	20767037	13q12.11	Gap junction beta-2 protein
GK	30671476	30748725	Xp21.2	Glycerol kinase
GLRX	95087023	95158709	5q15	Glutaredoxin-1
GLTSCR2	48248779	48260315	19q13.33	Glioma tumor suppressor candidate region gene 2 protein
GLUL	182350839	182361341	1q25.3	Glutamine synthetase
GLYAT	58407899	58499447	11q12.1	Glycine N-acyltransferase
GM2A	150591711	150650001	5q33.1	Ganglioside GM2 activator
GMDS	1624041	2245926	6p25.3	GDP-mannose 4,6 dehydratase
GMFB	54941202	54955914	14q22.2	Glia maturation factor beta
GMFG	39818993	39833012	19q13.2	Glia maturation factor gamma
GNA13	63006833	63052957	17q24.1	Guanine nucleotide-binding protein subunit alpha-13
GNAI2	50263724	50296787	3p21.31	Guanine nucleotide-binding protein G(i) subunit alpha-2
GNAI3	110091233	110136975	1p13.3	Guanine nucleotide-binding protein G(k) subunit alpha
GNAT2	110145889	110155679	1p13.3	Guanine nucleotide-binding protein G(t) subunit alpha-2
GNB2L1	180663909	180675096	5q35.3	Guanine nucleotide-binding protein subunit beta-2-like 1
GNB3	6949118	6956557	12p13.31	Guanine nucleotide-binding protein G(I)/G(S)/G(T) subunit beta-3
GNG10	114423615	114432526	9q31.3	Guanine nucleotide-binding protein G(I)/G(S)/G(O) subunit gamma-10
GNG11	93551011	93557922	7q21.3	Guanine nucleotide-binding protein G(I)/G(S)/G(O) subunit gamma-11
GNG3	62475130	62476673	11q12.3	Guanine nucleotide-binding protein G(I)/G(S)/G(O) subunit gamma-3
GNGT1	93220885	93540577	7q21.3	Guanine nucleotide-binding protein G(T) subunit gamma-T1
GNL1	30509154	30524951	6p21.33	Guanine nucleotide-binding protein-like 1
GNLY	85912298	85925977	2p11.2	Granulysin
GNRH1	25276776	25282170	8p21.2	Progonadoliberin-1
GORASP2	171784974	171823639	2q31.1	Golgi reassembly-stacking protein 2
GOSR2	45000483	45105003	17q21.32	Golgi SNAP receptor complex member 2
GPBP1L1	46092976	46153785	1p34.1	Vasculin-like protein 1
GPC3	132669773	133119922	Xq26.2	Glypican-3
GPC4	132434131	132549518	Xq26.2	Glypican-4
GPHN	66974125	67648520	14q23.3	Gephyrin
GPIAP1	34073230	34122703	11p13	Caprin-1
GPM6B	13789150	13956757	Xp22.2	Neuronal membrane glycoprotein M6-b
GPR114	57576333	57625593	16q21	Probable G-protein coupled receptor 114
GPR146	1084212	1098897	7p22.3	Probable G-protein coupled receptor 146
GPR157	9160364	9189229	1p36.23	Probable G-protein coupled receptor 157
GPR17	128403439	128410213	2q14.3	Uracil nucleotide/cysteinyl leukotriene receptor

GPR173	53078273	53109797	Xp11.22	Probable G-protein coupled receptor 173
GPR30	1121844	1133451	7p22.3	G-protein coupled estrogen receptor 1
GPR4	46093022	46105466	19q13.32	G-protein coupled receptor 4
GPR41	35849362	35851387	19q13.12	Free fatty acid receptor 3
GPR55	231772033	231825781	2q37.1	G-protein coupled receptor 55
GPR84	54756229	54758271	12q13.13	G-protein coupled receptor 84
GPR92	6728001	6745613	12p13.31	Lysophosphatidic acid receptor 5
GPRC5C	72420990	72447792	17q25.1	G-protein coupled receptor family C group 5 member C
GPSM2	109417972	109477167	1p13.3	G-protein-signaling modulator 2
GPSN2	14627897	14676792	19p13.12	Very-long-chain enoyl-CoA reductase
GRAP2	40297086	40369725	22q13.1	GRB2-related adapter protein 2
GRB2	73314157	73401790	17q25.1	Growth factor receptor-bound protein 2
GRINL1A	57884231	58074960	15q21.3	DNA-directed RNA polymerase II subunit GRINL1A
GRO1	74735110	74736959	4q13.3	Growth-regulated alpha protein
GRP	56887400	56898006	18q21.32	Gastrin-releasing peptide
GRPEL1	7060633	7069924	4p16.1	GrpE protein homolog 1, mitochondrial
GRWD1	48949030	48960279	19q13.33	Glutamate-rich WD repeat-containing protein 1
GSG1L	27798850	28074830	16p12.1	Germ cell-specific gene 1-like protein
GSK3B	119540170	119813264	3q13.33	Glycogen synthase kinase-3 beta
GSS	33516236	33543620	20q11.22	Glutathione synthetase
GSTA3	52761437	52774483	6p12.2	Glutathione S-transferase A3
GSTP1	67351066	67354131	11q13.2	Glutathione S-transferase P
GSTT1	24376133	24384680	22q11.23	Glutathione S-transferase theta-1
GTF2A2	59930261	59949740	15q22.2	Transcription initiation factor IIA subunit 2
GTF2H1	18343842	18388591	11p15.1	General transcription factor IIH subunit 1
GTF2H4	30875961	30881883	6p21.33	General transcription factor IIH subunit 4
GTF2IRD1	73868120	74016931	7q11.23	General transcription factor II-I repeat domain-containing protein 1
GTSE1	46692638	46726707	22q13.31	G2 and S phase-expressed protein 1
GUK1	228327663	228336685	1q42.13	Guanylate kinase
GYLTL1B	45943172	45950647	11p11.2	Glycosyltransferase-like protein LARGE2
GZMA	54398476	54406080	5q11.2	Granzyme A
H2AFV	44866390	44887682	7p13	Histone H2A.V
H2AFZ	100869243	100871545	4q23	Histone H2A.Z
H3F3A	226249552	226259702	1q42.12	Histone H3.3
HAAO	42994229	43019733	2p21	3-hydroxyanthranilate 3,4-dioxygenase
HACL1	15602211	15643338	3p25.1	2-hydroxyacyl-CoA lyase 1
HADHA	26413504	26467594	2p23.3	Trifunctional enzyme subunit alpha, mitochondrial
HADHB	26466038	26513336	2p23.3	Trifunctional enzyme subunit beta, mitochondrial
HADHSC	108910870	108956331	4q25	Hydroxyacyl-coenzyme A dehydrogenase, mitochondrial
HAGH	1845621	1877195	16p13.3	Hydroxyacylglutathione hydrolase, mitochondrial
HAO2	119911402	119936753	1p12	Hydroxyacid oxidase 2
HAS1	52216365	52227247	19q13.41	Hyaluronan synthase 1
HAVCR1	156456424	156486130	5q33.3	Hepatitis A virus cellular receptor 1
HBA2	222846	223709	16p13.3	Hemoglobin subunit alpha
HBB	5246694	5250625	11p15.4	Hemoglobin subunit beta
HBE1	5289582	5526847	11p15.4	Hemoglobin subunit epsilon
HBG1	5269313	5271122	11p15.4	Hemoglobin subunit gamma-1
HDAC1	32757687	32799236	1p35.1	Histone deacetylase 1
HDAC3	141000443	141016437	5q31.3	Histone deacetylase 3
HDGF	156711899	156736717	1q23.1	Hepatoma-derived growth factor
HES4	934342	935552	1p36.33	Transcription factor HES-4

HEXIM1	43224684	43229468	17q21.31	Protein HEXIM1
HGS	79650356	79670168	17q25.3	Hepatocyte growth factor-regulated tyrosine kinase substrate
HHLA2	108015376	108097132	3q13.13	HERV-H LTR-associating protein 2
HIBADH	27565061	27702614	7p15.2	3-hydroxyisobutyrate dehydrogenase, mitochondrial
HIF1AN	102288829	102319755	10q24.31	Hypoxia-inducible factor 1-alpha inhibitor
HIGD2A	175815748	175816772	5q35.2	HIG1 domain family member 2A, mitochondrial
HIST1H2AC	26124373	26139344	6p22.2	Histone H2A type 1-C
HIST1H2AM	27860477	27860963	6p22.1	Histone H2A type 1
HIST1H3D	26197068	26199521	6p22.2	Histone H3.1
HIST1H4F	26240561	26240976	6p22.2	Histone H4
HIST2H2AA	149822643	149823191	1q21.2	Histone H2A type 2-A
HIST3H3	228612546	228613026	1q42.13	Histone H3.1t
HK1	71029740	71161638	10q22.1	Hexokinase-1
HK2	75061108	75120486	2p12	Hexokinase-2
HLA-C	31226380	31229751	6p21.33	HLA class I histocompatibility antigen, Cw-4 alpha chain
HLA-DMB	32831136	32849631	6p21.32	HLA class II histocompatibility antigen, DM beta chain
HLA-DOB	32780540	32784825	6p21.32	HLA class II histocompatibility antigen, DO beta chain
HLA-DPA1	33032346	33048552	6p21.32	HLA class II histocompatibility antigen, DP alpha 1 chain
HLA-DQA1	32533367	32539589	6p21.32	HLA class II histocompatibility antigen, DQ alpha 1 chain
HLA-DQB1	32658022	32667070	6p21.32	HLA class II histocompatibility antigen, DQ beta 1 chain
HLA-DRA	32365090	32370300	6p21.32	HLA class II histocompatibility antigen, DR alpha chain
HLA-E	30446762	30451500	6p21.33	HLA class I histocompatibility antigen, alpha chain E
HLX1	221051699	221058401	1q41	H2.0-like homeobox protein
HM13	30102231	30157370	20q11.21	Minor histocompatibility antigen H13
HMGB2	174252846	174256276	4q34.1	High mobility group protein B2
HMGIY	34204650	34214008	6p21.31	High mobility group protein HMG-I/HMG-Y
HMOX1	35776354	35790207	22q12.3	Heme oxygenase 1
HMOX2	4524691	4560348	16p13.3	Heme oxygenase 2
HN1	73131343	73164376	17q25.1	Hematological and neurological expressed 1 protein
HNF4G	76320149	76479078	8q21.11	Hepatocyte nuclear factor 4-gamma
HNMT	138721590	138773930	2q22.1	Histamine N-methyltransferase
HOMER2	83509838	83654661	15q25.2	Homer protein homolog 2
HOMER3	19040010	19052070	19p13.11	Homer protein homolog 3
HOOK1	60280458	60342050	1p32.1	Protein Hook homolog 1
HOXA10	27210210	27219880	7p15.2	Homeobox protein Hox-A10
HOXA5	27180671	27183287	7p15.2	Homeobox protein Hox-A5
HOXA9	27202054	27210117	7p15.2	Homeobox protein Hox-A9
HOXB13	46802125	46806540	17q21.32	Homeobox protein Hox-B13
HOXB7	46684594	46710934	17q21.32	Homeobox protein Hox-B7
HOXD3	177001340	177037830	2q31.1	Homeobox protein Hox-D3
HPD	122277433	122301502	12q24.31	4-hydroxyphenylpyruvate dioxygenase
HPRT1	133594183	133654543	Xq26.2	Hypoxanthine-guanine phosphoribosyltransferase
HRAS	532242	537287	11p15.5	GTPase HRas
HRASLS3	63340667	63384355	11q12.3	HRAS-like suppressor 3
HRSP12	99114572	99129469	8q22.2	Ribonuclease UK114

HRY	193853934	193856521	3q29	Transcription factor HES-1
HSA9761	61683081	61699766	5q12.1	Probable dimethyladenosine transferase
HSD17B3	98997588	99064434	9q22.32	Testosterone 17-beta-dehydrogenase 3
HSD3B2	119957554	119965658	1p12	3 beta-hydroxysteroid dehydrogenase/Delta 5-->4-isomerase type 2
HSP90AB1	44214824	44221620	6p21.1	Heat shock protein HSP 90-beta
HSPA2	65002623	65009955	14q23.3	Heat shock-related 70 kDa protein 2
HSPA6	161494036	161496681	1q23.3	Heat shock 70 kDa protein 6
HSPA8	122928197	122933938	11q24.1	Heat shock cognate 71 kDa protein
HSPB7	16340523	16346089	1p36.13	Heat shock protein beta-7
HSPBAP1	122458846	122512671	3q21.1	HSPB1-associated protein 1
HSPBP1	55773599	55791749	19q13.42	Hsp70-binding protein 1
HSPC023	13884982	13889276	19p13.2	Leydig cell tumor 10 kDa protein homolog
HSPC047	102,672,879	102,678,856	7q22.1	-
HSPC152	64083932	64085556	11q13.1	Multifunctional methyltransferase subunit TRM112-like protein
HSPC171	67261006	67263181	16q22.1	Transmembrane protein 208
HSPC268	139024203	139031065	7q34	UPF0562 protein C7orf55
HSPD1	198351305	198381461	2q33.1	60 kDa heat shock protein, mitochondrial
HSPE1	198364718	198368181	2q33.1	10 kDa heat shock protein, mitochondrial
HTR3A	113845603	113861035	11q23.2	5-hydroxytryptamine receptor 3A
HTRA3	8271492	8308838	4p16.1	Serine protease HTRA3
HUMMAT1H	160175127	160185166	1q23.2	Astrocytic phosphoprotein PEA-15
HYPE	11200038	11244492	19p13.2	Low-density lipoprotein receptor
IBSP	88720733	88733074	4q22.1	Bone sialoprotein 2
ICAM2	62079954	62097994	17q23.3	Intercellular adhesion molecule 2
ICAM4	10397643	10399198	19p13.2	Intercellular adhesion molecule 4
ICMT	6281253	6296032	1p36.31	Protein-S-isoprenylcysteine O-methyltransferase
ID1	30193086	30194318	20q11.21	DNA-binding protein inhibitor ID-1
ID2	8818975	8824583	2p25.1	DNA-binding protein inhibitor ID-2
ID3	23884409	23886285	1p36.12	DNA-binding protein inhibitor ID-3
IDH3A	78423840	78464291	15q25.1	Isocitrate dehydrogenase [NAD] subunit alpha, mitochondrial
IDH3B	2639041	2644865	20p13	Isocitrate dehydrogenase [NAD] subunit beta, mitochondrial
IER2	13261229	13265722	19p13.2	Immediate early response gene 2 protein
IFI27	94571182	94583033	14q32.12	Interferon alpha-inducible protein 27, mitochondrial
IFI30	18283972	18288927	19p13.11	Gamma-interferon-inducible lysosomal thiol reductase
IFI35	41158742	41166473	17q21.31	Interferon-induced 35 kDa protein
IFITM1	313506	315272	11p15.5	Interferon-induced transmembrane protein 1
IFITM2	307631	315272	11p15.5	Interferon-induced transmembrane protein 2
IFNA17	21227242	21228221	9p21.3	Interferon alpha-17
IFNA2	21384254	21385396	9p21.3	Interferon alpha-2
IFNA21	21165636	21166659	9p21.3	Interferon alpha-21
IFNAR2	34602206	34637980	21q22.11	Interferon alpha/beta receptor 2
IFT57	107879659	107941417	3q13.12	Intraflagellar transport protein 57 homolog
IGBP1	69353299	69386174	Xq13.1	Immunoglobulin-binding protein 1
IGF1	102789645	102874423	12q23.2	Insulin-like growth factor I
IGF2	2150342	2170833	11p15.5	Insulin-like growth factor II
IGFALS	1840414	1844972	16p13.3	Insulin-like growth factor-binding protein complex acid labile subunit
IGFBP1	45927956	45933267	7p12.3	Insulin-like growth factor-binding protein 1
IGFBP3	45951949	45961473	7p12.3	Insulin-like growth factor-binding protein 3
IGFBP4	38599702	38613983	17q21.2	Insulin-like growth factor-binding protein 4

IGFBP5	217536828	217560248	2q35	Insulin-like growth factor-binding protein 5
IGFBP6	53491220	53496129	12q13.13	Insulin-like growth factor-binding protein 6
IGFBP7	57896939	57976551	4q12	Insulin-like growth factor-binding protein 7
IGL@	22,026,076	22,922,913	22q11.2	Immunoglobulin Lambda Locus
IGLL1	23915312	23922495	22q11.23	Immunoglobulin lambda-like polypeptide 1
IGSF9	159896829	159915386	1q23.2	Protein turtle homolog A
IHPK2	48725436	48777786	3p21.31	Inositol hexakisphosphate kinase 2
IKBKG	153769414	153796782	Xq28	NF-kappa-B essential modulator
IL10	206940947	206945839	1q32.1	Interleukin-10
IL10RB	34638663	34669539	21q22.11	Interleukin-10 receptor subunit beta
IL11	55875757	55881831	19q13.42	Interleukin-11
IL11RA	34650699	34661889	9p13.3	Interleukin-11 receptor subunit alpha
IL13	131991955	131996802	5q31.1	Interleukin-13
IL13RA1	117861535	117928502	Xq24	Interleukin-13 receptor subunit alpha-1
IL15	142557752	142655140	4q31.21	Interleukin-15
IL17RA	17565844	17596583	22q11.1	Interleukin-17 receptor A
IL18	112013974	112034840	11q23.1	Interleukin-18
IL1A	113531492	113542167	2q13	Interleukin-1 alpha
IL1B	113587328	113594480	2q13	Interleukin-1 beta
IL1RN	113864791	113891593	2q13	Interleukin-1 receptor antagonist protein
IL21R	27413483	27462115	16p12.1	Interleukin-21 receptor
IL3RA	1455509	1501578	Xp22.33	Interleukin-3 receptor subunit alpha
IL4	132009678	132018368	5q31.1	Interleukin-4
IL6	22765503	22771621	7p15.3	Interleukin-6
IL6R	154377669	154441926	1q21.3	Interleukin-6 receptor subunit alpha
IL7	79587978	79717758	8q21.12	Interleukin-7
IL8	219027568	219031718	2q35	C-X-C chemokine receptor type 1
IL8RA	218,162,845	218,166,995	2q35	Chemokine (C-X-C Motif) Receptor 1
IL8RB	218990012	219001976	2q35	C-X-C chemokine receptor type 2
IL9	135227935	135231516	5q31.1	Interleukin-9
ILF2	153634512	153643524	1q21.3	Interleukin enhancer-binding factor 2
ILK	6624961	6632102	11p15.4	Integrin-linked protein kinase
ILKAP	239079042	239112370	2q37.3	Integrin-linked kinase-associated serine/threonine phosphatase 2C
IMP4	131099798	131105383	2q21.1	U3 small nucleolar ribonucleoprotein protein IMP4
IMPDH2	49061758	49066841	3p21.31	Inosine-5'-monophosphate dehydrogenase 2
INA	105036920	105050108	10q24.33	Alpha-internexin
INHBA	41724712	41742706	7p14.1	Inhibin beta A chain
INS	2181009	2182571	11p15.5	Insulin
INSL5	67263424	67266939	1p31.3	Insulin-like peptide INSL5
IRF1	131817301	131826490	5q31.1	Interferon regulatory factor 1
IRF3	50162826	50169132	19q13.33	Interferon regulatory factor 3
IRF4	391739	411447	6p25.3	Interferon regulatory factor 4
IRF5	128577666	128590089	7q32.1	Interferon regulatory factor 5
ISL1	50678921	50690564	5q11.1	Insulin gene enhancer protein ISL-1
ISYNA1	18545198	18549111	19p13.11	Inositol-3-phosphate synthase 1
ITFG1	47188298	47498060	16q12.1	T-cell immunomodulatory protein
ITFG2	2921788	2968957	12p13.33	Integrin-alpha FG-GAP repeat-containing protein 2
ITGAX	31366455	31394318	16p11.2	Integrin alpha-X
ITGB1	33189247	33294720	10p11.22	Integrin beta-1
ITGB2	46305868	46351904	21q22.3	Integrin beta-2
ITGB4BP	33866714	33872788	20q11.22	Eukaryotic translation initiation factor 6
ITGB5	124480795	124620265	3q21.2	Integrin beta-5
ITPK1	93403259	93582665	14q32.12	Inositol-tetrakisphosphate 1-kinase
IVD	40697686	40728146	15q15.1	Isovaleryl-CoA dehydrogenase, mitochondrial

JAGN1	9932238	9936033	3p25.3	Protein jagunal homolog 1
JPH3	87635441	87731762	16q24.2	Junctophilin-3
JUB	23440383	23451851	14q11.2	LIM domain-containing protein ajuba
JUN	59246465	59249785	1p32.1	Transcription factor AP-1
JUNB	12902310	12904124	19p13.2	Transcription factor jun-B
JUP	39775692	39943183	17q21.2	Junction plakoglobin
KARS	75661622	75682541	16q23.1	Lysine--tRNA ligase
KATNAL1	30776767	30881621	13q12.3	Katanin p60 ATPase-containing subunit A-like 1
KAZALD1	102821598	102827888	10q24.31	Kazal-type serine protease inhibitor domain-containing protein 1
KBTBD10	170366212	170382772	2q31.1	Kelch-like protein 41
KCNAB2	6051526	6161253	1p36.31	Voltage-gated potassium channel subunit beta-2
KCNE1	35818988	35884573	21q22.12	Potassium voltage-gated channel subfamily E member 1
KCNE2	35736323	35743688	21q22.11	Potassium voltage-gated channel subfamily E member 2
KCNG4	84255823	84273356	16q24.1	Potassium voltage-gated channel subfamily G member 4
KCNJ15	39529128	39679279	21q22.13	ATP-sensitive inward rectifier potassium channel 15
KCNJ8	21917889	21928515	12p12.1	ATP-sensitive inward rectifier potassium channel 8
KCNK6	38810484	38819660	19q13.2	Potassium channel subfamily K member 6
KCNN4	44270685	44285409	19q13.31	Intermediate conductance calcium-activated potassium channel protein 4
KCNRG	50589390	50595058	13q14.2	Potassium channel regulatory protein
KCNS3	18059114	18542882	2p24.2	Potassium voltage-gated channel subfamily S member 3
KCTD13	29916333	29938356	16p11.2	BTB/POZ domain-containing adapter for CUL3-mediated RhoA degradation protein 1
KCTD14	77726761	77757237	11q14.1	BTB/POZ domain-containing protein KCTD14
KDELC1	103436631	103451357	13q33.1	KDEL motif-containing protein 1
KDELR1	48885827	48894810	19q13.33	ER lumen protein-retaining receptor 1
KDELR3	38864067	38879452	22q13.1	ER lumen protein-retaining receptor 3
KEL	142638201	142659768	7q34	Kell blood group glycoprotein
KIF22	29802040	29816706	16p11.2	Kinesin-like protein KIF22
KIF9	47269516	47324941	3p21.31	Kinesin-like protein KIF9
KISS1	204159469	204165614	1q32.1	Metastasis-suppressor KiSS-1
KLC2	66024765	66035331	11q13.2	Kinesin light chain 2
KLF12	74260226	74708394	13q22.1	Krueppel-like factor 12
KLF4	110247133	110252763	9q31.2	Krueppel-like factor 4
KLHL6	183205319	183273477	3q27.1	Kelch-like protein 6
KLK1	51322404	51327043	19q13.33	Kallikrein-1
KLK3	51358171	51364020	19q13.33	Prostate-specific antigen
KLRC1	10594863	10607284	12p13.2	NKG2-A/NKG2-B type II integral membrane protein
KLRC4	10559983	10562356	12p13.2	NKG2-F type II integral membrane protein
KLRG1	9102640	9163356	12p13.31	Killer cell lectin-like receptor subfamily G member 1
KNS2	104028233	104167888	14q32.33	Kinesin light chain 1
KPNA1	122140796	122233792	3q21.1	Importin subunit alpha-5
KPNA3	50273447	50367057	13q14.2	Importin subunit alpha-4
KPNA6	32573639	32642169	1p35.1	Importin subunit alpha-7
KREMEN2	3013945	3018384	16p13.3	Kremen protein 2
KRT15	39669995	39678781	17q21.2	Keratin, type I cytoskeletal 15
KRT17	39775689	39781094	17q21.2	Keratin, type I cytoskeletal 17
KRT19	39679869	39684560	17q21.2	Keratin, type I cytoskeletal 19
KRT33B	39519746	39526052	17q21.2	Keratin, type I cuticular Ha3-II

KRT5	52908359	52914471	12q13.13	Keratin, type II cytoskeletal 5
KRT7	52626304	52645970	12q13.13	Keratin, type II cytoskeletal 7
KRT8	53290977	53343738	12q13.13	Keratin, type II cytoskeletal 8
KRTAP4-12	39279343	39280419	17q21.2	Keratin-associated protein 4-12
L3MBTL2	41601209	41627275	22q13.2	Lethal(3)malignant brain tumor-like protein 2
LAD1	201342372	201368736	1q32.1	Ladinin-1
LAIR1	54865362	54882165	19q13.42	Leukocyte-associated immunoglobulin-like receptor 1
LALBA	48961467	48963849	12q13.11	Alpha-lactalbumin
LAMP1	113951556	113977987	13q34	Lysosome-associated membrane glycoprotein 1
LAMP2	119561682	119603220	Xq24	Lysosome-associated membrane glycoprotein 2
LANCL1	211295973	211342376	2q34	LanC-like protein 1
LASP1	37026112	37078023	17q12	LIM and SH3 domain protein 1
LASS4	8271620	8327305	19p13.2	Ceramide synthase 4
LBP	36974759	37005665	20q11.23	Lipopolysaccharide-binding protein
LCN2	130911350	130915734	9q34.11	Neutrophil gelatinase-associated lipocalin
LDB2	16503164	16900432	4p15.32	LIM domain-binding protein 2
LDHA	18415935	18429972	11p15.1	L-lactate dehydrogenase A chain
LDHB	21788276	21910791	12p12.1	L-lactate dehydrogenase B chain
LECT1	53277399	53313947	13q14.3	Leukocyte cell-derived chemotaxin 1
LEPROTL1	29952914	30034724	8p12	Leptin receptor overlapping transcript-like 1
LGALS1	38071615	38075813	22q13.1	Galectin-1
LGALS3	55590828	55612126	14q22.3	Galectin-3
LGALS8	236681300	236716281	1q43	Galectin-8
LGR4	27387508	27494322	11p14.1	Leucine-rich repeat-containing G-protein coupled receptor 4
LGTN	206744620	206785904	1q32.1	Eukaryotic translation initiation factor 2D
LHFP	39917029	40177665	13q13.3	Lipoma HMGIC fusion partner
LHFPL5	35773070	35801651	6p21.31	Tetraspan membrane protein of hair cell stereocilia
LHX4	180199421	180249380	1q25.2	LIM/homeobox protein Lhx4
LIG4	108859787	108870716	13q33.3	DNA ligase 4
LIN28	26,410,778	26,429,728	1p36.11	Lin-28 Homolog A
LMAN1	56995055	57027194	18q21.32	Protein ERGIC-53
LMBR1L	49490919	49504683	12q13.12	Protein LMBR1L
LMCD1	8543393	8609805	3p26.1	LIM and cysteine-rich domains protein 1
LMO4	87794151	87814606	1p22.3	LIM domain transcription factor LMO4
LMO6	49031151	49042845	Xp11.23	Prickle-like protein 3
LOC200420	73872046	73912703	2p13.1	Putative ALMS1-like protein
LOH12CR1	12510013	12619840	12p13.2	Loss of heterozygosity 12 chromosomal region 1 protein
LOXL2	23154702	23282841	8p21.3	Lysyl oxidase homolog 2
LPIN1	11817721	11967535	2p25.1	Phosphatidate phosphatase LPIN1
LPXN	58294344	58345693	11q12.1	Leupaxin
LRCH3	197518097	197615307	3q29	Leucine-rich repeat and calponin homology domain-containing protein 3
LRFN3	36426260	36436669	19q13.12	Leucine-rich repeat and fibronectin type-III domain-containing protein 3
LRP12	105501459	105601252	8q22.3	Low-density lipoprotein receptor-related protein 12
LRRC29	67241042	67260951	16q22.1	Leucine-rich repeat-containing protein 29
LSM2	31765173	31774761	6p21.33	U6 snRNA-associated Sm-like protein LSm2
LSM7	2321516	2328619	19p13.3	U6 snRNA-associated Sm-like protein LSm7
LSS	47608055	47648738	21q22.3	Lanosterol synthase
LTA	31539831	31542101	6p21.33	Lymphotoxin-alpha
LUM	91496406	91505608	12q21.33	Lumican
LY6H	144239331	144242128	8q24.3	Lymphocyte antigen 6H

LYPLA1	54958938	55014577	8q11.23	Acyl-protein thioesterase 1
LYPLA2	24117460	24122029	1p36.11	Acyl-protein thioesterase 2
LYSMD2	52015208	52043782	15q21.2	LysM and putative peptidoglycan-binding domain-containing protein 2
LYZ	69742121	69748014	12q15	Lysozyme C
LZTR1	21333751	21353327	22q11.21	Leucine-zipper-like transcriptional regulator 1
M6PRBP1	4838353	4867780	19p13.3	Perilipin-3
MAD	35870717	35873955	14q13.2	NF-kappa-B inhibitor alpha
MAFK	1570350	1582679	7p22.3	Transcription factor MafK
Mage3	151934652	151938240	Xq28	Melanoma-associated antigen 3
Mage6	151867214	151870825	Xq28	Melanoma-associated antigen 6
MAGEA1	152481522	152486115	Xq28	Melanoma-associated antigen 1
MAGEA10	151301782	151307033	Xq28	Melanoma-associated antigen 10
MAGEA3	151934652	151938240	Xq28	Melanoma-associated antigen 3
MAGEA6	151867214	151870825	Xq28	Melanoma-associated antigen 6
MAGEA9	148863584	148869397	Xq28	Melanoma-associated antigen 9
MAGEB4	30260057	30262293	Xp21.2	Melanoma-associated antigen B4
MAGEB4	30260057	30262293	Xp21.2	Melanoma-associated antigen B4
MAGED2	54834032	54842445	Xp11.21	Melanoma-associated antigen D2
MAGEH1	55478538	55479998	Xp11.21	Melanoma-associated antigen H1
MALT1	56338618	56417371	18q21.32	Mucosa-associated lymphoid tissue lymphoma translocation protein 1
MAP1LC3B	87417601	87438385	16q24.2	Microtubule-associated proteins 1A/1B light chain 3B
MAP2K3	21187984	21218552	17p11.2	Dual specificity mitogen-activated protein kinase kinase 3
MAP2K6	67410839	67539472	17q24.3	Dual specificity mitogen-activated protein kinase kinase 6
MAP2K7	7968728	7979363	19p13.2	Dual specificity mitogen-activated protein kinase kinase 7
MAP3K8	30722866	30750762	10p11.23	Mitogen-activated protein kinase kinase kinase 8
MAPBPIP	156024543	156028301	1q22	Ragulator complex protein LAMTOR2
MAPK10	86936276	87515284	4q21.3	Mitogen-activated protein kinase 10
MAPK12	50683879	50700254	22q13.33	Mitogen-activated protein kinase 12
MAPK13	36095586	36107842	6p21.31	Mitogen-activated protein kinase 13
MAPK15	144798429	144804628	8q24.3	Mitogen-activated protein kinase 15
MAPK3	30125426	30134827	16p11.2	Mitogen-activated protein kinase 3
MAPK6	52244303	52358462	15q21.2	Mitogen-activated protein kinase 6
MAPK8	49514698	49647403	10q11.22	Mitogen-activated protein kinase 8
MAPRE3	27193480	27250064	2p23.3	Microtubule-associated protein RP/EB family member 3
MAPRE3	27193480	27250064	2p23.3	Microtubule-associated protein RP/EB family member 3
MARCKSL1	32799433	32801980	1p35.1	MARCKS-related protein
MATR3	138609441	138667360	5q31.2	Matrin-3
MAX	65472892	65569413	14q23.3	Protein max
MB	36002811	36033998	22q12.3	Myoglobin
MBP	74690783	74845639	18q23	Myelin basic protein
MC1R	89978527	89987385	16q24.3	Melanocyte-stimulating hormone receptor
MCEE	71336814	71357369	2p13.3	Methylmalonyl-CoA epimerase, mitochondrial
MCF2L	113548692	113754053	13q34	Guanine nucleotide exchange factor DBS
MCFD2	47129009	47168994	2p21	Multiple coagulation factor deficiency protein 2
MCM3	52128807	52149679	6p12.2	DNA replication licensing factor MCM3
MCM7	99690351	99699563	7q22.1	DNA replication licensing factor MCM7
MCMDC1	119134605	119256327	6q22.31	DNA helicase MCM9

MDH2	75677369	75696826	7q11.23	Malate dehydrogenase, mitochondrial
MDK	46402306	46405375	11p11.2	Midkine
MDS1	169,083,499	169,663,775	3q26.2	Myelodysplasia Syndrome-Associated Protein 1
MED28	17616254	17635728	4p15.32	Mediator of RNA polymerase II transcription subunit 28
MED4	48627459	48669267	13q14.2	Mediator of RNA polymerase II transcription subunit 4
MED8	43849588	43855479	1p34.2	Mediator of RNA polymerase II transcription subunit 8
MEST	130126012	130146133	7q32.2	Mesoderm-specific transcript homolog protein
METTL6	15422782	15482073	3p25.1	Methyltransferase-like protein 6
MFAP1	44096690	44117000	15q15.3	Microfibrillar-associated protein 1
MFAP3	153418466	153600038	5q33.2	Microfibril-associated glycoprotein 3
MGAT1	180217541	180242652	5q35.3	Alpha-1,3-mannosyl-glycoprotein 2-beta-N-acetylglucosaminyltransferase
MGAT2	50087489	50090198	14q21.3	Alpha-1,6-mannosyl-glycoprotein 2-beta-N-acetylglucosaminyltransferase
MGC16169	106962756	107242652	4q24	TBC domain-containing protein kinase-like protein
MGC18216	98,648,971	98,964,530	15q26.3	Insulin-Like Growth Factor 1 Receptor
MGC26597	7,986,102	7,990,344	6p24.3	Phosphatidylinositol-4-Phosphate 5-Kinase, Type I, Pseudogene 1
MGC39372	2,854,657	2,881,407	6p25.2	erpin Peptidase Inhibitor, Clade B (Ovalbumin), Member 9, Pseudogene 1
MGC39518	201838441	201936394	2q33.1	Protein FAM126B
MGC40574	43,227,211	43,228,855	2p21	Long Intergenic Non-Protein Coding RNA 1126
MGC40579	141882414	141944449	3q23	Putative glycerol kinase 5
MGC5509	105953816	105965668	2q12.1	Ashwin
MGC7036	123899936	123921264	12q24.31	RILP-like protein 2
MGLL	127407909	127542051	3q21.3	Monoglyceride lipase
MGP	15034115	15038860	12p12.3	Matrix Gla protein
MGST2	140586922	140661899	4q31.1	Microsomal glutathione S-transferase 2
MIA	41277553	41283395	19q13.2	Melanoma-derived growth regulatory protein
MIF	24236191	24237414	22q11.23	Macrophage migration inhibitory factor
MINK1	4736683	4801356	17p13.2	Misshapen-like kinase 1
MIPEP	24304328	24463558	13q12.12	Mitochondrial intermediate peptidase
MIZF	118992297	119006752	11q23.3	Histone H4 transcription factor
MKNK1	47023090	47082515	1p33	MAP kinase-interacting serine/threonine-protein kinase 1
MKRN2	12598513	12625212	3p25.2	Probable E3 ubiquitin-protein ligase makorin-2
MLANA	5890802	5910606	9p24.1	Melanoma antigen recognized by T-cells 1
MLF2	6857170	6876641	12p13.31	Myeloid leukemia factor 2
MLH1	37034823	37107380	3p22.2	DNA mismatch repair protein Mlh1
ML-IAP	61867235	61871859	20q13.33	Baculoviral IAP repeat-containing protein 7
MLLT11	151030234	151040970	1q21.3	Protein AF1q
MMP1	102660651	102668891	11q22.2	Interstitial collagenase
MMP10	102641234	102651359	11q22.2	Stromelysin-2
MMP2	55423612	55540603	16q12.2	72 kDa type IV collagenase
MMP9	44637547	44645200	20q13.12	Matrix metalloproteinase-9
MOBKL2B	27325207	27529779	9p21.2	MOB kinase activator 3B
MOCOS	33767482	33852120	18q12.2	Molybdenum cofactor sulfurase
MORF4L1	79102829	79190475	15q25.1	Mortality factor 4-like protein 1
MOSPD1	134021656	134049297	Xq26.3	Motile sperm domain-containing protein 1
MPB1	8921061	8939308	1p36.23	Alpha-enolase
MPG	127006	135852	16p13.3	DNA-3-methyladenine glycosylase
MPP1	154006959	154049282	Xq28	55 kDa erythrocyte membrane protein

MPP6	24612887	24733812	7p15.3	MAGUK p55 subfamily member 6
MPST	37415676	37425863	22q12.3	3-mercaptopyruvate sulfurtransferase
MPV17	27532360	27548547	2p23.3	Protein Mpv17
MPZ	161274525	161279762	1q23.3	Myelin protein P0
MRAS	138066539	138124375	3q22.3	Ras-related protein M-Ras
MRCL3	3247479	3256234	18p11.31	Myosin regulatory light chain 12A
MRFAP1	6641818	6644472	4p16.1	MORF4 family-associated protein 1
MRP63	21750784	21753223	13q12.11	Ribosomal protein 63, mitochondrial
MRPL10	45900638	45908900	17q21.32	39S ribosomal protein L10, mitochondrial
MRPL17	6702013	6704632	11p15.4	39S ribosomal protein L17, mitochondrial
MRPL19	75873909	75917977	2p12	39S ribosomal protein L19, mitochondrial
MRPL40	19419425	19423598	22q11.21	39S ribosomal protein L40, mitochondrial
MRPS11	89010684	89022222	15q25.3	28S ribosomal protein S11, mitochondrial
MRPS5	95752952	95815179	2q11.1	28S ribosomal protein S5, mitochondrial
MS4A1	60223225	60238233	11q12.2	B-lymphocyte antigen CD20
MS4A4A	60048014	60076445	11q12.2	Membrane-spanning 4-domains subfamily A member 4A
MS4A6A	59939081	59952139	11q12.2	Membrane-spanning 4-domains subfamily A member 6A
MS4A7	60145955	60163424	11q12.2	Membrane-spanning 4-domains subfamily A member 7
MSH2	47630108	47789450	2p21	DNA mismatch repair protein Msh2
MSH4	76262567	76378923	1p31.1	MutS protein homolog 4
MSI2	55333212	55762046	17q22	RNA-binding protein Musashi homolog 2
MSMB	51549498	51562517	10q11.23	Beta-microseminoprotein
MSN	64808257	64961791	Xq12	Moesin
MSX2	174151536	174157896	5q35.2	Homeobox protein MSX-2
MT1A	56672578	56673999	16q12.2	Metallothionein-1A
MT1M	56666145	56667898	16q12.2	Metallothionein-1M
MT3	56622986	56625000	16q12.2	Metallothionein-3
MTAP	21802542	21931646	9p21.3	S-methyl-5'-thioadenosine phosphorylase
MTCH2	47638867	47664175	11p11.2	Mitochondrial carrier homolog 2
MTHFD1	64854749	64926722	14q23.3	C-1-tetrahydrofolate synthase, cytoplasmic
MUC1	155158300	155162707	1q22	Mucin-1
MVK	110011060	110035067	12q24.11	Mevalonate kinase
MVP	29831715	29859355	16p11.2	Major vault protein
MX1	42792231	42831141	21q22.3	Interferon-induced GTP-binding protein Mx1
MXD3	176728462	176739758	5q35.3	Max dimerization protein 3
MYB	135502453	135540311	6q23.3	Transcriptional activator Myb
MYBL2	42295754	42345136	20q13.12	Myb-related protein B
MYC	128747680	128753674	8q24.21	Myc proto-oncogene protein
MYCBP	39328636	39347289	1p34.3	C-Myc-binding protein
MYF6	81101277	81103253	12q21.31	Myogenic factor 6
MYL2	111348623	111358526	12q24.11	Myosin regulatory light chain 2, ventricular/cardiac muscle isoform
MYNN	169490619	169507504	3q26.2	Myoneurin
MYOC	171604557	171621823	1q24.3	Myocilin
MYOZ1	75391412	75401515	10q22.2	Myozenin-1
NAGK	71291474	71306935	2p13.3	N-acetyl-D-glucosamine kinase
NAPSA	50861734	50869087	19q13.33	Napsin-A
NASP	46049518	46084566	1p34.1	Nuclear autoantigenic sperm protein
NAT13	113435307	113465147	3q13.2	N-alpha-acetyltransferase 50
NAT5	19997760	20014299	20p11.23	N-alpha-acetyltransferase 20
NBL1	19967048	19984945	1p36.13	Neuroblastoma suppressor of tumorigenicity 1
NCDN	36023074	36032875	1p34.3	Neurochondrin

NCF1	74188309	74203659	7q11.23	Neutrophil cytosol factor 1
NCF2	183524698	183560011	1q25.3	Neutrophil cytosol factor 2
NCOA4	51565108	51590734	10q11.23	Nuclear receptor coactivator 4
NDUFA3	54606036	54612564	19q13.42	NADH dehydrogenase [ubiquinone] 1 alpha sub-complex subunit 3
NDUFA6	42481529	42486959	22q13.2	NADH dehydrogenase [ubiquinone] 1 alpha sub-complex subunit 6
NDUFB3	201936156	201950473	2q33.1	NADH dehydrogenase [ubiquinone] 1 beta subcomplex subunit 3
NDUFB4	120315156	120321347	3q13.33	NADH dehydrogenase [ubiquinone] 1 beta subcomplex subunit 4
NDUFB7	14676890	14682874	19p13.12	NADH dehydrogenase [ubiquinone] 1 beta subcomplex subunit 7
NDUFS3	47586888	47606114	11p11.2	NADH dehydrogenase [ubiquinone] iron-sulfur protein 3, mitochondrial
NDUFV1	67374323	67380006	11q13.2	NADH dehydrogenase [ubiquinone] flavoprotein 1, mitochondrial
NDUFV2	9102628	9134343	18p11.22	NADH dehydrogenase [ubiquinone] flavoprotein 2, mitochondrial
NDUFV3	44299754	44333414	21q22.3	NADH dehydrogenase [ubiquinone] flavoprotein 3, mitochondrial
NEDD1	97301001	97347129	12q23.1	Protein NEDD1
NEFL	24808468	24814624	8p21.2	Neurofilament light polypeptide
NEIL1	75639296	75647592	15q24.2	Endonuclease 8-like 1
NEUROD1	182537815	182545603	2q31.3	Neurogenic differentiation factor 1
NEUROG1	134869991	134871639	5q31.1	Neurogenin-1
NF2	29999545	30094587	22q12.2	Merlin
NFE2	54685895	54694905	12q13.13	Transcription factor NF-E2 45 kDa subunit
NFIA	61330931	61928465	1p31.3	Nuclear factor 1 A-type
NFIB	14081842	14398982	9p22.3	Nuclear factor 1 B-type
NFIC	3359561	3469215	19p13.3	Nuclear factor 1 C-type
NFKBIA	35870717	35873955	14q13.2	NF-kappa-B inhibitor alpha
NFKBIL2	145654165	145669827	8q24.3	Tonsoku-like protein
NFX1	33290509	33371155	9p13.3	Transcriptional repressor NF-X1
NFYB	104510855	104532067	12q23.3	Nuclear transcription factor Y subunit beta
NGFB	115828539	115880857	1p13.2	Beta-nerve growth factor
NGLY1	25760435	25831530	3p24.2	Peptide-N(4)-(N-acetyl-beta-glucosaminyl)asparagine amidase
NIF3L1	201754050	201768655	2q33.1	NIF3-like protein 1
NIFUN	108956358	108963160	12q23.3	Iron-sulfur cluster assembly enzyme ISCU, mitochondrial
NIP30	57186378	57220028	16q13	Protein FAM192A
NIP7	69373333	69377014	16q22.1	60S ribosome subunit biogenesis protein NIP7 homolog
NIPA1	23043277	23100005	15q11.2	Magnesium transporter NIPA1
NIT1	161087876	161095235	1q23.3	Nitrilase homolog 1
NKG7	51874860	51875969	19q13.41	Protein NKG7
NKIRAS2	40163400	40177659	17q21.2	NF-kappa-B inhibitor-interacting Ras-like protein 2
NME6	48334754	48343175	3p21.31	Nucleoside diphosphate kinase 6
NMUR2	151771093	151812929	5q33.1	Neuromedin-U receptor 2
NOL3	67204057	67209643	16q22.1	Nucleolar protein 3
NOV	120428546	120436593	8q24.12	Protein NOV homolog
NPC2	74942895	74960880	14q24.3	Epididymal secretory protein E1
NPEPL1	57264187	57294294	20q13.32	Probable aminopeptidase NPEPL1
NPM1	170814120	170838141	5q35.1	Nucleophosmin

NPPB	11917521	11918988	1p36.22	Natriuretic peptides B
NPTX2	98246609	98259180	7q22.1	Neuronal pentraxin-2
NPY1R	164245113	164265984	4q32.2	Neuropeptide Y receptor type 1
NR1D2	23986751	24022109	3p24.2	Nuclear receptor subfamily 1 group D member 2
NR1H3	47269851	47290396	11p11.2	Oxysterols receptor LXR-alpha
NR2E1	108487262	108510013	6q21	Nuclear receptor subfamily 2 group E member 1
NRBP1	27650657	27665126	2p23.3	Nuclear receptor-binding protein
NRIP1	16333556	16437321	21q11.2	Nuclear receptor-interacting protein 1
NRSN1	24126350	24155128	6p22.3	Neurensin-1
NSDHL	151999511	152038273	Xq28	Sterol-4-alpha-carboxylate 3-dehydrogenase, decarboxylating
NSFL1C	1422807	1454487	20p13	NSFL1 cofactor p47
NSUN5	72716514	72722864	7q11.23	Probable 28S rRNA (cytosine-C(5))-methyltransferase
NT5C2	104845940	104953056	10q24.32	Cytosolic purine 5'-nucleotidase
NUCKS1	205681947	205719404	1q32.1	Nuclear ubiquitous casein and cyclin-dependent kinase substrate 1
NUDT1	2281857	2290781	7p22.3	7,8-dihydro-8-oxoguanine triphosphatase
NUDT2	34329504	34343709	9p13.3	Bis(5'-nucleosyl)-tetraphosphatase [asymmetrical]
NUDT3	34247456	34360451	6p21.31	Diphosphoinositol polyphosphate phosphohydrolase 1
NUDT4	93771659	93797024	12q22	Diphosphoinositol polyphosphate phosphohydrolase 2
NUDT5	12207324	12238143	10p13	ADP-sugar pyrophosphatase
NUDT6	123809852	123844123	4q28.1	Nucleoside diphosphate-linked moiety X motif 6
NUMB	73741815	73930348	14q24.2	Protein numb homolog
NUP54	77035812	77069668	4q21.1	Nucleoporin p54
NUP62	50410082	50433020	19q13.33	Nuclear pore glycoprotein p62
NUSAP1	41624892	41673248	15q15.1	Nucleolar and spindle-associated protein 1
NXF2	101470280	101581634	Xq22.1	Nuclear RNA export factor 2
OAS1	113344582	113369990	12q24.13	2'-5'-oligoadenylate synthase 1
OBRGRP	65886270	65901690	1p31.3	Leptin receptor gene-related protein
OCLN	68788119	68853931	5q13.2	Occludin
OGG1	9791628	9829903	3p25.3	N-glycosylase/DNA lyase
OGN	95146249	95166978	9q22.31	Mimecan
OLFML3	114522063	114524876	1p13.2	Olfactomedin-like protein 3
OLIG1	34442450	34444726	21q22.11	Oligodendrocyte transcription factor 1
OLR1	10310902	10324737	12p13.2	Oxidized low-density lipoprotein receptor 1
OPRL1	62711526	62731996	20q13.33	Nociceptin receptor
OPTN	13141449	13180291	10p13	Optineurin
OR2C3	247693434	247697141	1q44	Olfactory receptor 2C3
OR51E2	4701401	4719084	11p15.4	Olfactory receptor 51E2
ORC2L	201773696	201828403	2q33.1	Origin recognition complex subunit 2
ORC5L	103766788	103848495	7q22.1	Origin recognition complex subunit 5
OS9	58087738	58115340	12q13.3	Protein OS-9
OSBPL2	60813580	60871268	20q13.33	Oxysterol-binding protein-related protein 2
OSCAR	54597933	54606000	19q13.42	Osteoclast-associated immunoglobulin-like receptor
OSGEP	20914570	20923264	14q11.2	Probable tRNA N6-adenosine threonylcarbamoyltransferase
OSGEPL1	190611386	190627953	2q32.2	Probable tRNA N6-adenosine threonylcarbamoyltransferase, mitochondrial
OSM	30658818	30662829	22q12.2	Oncostatin-M
OTX1	63277192	63284971	2p15	Homeobox protein OTX1
OXCT	41730167	41870621	5p13.1	Succinyl-CoA:3-ketoacid coenzyme A transferase 1, mitochondrial

OXCT1	41730167	41870621	5p13.1	Succinyl-CoA:3-ketoacid coenzyme A transferase 1, mitochondrial
OXSR1	38206580	38296979	3p22.2	Serine/threonine-protein kinase OSR1
p21	112084840	112259313	1p13.2	Ras-related protein Rap-1A
P2RX7	121570622	121623876	12q24.31	P2X purinoceptor 7
P2RY12	151055168	151102600	3q25.1	P2Y purinoceptor 12
P2RY2	72929343	72947397	11q13.4	P2Y purinoceptor 2
P2RY6	72975550	73009662	11q13.4	P2Y purinoceptor 6
p53	7565097	7590856	17p13.1	Cellular tumor antigen p53
PA2G4	56498103	56507691	12q13.2	Proliferation-associated protein 2G4
PABPC1	101698044	101735037	8q22.3	Polyadenylate-binding protein 1
PACSIN1	34433916	34503006	6p21.31	Protein kinase C and casein kinase substrate in neurons protein 1
PACSIN3	47199076	47207994	11p11.2	Protein kinase C and casein kinase substrate in neurons protein 3
PAFAH2	26286258	26324648	1p36.11	Platelet-activating factor acetylhydrolase 2, cytoplasmic
PAGE1	49452053	49460596	Xp11.23	P antigen family member 1
PAGE4	49593863	49598576	Xp11.23	P antigen family member 4
PAICS	57301907	57327534	4q12	Multifunctional protein ADE2
PAK1IP1	10694928	10710015	6p24.2	p21-activated protein kinase-interacting protein 1
PAPOLB	4897364	4901625	7p22.1	Poly(A) polymerase beta
PAQR5	69591286	69700119	15q23	Membrane progestin receptor gamma
PARD6A	67694849	67696681	16q22.1	Partitioning defective 6 homolog alpha
PARL	183547173	183602721	3q27.1	Presenilins-associated rhomboid-like protein, mitochondrial
PARP1	226548392	226595780	1q42.12	Poly [ADP-ribose] polymerase 1
PAX6	31806340	31839509	11p13	Paired box protein Pax-6
PAX8	113973574	114036527	2q13	Paired box protein Pax-8
PCDHGC3	140855580	140892542	5q31.3	Protocadherin gamma-C3
PCK1	56136136	56141513	20q13.31	Phosphoenolpyruvate carboxykinase, cytosolic [GTP]
PCNA	5095599	5107272	20p12.3	Proliferating cell nuclear antigen
PCOLCE	100199800	100205798	7q22.1	Procollagen C-endopeptidase enhancer 1
PCSK2	17206752	17465223	20p12.1	Neuroendocrine convertase 2
PCSK5	78505560	78977255	9q21.13	Proprotein convertase subtilisin/kexin type 5
PDCD10	167401086	167452727	3q26.1	Programmed cell death protein 10
PDCD2	170884383	170893780	6q27	Programmed cell death protein 2
PDCD4	112631565	112659764	10q25.2	Programmed cell death protein 4
PDCD5	33071974	33078358	19q13.11	Programmed cell death protein 5
PDCD6	271736	353971	5p15.33	Programmed cell death protein 6
PDCL	125560668	125590910	9q33.2	Phosducin-like protein
PDE4A	10527449	10580305	19p13.2	cAMP-specific 3',5'-cyclic phosphodiesterase 4A
PDE6B	619373	664571	4p16.3	Rod cGMP-specific 3',5'-cyclic phosphodiesterase subunit beta
PDE6D	232597135	232650982	2q37.1	Retinal rod rhodopsin-sensitive cGMP 3',5'-cyclic phosphodiesterase subunit delta
PDEF	34505579	34524110	6p21.31	SAM pointed domain-containing Ets transcription factor
PDGFB	39619364	39640756	22q13.1	Platelet-derived growth factor subunit B
PDHB	58413357	58419584	3p14.3	Pyruvate dehydrogenase E1 component subunit beta, mitochondrial
PDIA3	44038590	44065477	15q15.3	Protein disulfide-isomerase A3
PDIA4	148700154	148725733	7q36.1	Protein disulfide-isomerase A4
PDIA6	10923517	10978103	2p25.1	Protein disulfide-isomerase A6

PDK4	95212811	95225803	7q21.3	[Pyruvate dehydrogenase (acetyl-transferring)] kinase isozyme 4, mitochondrial
PDPK1	2587965	2653189	16p13.3	3-phosphoinositide-dependent protein kinase 1
PEA15	160175127	160185166	1q23.2	Astrocytic phosphoprotein PEA-15
PENK	57349233	57359293	8q12.1	Proenkephalin-A
PEX10	2336236	2345236	1p36.32	Peroxisome biogenesis factor 10
PEX11A	90220995	90234014	15q26.1	Peroxisomal membrane protein 11A
PEX11B	145516252	145523730	1q21.1	Peroxisomal membrane protein 11B
PEX14	10532345	10690815	1p36.22	Peroxisomal membrane protein PEX14
PEX19	160246602	160256138	1q23.2	Peroxisomal biogenesis factor 19
PEX3	143771944	143811147	6q24.2	Peroxisomal biogenesis factor 3
PEX5	7341281	7371170	12p13.31	Peroxisomal targeting signal 1 receptor
PFKFB4	48555117	48599448	3p21.31	6-phosphofructo-2-kinase/fructose-2,6-bisphosphatase 4
PFKL	45719934	45747259	21q22.3	ATP-dependent 6-phosphofructokinase, liver type
PFKP	3108525	3179904	10p15.2	ATP-dependent 6-phosphofructokinase, platelet type
PFN1	4848947	4852356	17p13.2	Profilin-1
PGAM1	99185917	99193198	10q24.1	Phosphoglycerate mutase 1
PGCP	97657455	98161882	8q22.1	Carboxypeptidase Q
PGDS	139871956	139879887	9q34.3	Prostaglandin-H2 D-isomerase
PGLS	17622438	17632097	19p13.11	6-phosphogluconolactonase
PGRMC1	118370216	118378429	Xq24	Membrane-associated progesterone receptor component 1
PGS1	76374721	76421195	17q25.3	CDP-diacylglycerol--glycerol-3-phosphate 3-phosphatidyltransferase, mitochondrial
PHB	47481414	47492246	17q21.33	Prohibitin
PHF10	170104001	170124151	6q27	PHD finger protein 10
PHF11	50069746	50103123	13q14.2	PHD finger protein 11
PHF17	129730779	129796379	4q28.2	Protein Jade-1
PHKA2	18910418	19002716	Xp22.13	Phosphorylase b kinase regulatory subunit alpha, liver isoform
PHYH	13319796	13344412	10p13	Phytanoyl-CoA dioxygenase, peroxisomal
PI14	167159577	167196792	3q26.1	Serpin I2
PI3	43803517	43805185	20q13.12	Elafin
PIAS4	4007644	4039384	19p13.3	E3 SUMO-protein ligase PIAS4
PIGF	46808076	46844258	2p21	Phosphatidylinositol-glycan biosynthesis class F protein
PIGQ	616995	634136	16p13.3	Phosphatidylinositol N-acetylglucosaminyltransferase subunit Q
PIK3R3	46505812	46642160	1p34.1	Phosphatidylinositol 3-kinase regulatory subunit gamma
PIP	142829170	142836839	7q34	Prolactin-inducible protein
PIP5K2C	57984957	57997198	12q13.3	Phosphatidylinositol 5-phosphate 4-kinase type-2 gamma
PIPOX	27277531	27384234	17q11.2	Peroxisomal sarcosine oxidase
PIR	15402921	15511687	Xp22.2	Pirin
PITX2	111538579	111563279	4q25	Pituitary homeobox 2
PITX3	103989943	104001231	10q24.32	Pituitary homeobox 3
PIWIL4	94277006	94354587	11q21	Piwi-like protein 4
PKD2L1	102047903	102090243	10q24.31	Polycystic kidney disease 2-like 1 protein
PKIA	79428374	79517502	8q21.12	cAMP-dependent protein kinase inhibitor alpha
PKNOX1	44394620	44453691	21q22.3	Homeobox protein PKNOX1
PLA2G12A	110631145	110651233	4q25	Group XIIA secretory phospholipase A2
PLA2G2A	20301925	20306932	1p36.13	Phospholipase A2, membrane associated

PLA2G3	31530795	31536593	22q12.2	Group 3 secretory phospholipase A2
PLA2G4D	42359207	42386752	15q15.1	Cytosolic phospholipase A2 delta
PLAC1	133699868	133898352	Xq26.3	Placenta-specific protein 1
PLAGL1	144261437	144385735	6q24.2	Zinc finger protein PLAGL1
PLAGL2	30780306	30795594	20q11.21	Zinc finger protein PLAGL2
PLAT	42032236	42065242	8p11.21	Tissue-type plasminogen activator
PLAUR	44150247	44174699	19q13.31	Urokinase plasminogen activator surface receptor
PLCD4	219472488	219501907	2q35	1-phosphatidylinositol 4,5-bisphosphate phosphodiesterase delta-4
PLCG2	81772702	81991899	16q23.3	1-phosphatidylinositol 4,5-bisphosphate phosphodiesterase gamma-2
PLEK	68592305	68624585	2p13.3	Pleckstrin
PLEK2	67853700	67878917	14q23.3	Pleckstrin-2
PLEKHA1	124134212	124191867	10q26.13	Pleckstrin homology domain-containing family A member 1
PLEKHA8	30067020	30170096	7p14.3	Pleckstrin homology domain-containing family A member 8
PLEKHB2	131862420	132111282	2q21.1	Pleckstrin homology domain-containing family B member 2
PLEKHF2	96146032	96168912	8q22.1	Pleckstrin homology domain-containing family F member 2
PLEKHO1	150121373	150136916	1q21.2	Pleckstrin homology domain-containing family O member 1
PLK2	57749809	57756087	5q11.2	Serine/threonine-protein kinase PLK2
PLN	118869461	118881893	6q22.31	Cardiac phospholamban
PLS3	114795501	114885181	Xq23	Plastin-3
PLSCR3	7293046	7307416	17p13.1	Phospholipid scramblase 3
PLTP	44527399	44540794	20q13.12	Phospholipid transfer protein
PMP2	82352561	82359758	8q21.13	Myelin P2 protein
PMP22	15133095	15168643	17p12	Peripheral myelin protein 22
PMVK	154897210	154909467	1q21.3	Phosphomevalonate kinase
PNPLA4	7866288	7895780	Xp22.31	Patatin-like phospholipase domain-containing protein 4
PODN	53527854	53551174	1p32.3	Podocan
POLA2	65029233	65073060	11q13.1	DNA polymerase alpha subunit B
POLB	42195972	42229326	8p11.21	DNA polymerase beta
POLD1	50887461	50921273	19q13.33	DNA polymerase delta catalytic subunit
POLD2	44154286	44163957	7p13	DNA polymerase delta subunit 2
POLR2C	57496299	57505922	16q21	DNA-directed RNA polymerase II subunit RPB3
POLR2I	36604612	36606248	19q13.12	DNA-directed RNA polymerase II subunit RPB9
POLR2J	102113565	102119354	7q22.1	DNA-directed RNA polymerase II subunit RPB11-a
POLR2K	101162812	101166230	8q22.2	DNA-directed RNA polymerases I, II, and III subunit RPABC4
POLR2L	837356	842545	11p15.5	DNA-directed RNA polymerases I, II, and III subunit RPABC5
POLR3K	96407	103628	16p13.3	DNA-directed RNA polymerase III subunit RPC10
POMC	25383722	25391772	2p23.3	Pro-opiomelanocortin
POP4	30094924	30108144	19q12	Ribonuclease P protein subunit p29
PORCN	48367350	48379202	Xp11.23	Protein-serine O-palmitoleoyltransferase porcupine
POT1	124462440	124570037	7q31.33	Protection of telomeres protein 1
PPAP2B	56960419	57110974	1p32.2	Lipid phosphate phosphohydrolase 3
PPARG	12328867	12475855	3p25.2	Peroxisome proliferator-activated receptor gamma
PPFIBP2	7534529	7678358	11p15.4	Liprin-beta-2
PPGB	44518783	44527459	20q13.12	Lysosomal protective protein
PPIA	44836279	44864163	7p13	Peptidyl-prolyl cis-trans isomerase A

PPIB	64448011	64455404	15q22.31	Peptidyl-prolyl cis-trans isomerase B
PPID	159630286	159644548	4q32.1	Peptidyl-prolyl cis-trans isomerase D
PPIH	43124096	43142429	1p34.2	Peptidyl-prolyl cis-trans isomerase H
PPIL1	36822603	36842800	6p21.2	Peptidyl-prolyl cis-trans isomerase-like 1
PPIL5	50065415	50081390	14q21.3	Leucine-rich repeat protein 1
PPM1A	60712470	60765805	14q23.1	Protein phosphatase 1A
PPME1	73882144	73965748	11q13.4	Protein phosphatase methylesterase 1
PPP1CB	28974506	29025806	2p23.2	Serine/threonine-protein phosphatase PP1-beta catalytic subunit
PPP2R1A	52693292	52730687	19q13.41	Serine/threonine-protein phosphatase 2A 65 kDa regulatory subunit A alpha isoform
PPP2R2B	145967936	146464347	5q32	Serine/threonine-protein phosphatase 2A 55 kDa regulatory subunit B beta isoform
PPP4C	30087299	30096698	16p11.2	Serine/threonine-protein phosphatase 4 catalytic subunit
PRAME	22890123	22901768	22q11.22	Melanoma antigen preferentially expressed in tumors
PRCP	82534544	82681626	11q14.1	Lysosomal Pro-X carboxypeptidase
PRDM4	108126643	108155049	12q23.3	PR domain zinc finger protein 4
PRDX1	45976708	45988719	1p34.1	Peroxiredoxin-1
PRDX2	12907634	12912694	19p13.2	Peroxiredoxin-2
PRDX4	23682379	23704516	Xp22.11	Peroxiredoxin-4
PRELP	203444956	203460480	1q32.1	Prolargin
PRG1	43,349,056	43,349,548	19q13.2	P53-Responsive Gene 1
PRG2	57154267	57158130	11q12.1	Bone marrow proteoglycan
PRKAR1A	66507921	66547460	17q24.2	cAMP-dependent protein kinase type I-alpha regulatory subunit
PRKCA	64298754	64806861	17q24.2	Protein kinase C alpha type
PRKCDBP	6340176	6341877	11p15.4	Protein kinase C delta-binding protein
PRKR	102004319	102067123	7q22.1	PRKR-interacting protein 1
PRKRIP1	102004319	102067123	7q22.1	PRKR-interacting protein 1
PRL	22287480	22297730	6p22.3	Prolactin
PRM1	11374693	11375207	16p13.13	Sperm protamine P1
PRMT5	23389720	23398794	14q11.2	Protein arginine N-methyltransferase 5
PRNP	4666882	4682236	20p13	Major prion protein
Progranulin	42422614	42430470	17q21.31	Granulins
PROK1	110993822	110999976	1p13.3	Prokineticin-1
ProSAPiP1	3143263	3154192	20p13	Leucine zipper putative tumor suppressor 3
PRPF19	60658202	60674060	11q12.2	Pre-mRNA-processing factor 19
PRPS1	106871737	106894256	Xq22.3	Ribose-phosphate pyrophosphokinase 1
PRUNE	150980896	151008189	1q21.3	Protein prune homolog
PRV1	43857811	43867480	19q13.31	CD177 antigen
PSAP	73576055	73611126	10q22.1	Prosaposin
PSCD2	48972289	48985571	19q13.33	Cytohesin-2
PSG3	43225790	43244721	19q13.2	Pregnancy-specific beta-1-glycoprotein 3
PSG9	43715943	43773682	19q13.31	Pregnancy-specific beta-1-glycoprotein 9
PSMA1	14515329	14665181	11p15.2	Proteasome subunit alpha type-1
PSMA3	58711549	58738730	14q23.1	Proteasome subunit alpha type-3
PSMA4	78832747	78841604	15q25.1	Proteasome subunit alpha type-4
PSMA6	35747839	35786699	14q13.2	Proteasome subunit alpha type-6
PSMB4	151372010	151374420	1q21.3	Proteasome subunit beta type-4
PSMB6	4699439	4701790	17p13.2	Proteasome subunit beta type-6
PSMB8	32808494	32812480	6p21.32	Proteasome subunit beta type-8
PSMC3	47440320	47447993	11p11.2	26S protease regulatory subunit 6A
PSMD12	65334032	65362743	17q24.2	26S proteasome non-ATPase regulatory subunit 12

PSME1	24605367	24608176	14q12	Proteasome activator complex subunit 1
PSME2	24612574	24616779	14q12	Proteasome activator complex subunit 2
PSME3	40976402	40995774	17q21.31	Proteasome activator complex subunit 3
PSPC1	20248896	20357142	13q12.11	Paraspeckle component 1
PSTPIP1	77285700	77329673	15q24.3	Proline-serine-threonine phosphatase-interacting protein 1
PTE1	44470360	44486045	20q13.12	Acyl-coenzyme A thioesterase 8
PTEN	89622870	89731687	10q23.31	Phosphatidylinositol 3,4,5-trisphosphate 3-phosphatase and dual-specificity protein phosphatase PTEN
PTGES2	130882972	130890741	9q34.11	Prostaglandin E synthase 2
PTGES3	57057127	57082159	12q13.3	Prostaglandin E synthase 3
PTGS1	125132824	125157982	9q33.2	Prostaglandin G/H synthase 1
PTK9	44187526	44200178	12q12	Twinfilin-1
PTK9L	52262626	52273276	3p21.2	Twinfilin-2
PTMA	232571605	232578251	2q37.1	Prothymosin alpha
PTMS	6874682	6880116	12p13.31	Parathymosin
PTPN1	49126891	49201299	20q13.13	Tyrosine-protein phosphatase non-receptor type 1
PTPN6	7055631	7070479	12p13.31	Tyrosine-protein phosphatase non-receptor type 6
PTPRA	2844830	3019722	20p13	Receptor-type tyrosine-protein phosphatase alpha
PTTG1	159848829	159855748	5q33.3	Securin
PTTG1IP	46269500	46293752	21q22.3	Pituitary tumor-transforming gene 1 protein-interacting protein
PUS1	132413745	132428406	12q24.33	tRNA pseudouridine synthase A, mitochondrial
PVRL4	161040785	161059389	1q23.3	Nectin-4
PWP1	108079509	108106944	12q23.3	Periodic tryptophan protein 1 homolog
PYGB	25228705	25278650	20p11.21	Glycogen phosphorylase, brain form
PYGO2	154929502	154936329	1q21.3	Pygopus homolog 2
QDPR	17461884	17513857	4p15.32	Dihydropteridine reductase
RAB11A	66018392	66184329	15q22.31	Ras-related protein Rab-11A
RAB11B	8454865	8469318	19p13.2	Ras-related protein Rab-11B
RAB13	153954127	153958834	1q21.3	Ras-related protein Rab-13
RAB13	153954127	153958834	1q21.3	Ras-related protein Rab-13
RAB27A	55495164	55611311	15q21.3	Ras-related protein Rab-27A
RAB27A	55495164	55611311	15q21.3	Ras-related protein Rab-27A
RAB2B	21927179	21945132	14q11.2	Ras-related protein Rab-2B
RAB31	9708162	9862548	18p11.22	Ras-related protein Rab-31
RAB35	120532899	120555306	12q24.23	Ras-related protein Rab-35
RAB3IL1	61664773	61687741	11q12.2	Guanine nucleotide exchange factor for Rab-3A
RAB4A	229406822	229441641	1q42.13	Ras-related protein Rab-4A
RAB5A	19988571	20026667	3p24.3	Ras-related protein Rab-5A
RAB5B	56367697	56388490	12q13.2	Ras-related protein Rab-5B
RAB7L1	205737114	205744588	1q32.1	Ras-related protein Rab-7L1
RAB8A	16222439	16245044	19p13.12	Ras-related protein Rab-8A
RAB9A	13707244	13728625	Xp22.2	Ras-related protein Rab-9A
RABAC1	42460833	42463542	19q13.2	Prenylated Rab acceptor protein 1
RABL2B	51205929	51222091	22q13.33	Rab-like protein 2B
RACGAP1	50370706	50426919	12q13.12	Rac GTPase-activating protein 1
RAD1	34905369	34919094	5p13.2	Cell cycle checkpoint protein RAD1
RAD17	68665120	68710628	5q13.2	Cell cycle checkpoint protein RAD17
RAD18	8817088	9005457	3p25.3	E3 ubiquitin-protein ligase RAD18
RAD23B	110045418	110094475	9q31.2	UV excision repair protein RAD23 homolog B
RAD51	40986972	41024354	15q15.1	DNA repair protein RAD51 homolog 1
RAD51L1	68286496	69196935	14q24.1	DNA repair protein RAD51 homolog 2
RAD51L3	33426811	33448541	17q12	DNA repair protein RAD51 homolog 4
RAG2	36597124	36619829	11p12	V(D)J recombination-activating protein 2

RAMP1	238767536	238820756	2q37.3	Receptor activity-modifying protein 1
RAMP1	238767536	238820756	2q37.3	Receptor activity-modifying protein 1
RAPGEF3	48128455	48164823	12q13.11	Rap guanine nucleotide exchange factor 3
RARB	25215823	25639423	3p24.2	Retinoic acid receptor beta
RARRES2	150035408	150038763	7q36.1	Retinoic acid receptor responder protein 2
RARRES3	63304281	63313934	11q12.3	Retinoic acid receptor responder protein 3
RASD2	35936915	35950048	22q12.3	GTP-binding protein Rhes
RASSF5	206680879	206762616	1q32.1	Ras association domain-containing protein 5
RB1	48877887	49056122	13q14.2	Retinoblastoma-associated protein
RBBP4	33116743	33151812	1p35.1	Histone-binding protein RBBP4
RBBP5	205055270	205091143	1q32.1	Retinoblastoma-binding protein 5
RBM38	55966463	55984389	20q13.31	RNA-binding protein 38
RBM8A	145507598	145513536	1q21.1	RNA-binding protein 8A
RBMX	135930163	135962923	Xq26.3	RNA-binding motif protein, X chromosome
RBMY1F	24314689	24329129	Yq11.223	RNA-binding motif protein, Y chromosome, family 1 member F/J
RBP1	139236276	139258671	3q23	Retinol-binding protein 1
RBP4	95351444	95361501	10q23.33	Retinol-binding protein 4
RBP5	7276280	7281538	12p13.31	Retinol-binding protein 5
RCC1	28832455	28865812	1p35.3	Regulator of chromosome condensation
RCV1	9799637	9808938	17p13.1	Recoverin
REG4	120336641	120354283	1p12	Regenerating islet-derived protein 4
RENBP	153200716	153210232	Xq28	N-acylglucosamine 2-epimerase
REXO4	136271186	136283164	9q34.2	RNA exonuclease 4
RFC3	34392186	34540695	13q13.2	Replication factor C subunit 3
RFC4	186507669	186524847	3q27.3	Replication factor C subunit 4
RFP2	50570024	50594617	13q14.2	E3 ubiquitin-protein ligase TRIM13
RFXAP	37393361	37403241	13q13.3	Regulatory factor X-associated protein
RGS18	192127587	192154945	1q31.2	Regulator of G-protein signaling 18
RGS19	62704534	62711323	20q13.33	Regulator of G-protein signaling 19
RGS2	192778169	192781403	1q31.2	Regulator of G-protein signaling 2
RHAG	49572871	49604552	6p12.3	Ammonium transporter Rh type A
RHOBTB1	62629196	62761198	10q21.2	Rho-related BTB domain-containing protein 1
RHOC	113243728	113250056	1p13.2	Rho-related GTP-binding protein RhoC
RHOD	66824289	66839484	11q13.2	Rho-related GTP-binding protein RhoD
RHOH	40192673	40248587	4p14	Rho-related GTP-binding protein RhoH
RILP	1549444	1553371	17p13.3	Rab-interacting lysosomal protein
RIN1	66097713	66104311	11q13.2	Ras and Rab interactor 1
RLN1	5334969	5339873	9p24.1	Prorelaxin H1
RNASEH1	3592383	3606206	2p25.3	Ribonuclease H1
RNF12	73805052	73834452	Xq13.2	E3 ubiquitin-protein ligase RLIM
RNF182	13924677	13980533	6p23	E3 ubiquitin-protein ligase RNF182
RNF183	116059373	116065656	9q32	RING finger protein 183
RNF26	119205237	119208023	11q23.3	RING finger protein 26
RNF40	30773066	30787628	16p11.2	E3 ubiquitin-protein ligase BRE1B
RNF6	26706253	26796791	13q12.13	E3 ubiquitin-protein ligase RNF6
RNF7	141457046	141466402	3q23	RING-box protein 2
RNP24	124069078	124083116	12q24.31	Transmembrane emp24 domain-containing protein 2
RNPEP	201951500	201975275	1q32.1	Aminopeptidase B
RNPS1	2303117	2318413	16p13.3	RNA-binding protein with serine-rich domain 1
RNU3IP2	51967446	51975957	3p21.2	U3 small nucleolar RNA-interacting protein 2
RP11-444E17.2	44246480	44265458	6p21.1	T-complex-associated testis-expressed protein 1
RP13-36C9.1	134945598	134953994	Xq26.3	Cancer/testis antigen family 45 member A5
RPA2	28218035	28241257	1p35.3	Replication protein A 32 kDa subunit

RPL10	153618315	153637504	Xq28	60S ribosomal protein L10
RPL10A	35436185	35438562	6p21.31	60S ribosomal protein L10a
RPL11	24018269	24022915	1p36.11	60S ribosomal protein L11
RPL13	89627065	89630950	16q24.3	60S ribosomal protein L13
RPL14	40498783	40503861	3p22.1	60S ribosomal protein L14
RPL18	49118585	49122793	19q13.33	60S ribosomal protein L18
RPL23	37004118	37010096	17q12	60S ribosomal protein L23
RPL24	101399935	101405626	3q12.3	60S ribosomal protein L24
RPL26L1	172385732	172396774	5q35.1	60S ribosomal protein L26-like 1
RPL27	41150290	41154976	17q21.31	60S ribosomal protein L27
RPL28	55896713	55914612	19q13.42	60S ribosomal protein L28
RPL31	101618177	101640494	2q11.2	60S ribosomal protein L31
RPL32	12875984	12883087	3p25.2	60S ribosomal protein L32
RPL34	109541722	109551568	4q25	60S ribosomal protein L34
RPL35	127620159	127624260	9q33.3	60S ribosomal protein L35
RPL35A	197676858	197683481	3q29	60S ribosomal protein L35a
RPL36	5674958	5691887	19p13.3	60S ribosomal protein L36
RPL39	118920467	118925606	Xq24	60S ribosomal protein L39
RPL7A	136215069	136218281	9q34.2	60S ribosomal protein L7a
RPL9	39455744	39460568	4p14	60S ribosomal protein L9
RPLP0	120634489	120639038	12q24.23	60S acidic ribosomal protein P0
RPN2	35806813	35870022	20q11.23	Dolichyl-diphosphooligosaccharide--protein glycosyltransferase subunit 2
RPP30	92631473	92668312	10q23.31	Ribonuclease P protein subunit p30
RPS11	49999622	50002946	19q13.33	40S ribosomal protein S11
RPS13	17095936	17099334	11p15.1	40S ribosomal protein S13
RPS16	39923847	39926588	19q13.2	40S ribosomal protein S16
RPS17	82821158	82824972	15q25.2	40S ribosomal protein S17
RPS20	56979854	56987069	8q12.1	40S ribosomal protein S20
RPS21	60962172	60963576	20q13.33	40S ribosomal protein S21
RPS25	118886422	118889401	11q23.3	40S ribosomal protein S25
RPS26	56435637	56438116	12q13.2	40S ribosomal protein S26
RPS28	8386042	8388224	19p13.2	40S ribosomal protein S28
RPS3A	152020725	152025804	4q31.3	40S ribosomal protein S3a
RPS4Y1	2709527	2800041	Yp11.31	40S ribosomal protein S4, Y isoform 1
RPS5	58897767	58906173	19q13.43	40S ribosomal protein S5
RPS7	3622795	3628509	2p25.3	40S ribosomal protein S7
RPS9	54704610	54752862	19q13.42	40S ribosomal protein S9
RPSA	39448180	39454033	3p22.1	40S ribosomal protein SA
RRAD	66955582	66959547	16q22.1	GTP-binding protein RAD
RRAS	50138549	50143458	19q13.33	Ras-related protein R-Ras
RRM1	4115937	4160106	11p15.4	Ribonucleoside-diphosphate reductase large subunit
RSAD2	7005937	7038370	2p25.2	Radical S-adenosyl methionine domain-containing protein 2
RSPO3	127439749	127518910	6q22.33	R-spondin-3
RSU1	16632610	16859527	10p13	Ras suppressor protein 1
RTKN	74652963	74669549	2p13.1	Rhotekin
RUTBC3	40766595	40806293	22q13.1	Small G protein signaling modulator 3
RUVBL1	127783621	127872757	3q21.3	RuvB-like 1
RUVBL2	49496705	49519252	19q13.33	RuvB-like 2
RWDD1	116892530	116918838	6q22.1	RWD domain-containing protein 1
RWDD2	30376705	30391699	21q21.3	RWD domain-containing protein 2B
S100A11	152004982	152020383	1q21.3	Protein S100-A11
S100A16	153579362	153585621	1q21.3	Protein S100-A16

S100A3	153519805	153521848	1q21.3	Protein S100-A3
S100A4	153516089	153522612	1q21.3	Protein S100-A4
S100A6	153507075	153508720	1q21.3	Protein S100-A6
S100A7	153430220	153433177	1q21.3	Protein S100-A7
S100A9	153330330	153333503	1q21.3	Protein S100-A9
S100P	6694796	6698897	4p16.1	Protein S100-P
SAA1	18287721	18291524	11p15.1	Serum amyloid A-1 protein
SAA4	18252896	18258440	11p15.1	Serum amyloid A-4 protein
SAE1	47616531	47713886	19q13.32	SUMO-activating enzyme subunit 1
SART2	116575336	116762424	6q22.1	Dermatan-sulfate epimerase
SCAMP1	77656339	77776562	5q14.1	Secretory carrier-associated membrane protein 1
SCAMP3	155225770	155232221	1q22	Secretory carrier-associated membrane protein 3
SCAP2	26706681	27034858	7p15.2	Src kinase-associated phosphoprotein 2
SCARB1	125261402	125367214	12q24.31	Scavenger receptor class B member 1
SCGB1A1	62172575	62190667	11q12.3	Uteroglobin
SCGB1A1	62172575	62190667	11q12.3	Uteroglobin
SCHIP1	159557650	159615149	3q25.33	Schwannomin-interacting protein 1
SCIN	12610203	12693228	7p21.3	Adseverin
SCLY	238969530	239008054	2q37.3	Selenocysteine lyase
SCMH1	41492872	41707826	1p34.2	Polycomb protein SCMH1
SCNM1	151129140	151142773	1q21.3	Sodium channel modifier 1
SCO1	10583654	10601692	17p13.1	Protein SCO1 homolog, mitochondrial
SCYB6	73,836,497	73,849,064	4q13.3	Small Inducible Cytokine Subfamily B (Cys-X-Cys)
SCYD1	57406370	57418960	16q21	Fractalkine
SCYE1	106,315,544	106,349,226	4q24	Small Inducible Cytokine Subfamily E, Member 1 (Endothelial Monocyte-Activating)
SCYL3	169818772	169863408	1q24.2	Protein-associating with the carboxyl-terminal domain of ezrin
SDC1	20400558	20425194	2p24.1	Syndecan-1
SDC3	31342314	31381608	1p35.2	Syndecan-3
SDCCAG3	139296377	139305061	9q34.3	Serologically defined colon cancer antigen 3
SDF2	26975374	26989207	17q11.2	Stromal cell-derived factor 2
SDHD	111957497	111990353	11q23.1	Succinate dehydrogenase [ubiquinone] cytochrome b small subunit, mitochondrial
SDPR	192699028	192711981	2q32.3	Serum deprivation-response protein
SEC22C	42589461	42642572	3p22.1	Vesicle-trafficking protein SEC22c
SEC23B	18488137	18542059	20p11.23	Protein transport protein Sec23B
SEC24D	119643978	119759838	4q26	Protein transport protein Sec24D
SELL	169659808	169680839	1q24.2	L-selectin
SEMA3D	84624869	84816171	7q21.11	Semaphorin-3D
SEMA4F	74881355	74909186	2p13.1	Semaphorin-4F
SERF2	44069285	44094787	15q15.3	Small EDRK-rich factor 2
SERINC3	43124862	43150750	20q13.12	Serine incorporator 3
SERPINA5	95027779	95059457	14q32.13	Plasma serine protease inhibitor
SERPINA7	105277197	105282729	Xq22.3	Thyroxine-binding globulin
SERPINB4	61304493	61311532	18q21.33	Serpin B4
SERPIND1	21128167	21142008	22q11.21	Heparin cofactor 2
SERPINE1	100770370	100782547	7q22.1	Plasminogen activator inhibitor 1
SERPING1	57364860	57382326	11q12.1	Plasma protease C1 inhibitor
SERTAD1	40927499	40931932	19q13.2	SERTA domain-containing protein 1
SERTAD4	210406144	210419976	1q32.2	SERTA domain-containing protein 4
SESN2	28586038	28609002	1p35.3	Sestrin-2
SET	131445703	131458679	9q34.11	Protein SET
SF3A1	30727977	30752936	22q12.2	Splicing factor 3A subunit 1
SF3A2	2236520	2248678	19p13.3	Splicing factor 3A subunit 2

SFN	27189633	27190947	1p36.11	14-3-3 protein sigma
SFRS2	74730197	74733456	17q25.1	Serine/arginine-rich splicing factor 2
SFTPD	81697496	81742370	10q22.3	Pulmonary surfactant-associated protein D
SFXN2	104474295	104503249	10q24.32	Sideroflexin-2
SGCD	155297354	156194799	5q33.2	Delta-sarcoglycan
SGK	134490384	134639250	6q23.2	Serine/threonine-protein kinase Sgk1
SGNE1	32933877	32989299	15q13.3	Neuroendocrine protein 7B2
SGTA	2754712	2783369	19p13.3	Small glutamine-rich tetratricopeptide repeat-containing protein alpha
SH2D1A	123480194	123507005	Xq25	SH2 domain-containing protein 1A
SH2D1B	162365056	162381928	1q23.3	SH2 domain-containing protein 1B
SH2D3A	6752171	6767599	19p13.3	SH2 domain-containing protein 3A
SH3BGRL3	26605667	26608007	1p36.11	SH3 domain-binding glutamic acid-rich-like protein 3
SH3BP1	38030661	38062939	22q13.1	SH3 domain-binding protein 1
SH3GL1	4360367	4400544	19p13.3	Endophilin-A2
SH3GL2	17579080	17797127	9p22.2	Endophilin-A1
SH3GLB1	87170259	87213867	1p22.3	Endophilin-B1
SH3PX3	75940247	75954642	15q24.2	Sorting nexin-33
SIGIRR	405716	417455	11p15.5	Single Ig IL-1-related receptor
SIGLEC9	51628165	51639908	19q13.41	Sialic acid-binding Ig-like lectin 9
SIRT7	79869815	79879199	17q25.3	NAD-dependent protein deacetylase sirtuin-7
SKIP	228844666	229046361	2q36.3	A-kinase anchor protein SPHKAP
SKP1A	133484633	133512729	5q31.1	S-phase kinase-associated protein 1
SLC13A3	45186463	45304714	20q13.12	Solute carrier family 13 member 3
SLC16A7	59989848	60176395	12q14.1	Monocarboxylate transporter 2
SLC17A3	25833294	25882514	6p22.2	Sodium-dependent phosphate transport protein 4
SLC18A1	20002366	20040717	8p21.3	Chromaffin granule amine transporter
SLC19A3	228549926	228582728	2q36.3	Thiamine transporter 2
SLC1A4	65215611	65250999	2p14	Neutral amino acid transporter A
SLC1A7	53552855	53608289	1p32.3	Excitatory amino acid transporter 5
SLC20A2	42273993	42397069	8p11.21	Sodium-dependent phosphate transporter 2
SLC22A5	131705444	131731306	5q31.1	Solute carrier family 22 member 5
SLC22A8	62756626	62783311	11q12.3	Solute carrier family 22 member 8
SLC25A10	79670404	79688042	17q25.3	Mitochondrial dicarboxylate carrier
SLC25A15	41363548	41384247	13q14.11	Mitochondrial ornithine transporter 1
SLC25A18	18043139	18073760	22q11.21	Mitochondrial glutamate carrier 2
SLC25A19	73269073	73285591	17q25.1	Mitochondrial thiamine pyrophosphate carrier
SLC25A20	48894369	48936426	3p21.31	Mitochondrial carnitine/acylcarnitine carrier protein
SLC25A4	186064395	186071536	4q35.1	ADP/ATP translocase 1
SLC25A6	1505045	1511617	Xp22.33	ADP/ATP translocase 3
SLC27A3	153746830	153752633	1q21.3	Long-chain fatty acid transport protein 3
SLC2A3	8071826	8088871	12p13.31	Solute carrier family 2, facilitated glucose transporter member 3
SLC2A5	9095166	9148537	1p36.23	Solute carrier family 2, facilitated glucose transporter member 5
SLC2A6	136336217	136344259	9q34.2	Solute carrier family 2, facilitated glucose transporter member 6
SLC2A9	9772777	10056560	4p16.1	Solute carrier family 2, facilitated glucose transporter member 9
SLC31A1	115983808	116028674	9q32	High affinity copper uptake protein 1
SLC31A2	115913222	115926417	9q32	Probable low affinity copper uptake protein 2
SLC33A1	155538813	155572218	3q25.31	Acetyl-coenzyme A transporter 1
SLC35B1	47778305	47786376	17q21.33	Solute carrier family 35 member B1
SLC35C2	44978167	44993043	20q13.12	Solute carrier family 35 member C2

SLC38A2	46751972	46766650	12q13.11	Sodium-coupled neutral amino acid transporter 2
SLC38A5	48316920	48328644	Xp11.23	Sodium-coupled neutral amino acid transporter 5
SLC39A7	33168222	33172216	6p21.32	Zinc transporter SLC39A7
SLC3A1	44502599	44548633	2p21	Neutral and basic amino acid transport protein rBAT
SLC44A5	75667816	76076801	1p31.1	Choline transporter-like protein 5
SLC4A2	150754297	150773614	7q36.1	Anion exchange protein 2
SLC6A1	11034410	11080933	3p25.3	Sodium- and chloride-dependent GABA transporter 1
SLC6A13	329789	372039	12p13.33	Sodium- and chloride-dependent GABA transporter 2
SLC6A8	152953554	152962048	Xq28	Sodium- and chloride-dependent creatine transporter 1
SLC7A4	21383007	21387129	22q11.21	Cationic amino acid transporter 4
SLC7A9	33321415	33360672	19q13.11	b(0,+)-type amino acid transporter 1
SLCO3A1	92396925	92715665	15q26.1	Solute carrier organic anion transporter family member 3A1
SLCO4A1	61273797	61317137	20q13.33	Solute carrier organic anion transporter family member 4A1
SLPI	43880880	43883205	20q13.12	Antileukoproteinase
SMA3	58711549	58738730	14q23.1	Proteasome subunit alpha type-3
SMAD1	146402346	146479231	4q31.21	Mothers against decapentaplegic homolog 1
SMAD2	45357922	45457515	18q21.1	Mothers against decapentaplegic homolog 2
SMAF1	38,581,195	38,588,463	20q11.23	Small Adipocyte Factor 1
SMN1	70220768	70249769	5q13.2	Survival motor neuron protein
SMOC1	70320848	70499083	14q24.2	SPARC-related modular calcium-binding protein 1
SMTN	31460091	31500743	22q12.2	Smoothelin
SMU1	33041762	33076665	9p21.1	WD40 repeat-containing protein SMU1
SMYD3	245912642	246670614	1q44	Histone-lysine N-methyltransferase SMYD3
SNAI1	48599536	48605423	20q13.13	Zinc finger protein SNAI1
SNAPC1	62229075	62263146	14q23.2	snRNA-activating protein complex subunit 1
SNCB	176047085	176057530	5q35.2	Beta-synuclein
SNCG	88718375	88723017	10q23.2	Gamma-synuclein
SND1	127292234	127732661	7q32.1	Staphylococcal nuclease domain-containing protein 1
SNRPA1	101821715	101835487	15q26.3	U2 small nuclear ribonucleoprotein A'
SNRPD1	19192228	19210417	18q11.2	Small nuclear ribonucleoprotein Sm D1
SNRPD2	46190712	46195827	19q13.32	Small nuclear ribonucleoprotein Sm D2
SNTA1	31995761	32031698	20q11.21	Alpha-1-syntrophin
SNX10	26331541	26413949	7p15.2	Sorting nexin-10
SNX8	2291405	2393953	7p22.3	Sorting nexin-8
SNX9	158244296	158366109	6q25.3	Sorting nexin-9
SOAT1	179262925	179327815	1q25.2	Sterol O-acyltransferase 1
SOCS3	76352864	76356158	17q25.3	Suppressor of cytokine signaling 3
SOCS5	46926091	46990268	2p21	Suppressor of cytokine signaling 5
SORD	45315302	45369383	15q21.1	Sorbitol dehydrogenase
SORT1	109852192	109940573	1p13.3	Sortilin
SOX10	38366693	38383429	22q13.1	Transcription factor SOX-10
SOX5	23682440	24103966	12p12.1	Transcription factor SOX-5
SPARC	151040657	151066726	5q33.1	SPARC
SPARC	151040657	151066726	5q33.1	SPARC
SPATA22	3343313	3417146	17p13.2	Spermatogenesis-associated protein 22
SPATA6	48761044	48937845	1p33	Spermatogenesis-associated protein 6
SPHK2	49122548	49133974	19q13.33	Sphingosine kinase 2
SPINT1	41136216	41150405	15q15.1	Kunitz-type protease inhibitor 1

SPN	29674300	29682187	16p11.2	Leukosialin
SPOCK2	73818793	73848790	10q22.1	Testican-2
SPP1	88896819	88904562	4q22.1	Osteopontin
SPTLC2	77972340	78083116	14q24.3	Serine palmitoyltransferase 2
SQLE	126010739	126034525	8q24.13	Squalene monooxygenase
SQRDL	45923346	45983492	15q21.1	Sulfide:quinone oxidoreductase, mitochondrial
SRD5A1	6633456	6669675	5p15.31	3-oxo-5-alpha-steroid 4-dehydrogenase 1
SRPR	126132814	126139039	11q24.2	Signal recognition particle receptor subunit alpha
SS18	23596578	23671181	18q11.2	Protein SSXT
SSB	170648443	170668574	2q31.1	Lupus La protein
SSBP2	80708840	81047616	5q14.1	Single-stranded DNA-binding protein 2
SSR3	156257929	156272973	3q25.31	Translocon-associated protein subunit gamma
SSRP1	57093459	57103351	11q12.1	FACT complex subunit SSRP1
SSSCA1	65337901	65341413	11q13.1	Sjoegren syndrome/scleroderma autoantigen 1
SST	187386694	187388187	3q27.3	Somatostatin
SSTR2	71161151	71167185	17q25.1	Somatostatin receptor type 2
SSU72	1477053	1510249	1p36.33	RNA polymerase II subunit A C-terminal domain phosphatase SSU72
ST14	130029457	130080271	11q24.3	Suppressor of tumorigenicity 14 protein
ST3GAL1	134467091	134584183	8q24.22	CMP-N-acetylneuraminate-beta-galactosamide-alpha-2,3-sialyltransferase 1
ST5	8714898	8932498	11p15.4	Suppression of tumorigenicity 5 protein
ST7	116593292	116870157	7q31.2	Suppressor of tumorigenicity 7 protein
STAM2	152973315	153032506	2q23.3	Signal transducing adapter molecule 2
STAMBP	74056086	74100786	2p13.1	STAM-binding protein
STAR	38001167	38008783	8p11.23	Steroidogenic acute regulatory protein, mitochondrial
STAU2	74332604	74659943	8q21.11	Double-stranded RNA-binding protein Staufen homolog 2
STC2	172741716	172756506	5q35.1	Stanniocalcin-2
STIM1	3875757	4114439	11p15.4	Stromal interaction molecule 1
STK11	1189406	1228428	19p13.3	Serine/threonine-protein kinase STK11
STK17B	196998290	197041227	2q32.3	Serine/threonine-protein kinase 17B
STK24	99102455	99230194	13q32.2	Serine/threonine-protein kinase 24
STK25	242432089	242449145	2q37.3	Serine/threonine-protein kinase 25
STK38L	27396901	27478892	12p11.23	Serine/threonine-protein kinase 38-like
STMN2	80523049	80578410	8q21.13	Stathmin-2
STOM	124101355	124132531	9q33.2	Erythrocyte band 7 integral membrane protein
STOML2	35099888	35103154	9p13.3	Stomatin-like protein 2, mitochondrial
STUB1	730224	732870	16p13.3	E3 ubiquitin-protein ligase CHIP
STX6	180941861	180992047	1q25.3	Syntaxin-6
STYXL1	75625656	75677322	7q11.23	Serine/threonine/tyrosine-interacting-like protein 1
SUCNR1	151591431	151602405	3q25.1	Succinate receptor 1
SUFU	104263744	104393292	10q24.32	Suppressor of fused homolog
SULT1A1	28616903	28634946	16p11.2	Sulfotransferase 1A1
SULT2A1	48373723	48389654	19q13.33	Bile salt sulfotransferase
SUMO1	203070903	203103331	2q33.1	Small ubiquitin-related modifier 1
SUMO2	73163408	73179078	17q25.1	Small ubiquitin-related modifier 2
SUPT5H	39926796	39967310	19q13.2	Transcription elongation factor SPT5
SURF2	136223428	136228045	9q34.2	Surfeit locus protein 2
SURF4	136228325	136242970	9q34.2	Surfeit locus protein 4
SURVIVIN	76210267	76221717	17q25.3	Baculoviral IAP repeat-containing protein 5
SUV39H1	48553945	48567403	Xp11.23	Histone-lysine N-methyltransferase SUV39H1
SUV39H2	14920819	14946314	10p13	Histone-lysine N-methyltransferase SUV39H2
SYNGR3	2039661	2044276	16p13.3	Synaptogyrin-3

SYT12	66774249	66818334	11q13.2	Synaptotagmin-12
SYT3	51124564	51171651	19q13.33	Synaptotagmin-3
SYTL1	27668513	27680421	1p36.11	Synaptotagmin-like protein 1
TAC3	57403784	57422667	12q13.3	Tachykinin-3
TACSTD1	47572297	47614740	2p21	Epithelial cell adhesion molecule
TACSTD2	59041099	59043166	1p32.1	Tumor-associated calcium signal transducer 2
TAF11	34845555	34855866	6p21.31	Transcription initiation factor TFIID subunit 11
TAF1B	9983483	10074545	2p25.1	TATA box-binding protein-associated factor RNA polymerase I subunit B
TAF2I	34845555	34855866	6p21.31	Transcription initiation factor TFIID subunit 11
TAF6	99704693	99717464	7q22.1	Transcription initiation factor TFIID subunit 6
TAF9B	77385245	77395203	Xq21.1	Transcription initiation factor TFIID subunit 9B
TAGLN2	159887897	159895522	1q23.2	Transgelin-2
TAP1	32812986	32821755	6p21.32	Antigen peptide transporter 1
TARS	33440802	33469644	5p13.3	Threonine--tRNA ligase, cytoplasmic
TBL2	72983262	72993121	7q11.23	Transducin beta-like protein 2
TBX3	115108059	115121969	12q24.21	T-box transcription factor TBX3
TCEA1	54879112	54935089	8q11.23	Transcription elongation factor A protein 1
TCEAL1	102883632	102885881	Xq22.2	Transcription elongation factor A protein-like 1
TCEB3	24069645	24088549	1p36.11	Transcription elongation factor B polypeptide 3
TCF19	31126319	31134936	6p21.33	Transcription factor 19
TCL1A	96176304	96180533	14q32.13	T-cell leukemia/lymphoma protein 1A
TCN1	59620273	59634048	11q12.1	Transcobalamin-1
TD52	49411431	49493714	20q13.13	Breast carcinoma-amplified sequence 4
TEAD2	49843852	49865714	19q13.33	Transcriptional enhancer factor TEF-4
TEF	41763337	41795330	22q13.2	Thyrotroph embryonic factor
TEKT3	15207128	15244958	17p12	Tektin-3
TERF1	73921099	73960357	8q21.11	Telomeric repeat-binding factor 1
TESC	117476728	117537284	12q24.22	Calcineurin B homologous protein 3
TEX11	69748790	70128581	Xq13.1	Testis-expressed sequence 11 protein
TFAP2A	10393419	10419892	6p24.3	Transcription factor AP-2-alpha
TFAP2B	50786436	50815326	6p12.3	Transcription factor AP-2-beta
TFB1M	155578643	155635627	6q25.3	Dimethyladenosine transferase 1, mitochondrial
TFF1	43782391	43786703	21q22.3	Trefoil factor 1
TFF3	43731777	43735761	21q22.3	Trefoil factor 3
TFPI2	93514709	93520303	7q21.3	Tissue factor pathway inhibitor 2
TFRC	195754054	195809060	3q29	Transferrin receptor protein 1
TGFBR2	30647994	30735634	3p24.1	TGF-beta receptor type-2
TGIF	3411606	3458409	18p11.31	Homeobox protein TGIF1
TGIF2	35201891	35222353	20q11.23	Homeobox protein TGIF2
TGM4	44916100	44956482	3p21.31	Protein-glutamine gamma-glutamyltransferase 4
THAP11	67876213	67878097	16q22.1	THAP domain-containing protein 11
THRA	38214543	38250120	17q21.1	Thyroid hormone receptor alpha
THRAP5	867962	893218	19p13.3	Mediator of RNA polymerase II transcription subunit 16
THRAP6	118532952	118552501	8q24.11	Mediator of RNA polymerase II transcription subunit 30
TIMM13	2425622	2427892	19p13.3	Mitochondrial import inner membrane translocase subunit Tim13
TIMP1	47441712	47446188	Xp11.23	Metalloproteinase inhibitor 1
TIMP3	33197687	33259030	22q12.3	Metalloproteinase inhibitor 3
TIMP4	12194551	12200851	3p25.2	Metalloproteinase inhibitor 4
TINF2	24708849	24711880	14q12	TERF1-interacting nuclear factor 2
TITF1	36985602	36990354	14q13.3	Homeobox protein Nkx-2.1
TLE1	84198598	84304220	9q21.32	Transducin-like enhancer protein 1

TLR7	12885202	12908499	Xp22.2	Toll-like receptor 7
TLX3	170736288	170739138	5q35.1	T-cell leukemia homeobox protein 3
TM4SF1	149086809	149095652	3q25.1	Transmembrane 4 L6 family member 1
TMC6	76106539	76128488	17q25.3	Transmembrane channel-like protein 6
TMED1	10943114	10946994	19p13.2	Transmembrane emp24 domain-containing protein 1
TMED6	69377151	69385712	16q22.1	Transmembrane emp24 domain-containing protein 6
TMEM126B	85339629	85347580	11q14.1	Complex I assembly factor TMEM126B, mitochondrial
TMEM138	61129473	61136981	11q12.2	Transmembrane protein 138
TMEM14C	10723148	10731362	6p24.2	Transmembrane protein 14C
TMEM161B	87485450	87565293	5q14.3	Transmembrane protein 161B
TMEM31	102965837	102968956	Xq22.2	Transmembrane protein 31
TMEM66	29920528	29940723	8p12	Store-operated calcium entry-associated regulatory factor
TMEM8	55738007	55741647	19q13.42	Lysoplasmalogenase
TMOD1	100263462	100364030	9q22.33	Tropomodulin-1
TNA	45043040	45077563	3p21.31	Tetranectin
TNF	31543344	31546113	6p21.33	Tumor necrosis factor
TNFAIP1	26662628	26674035	17q11.2	BTB/POZ domain-containing adapter for CUL3-mediated RhoA degradation protein 2
TNFRSF10A	23047965	23082639	8p21.3	Tumor necrosis factor receptor superfamily member 10A
TNFRSF11B	119935796	119964439	8q24.12	Tumor necrosis factor receptor superfamily member 11B
TNFRSF12A	3068446	3072384	16p13.3	Tumor necrosis factor receptor superfamily member 12A
TNFRSF14	2487078	2496821	1p36.32	Tumor necrosis factor receptor superfamily member 14
TNFRSF19L	73087309	73108519	11q13.4	Tumor necrosis factor receptor superfamily member 19L
TNFRSF1A	6437923	6451280	12p13.31	Tumor necrosis factor receptor superfamily member 1A
TNFRSF9	7979907	8000926	1p36.23	Tumor necrosis factor receptor superfamily member 9
TNFSF13	7461609	7464925	17p13.1	Tumor necrosis factor ligand superfamily member 13
TNFSF14	6663148	6670599	19p13.3	Tumor necrosis factor ligand superfamily member 14
TNFSF6	172628154	172636014	1q24.3	Tumor necrosis factor ligand superfamily member 6
TNIP1	150409506	150473138	5q33.1	TNFAIP3-interacting protein 1
TNK1	7283853	7293093	17p13.1	Non-receptor tyrosine-protein kinase TNK1
TNNC1	52485118	52488086	3p21.1	Troponin C, slow skeletal and cardiac muscles
TNNC2	44451853	44462384	20q13.12	Troponin C, skeletal muscle
TNNI1	201373625	201398994	1q32.1	Troponin I, slow skeletal muscle
TNNI2	1860219	1862910	11p15.5	Troponin I, fast skeletal muscle
TNNI3	55663137	55669141	19q13.42	Troponin I, cardiac muscle
TOB1	48939584	48945339	17q21.33	Protein Tob1
TOB2	41829496	41843027	22q13.2	Protein Tob2
TOLLIP	1295601	1330884	11p15.5	Toll-interacting protein
TOMM22	39077953	39080818	22q13.1	Mitochondrial import receptor subunit TOM22 homolog
TOR1A	132575223	132586413	9q34.11	Torsin-1A
TP53	7565097	7590856	17p13.1	Cellular tumor antigen p53

TP53TG3	32684852	32688053	16p11.2	TP53-target gene 3 protein
TPD52	80870571	81143467	8q21.13	Tumor protein D52
TPD52L2	62496596	62522898	20q13.33	Tumor protein D54
TPI1	6976283	6980112	12p13.31	Triosephosphate isomerase
TPM1	63334831	63364114	15q22.2	Tropomyosin alpha-1 chain
TPSB1	1290697	1292555	16p13.3	Tryptase alpha/beta-1
TPTE	10906201	11029719	21p11.1	Putative tyrosine-protein phosphatase TPTE
TRA2A	23544399	23571660	7p15.3	Transformer-2 protein homolog alpha
TRADD	67188083	67194201	16q22.1	Tumor necrosis factor receptor type 1-associated DEATH domain protein
TRAF2	139776364	139821059	9q34.3	TNF receptor-associated factor 2
TRAF4	27071002	27077974	17q11.2	TNF receptor-associated factor 4
TRAF6	36508577	36531822	11p12	TNF receptor-associated factor 6
TRAFD1	112563305	112591407	12q24.13	TRAF-type zinc finger domain-containing protein 1
TRAM1	71485677	71520622	8q13.3	Translocating chain-associated membrane protein 1
TRAP1	3701640	3767598	16p13.3	Heat shock protein 75 kDa, mitochondrial
TRAPPC2	13730363	13752754	Xp22.2	Trafficking protein particle complex subunit 2
TREM1	41235664	41254457	6p21.1	Triggering receptor expressed on myeloid cells 1
TREX2	152710178	152736045	Xq28	Three prime repair exonuclease 2
TRIM22	5710919	5758319	11p15.4	E3 ubiquitin-protein ligase TRIM22
TRIM31	30070674	30080883	6p22.1	E3 ubiquitin-protein ligase TRIM31
TRIM32	119449581	119463579	9q33.1	E3 ubiquitin-protein ligase TRIM32
TRIM38	25963030	25987384	6p22.2	E3 ubiquitin-protein ligase TRIM38
TRIM7	180620924	180632293	5q35.3	Tripartite motif-containing protein 7
TRIM8	104404253	104418164	10q24.32	Probable E3 ubiquitin-protein ligase TRIM8
TRIP10	6737936	6751537	19p13.3	Cdc42-interacting protein 4
TROVE2	193028552	193060907	1q31.2	60 kDa SS-A/Ro ribonucleoprotein
TRPV2	16318856	16340317	17p11.2	Transient receptor potential cation channel subfamily V member 2
TSG101	18489883	18548779	11p15.1	Tumor susceptibility gene 101 protein
TSG101	18489883	18548779	11p15.1	Tumor susceptibility gene 101 protein
TSSC1	3192696	3381653	2p25.3	Protein TSSC1
TSSK6	19623227	19626838	19p13.11	Testis-specific serine/threonine-protein kinase 6
TTC2	41,976,421	42,021,376	17q21.2	Tetratricopeptide Repeat Protein 2
TTK	80713604	80752244	6q14.1	Dual specificity protein kinase TTK
TUBA6	49582519	49667114	12q13.12	Tubulin alpha-1C chain
TUBB2A	3153903	3157760	6p25.2	Tubulin beta-2A chain
TUBE1	112391980	112408732	6q21	Tubulin epsilon chain
TUBG1	40761694	40767252	17q21.2	Tubulin gamma-1 chain
TWIST1	19060614	19157295	7p21.1	Twist-related protein 1
TWIST2	239756673	239795893	2q37.3	Twist-related protein 2
TXN2	36863083	36878077	22q12.3	Thioredoxin, mitochondrial
TXN2	36863083	36878077	22q12.3	Thioredoxin, mitochondrial
TYK2	10461209	10491352	19p13.2	Non-receptor tyrosine-protein kinase TYK2
TYMS	657604	673578	18p11.32	Thymidylate synthase
TYROBP	36395303	36399197	19q13.12	TYRO protein tyrosine kinase-binding protein
U2AF1L4	36233365	36236346	19q13.12	Splicing factor U2AF 26 kDa subunit
UBADC1	138824815	138853226	9q34.3	Ubiquitin-associated domain-containing protein 1
UBAP1	34179003	34252521	9p13.3	Ubiquitin-associated protein 1
UBD	29523292	29527702	6p22.1	Ubiquitin D
UBE1C	69103881	69129559	3p14.1	NEDD8-activating enzyme E1 catalytic subunit
UBE2A	118708501	118718381	Xq24	Ubiquitin-conjugating enzyme E2 A
UBE2B	133706870	133727683	5q31.1	Ubiquitin-conjugating enzyme E2 B
UBE2B	133706870	133727683	5q31.1	Ubiquitin-conjugating enzyme E2 B
UBE2C	44441215	44445596	20q13.12	Ubiquitin-conjugating enzyme E2 C

UBE2I	1355548	1377019	16p13.3	SUMO-conjugating enzyme UBC9
UBE2J1	90036344	90062567	6q15	Ubiquitin-conjugating enzyme E2 J1
UBE2T	202300785	202311108	1q32.1	Ubiquitin-conjugating enzyme E2 T
UBE2V2	48920960	48977268	8q11.21	Ubiquitin-conjugating enzyme E2 variant 2
UBOX5	3088219	3140842	20p13	RING finger protein 37
UBXD2	135,741,619	135,785,063	2q21.3	UBX Domain-Containing Protein 2
UBXD8	175874629	175937075	5q35.2	FAS-associated factor 2
UCHL1	41258430	41270472	4p13	Ubiquitin carboxyl-terminal hydrolase isozyme L1
UCP2	73685712	73694352	11q13.4	Mitochondrial uncoupling protein 2
UFC1	161122566	161128646	1q23.3	Ubiquitin-fold modifier-conjugating enzyme 1
UGB	62172575	62190667	11q12.3	Uteroglobin
UGDH	39500375	39529931	4p14	UDP-glucose 6-dehydrogenase
UGT2B4	70345883	70391732	4q13.2	UDP-glucuronosyltransferase 2B4
UGT2B7	69917081	69978705	4q13.2	UDP-glucuronosyltransferase 2B7
UNRIP	16035325	16056412	12p12.3	Serine-threonine kinase receptor-associated protein
UQCRC2	21963981	21994981	16p12.2	Cytochrome b-c1 complex subunit 2, mitochondrial
UROD	45477819	45481247	1p34.1	Uroporphyrinogen decarboxylase
UROS	127477146	127511817	10q26.13	Uroporphyrinogen-III synthase
USP10	84733584	84813528	16q24.1	Ubiquitin carboxyl-terminal hydrolase 10
USP13	179370543	179507189	3q26.33	Ubiquitin carboxyl-terminal hydrolase 13
USP2	119225925	119252436	11q23.3	Ubiquitin carboxyl-terminal hydrolase 2
USP30	109460894	109525831	12q24.11	Ubiquitin carboxyl-terminal hydrolase 30
USP33	78161672	78225537	1p31.1	Ubiquitin carboxyl-terminal hydrolase 33
USP5	6961292	6975796	12p13.31	Ubiquitin carboxyl-terminal hydrolase 5
UTP14A	129040097	129063737	Xq26.1	U3 small nucleolar RNA-associated protein 14 homolog A
UXS1	106709759	106810795	2q12.2	UDP-glucuronic acid decarboxylase 1
VAMP1	6571403	6580153	12p13.31	Vesicle-associated membrane protein 1
VAMP5	85811531	85820535	2p11.2	Vesicle-associated membrane protein 5
VCL	75757872	75879918	10q22.2	Vinculin
VDAC1	133307606	133340824	5q31.1	Voltage-dependent anion-selective channel protein 1
VDRIP	48627459	48669267	13q14.2	Mediator of RNA polymerase II transcription subunit 4
VEGFB	64002010	64006259	11q13.1	Vascular endothelial growth factor B
VEGFC	177604689	177713881	4q34.3	Vascular endothelial growth factor C
VIL2	159186773	159240444	6q25.3	Ezrin
VILL	38029550	38048679	3p22.2	Villin-like protein
VPS18	41186628	41196173	15q15.1	Vacuolar protein sorting-associated protein 18 homolog
VSIG4	65241580	65259967	Xq12	V-set and immunoglobulin domain-containing protein 4
VTI2	68113792	68141548	14q24.1	Vesicle transport through interaction with t-SNAREs homolog 1B
VTN	26691290	26700110	17q11.2	Vitronectin
WBP11	14939410	14956474	12p12.3	WW domain-binding protein 11
WBSCR1	73588575	73611431	7q11.23	Eukaryotic translation initiation factor 4H
WBSCR27	73248920	73256865	7q11.23	Williams-Beuren syndrome chromosomal region 27 protein
WDR20	102605840	102691184	14q32.31	WD repeat-containing protein 20
WDR68	61627822	61671639	17q23.3	DDB1- and CUL4-associated factor 7
WNT11	75897369	75921780	11q13.5	Protein Wnt-11
WNT2	116916685	116963343	7q31.2	Protein Wnt-2
WNT5A	55499743	55523973	3p14.3	Protein Wnt-5a
WNT7A	13857755	13921618	3p25.1	Protein Wnt-7a

WNT9A	228106357	228135599	1q42.13	Protein Wnt-9a
WRNIP1	2765648	2787186	6p25.2	ATPase WRNIP1
WWOX	78133310	79246564	16q23.1	WW domain-containing oxidoreductase
WWTR1	149235022	149454501	3q25.1	WW domain-containing transcription regulator protein 1
XAB1	27851114	27874375	2p23.3	GPN-loop GTPase 1
XAB2	7684411	7694451	19p13.2	Pre-mRNA-splicing factor SYF1
XAGE2	52380351	52387001	Xp11.22	X antigen family member 2
XCL2	168510003	168513235	1q24.2	Cytokine SCM-1 beta
XLKD1	10578513	10633236	11p15.4	Lymphatic vessel endothelial hyaluronic acid receptor 1
XPA	100437191	100459639	9q22.33	DNA repair protein complementing XP-A cells
XRCC5	216972187	217071026	2q35	X-ray repair cross-complementing protein 5
YARS2	32880424	32908836	12p11.21	Tyrosine--tRNA ligase, mitochondrial
YBX1	43148098	43168020	1p34.2	Nuclease-sensitive element-binding protein 1
YKT6	44240567	44253893	7p13	Synaptobrevin homolog YKT6
YWHAG	75956116	75988348	7q11.23	14-3-3 protein gamma
YWHAZ	101928753	101965616	8q22.3	14-3-3 protein zeta/delta
ZCCHC17	31769842	31837783	1p35.2	Nucleolar protein of 40 kDa
ZDHHC4	6617065	6629005	7p22.1	Probable palmitoyltransferase ZDHHC4
ZDHHC5	57435219	57468659	11q12.1	Palmitoyltransferase ZDHHC5
ZDHHC7	85007787	85045141	16q24.1	Palmitoyltransferase ZDHHC7
ZFAND3	37787275	38122400	6p21.2	AN1-type zinc finger protein 3
ZFP36L2	43449541	43453748	2p21	Zinc finger protein 36, C3H1 type-like 2
ZMYND12	42896000	42921938	1p34.2	Zinc finger MYND domain-containing protein 12
ZMYND19	140476531	140484942	9q34.3	Zinc finger MYND domain-containing protein 19
ZNF350	52467596	52490109	19q13.41	Zinc finger protein 350
ZNF426	9638667	9649303	19p13.2	Zinc finger protein 426
ZNF461	37128094	37157755	19q13.12	Zinc finger protein 461
ZNF496	247460714	247495148	1q44	Zinc finger protein 496
ZNF559	9434448	9461838	19p13.2	Zinc finger protein 559
ZNHIT1	100860949	100867471	7q22.1	Zinc finger HIT domain-containing protein 1

Table S2. Positive aAb for EBNA spots (n=14) defined taking into account the normalization values (> mean of the normalized values of pCite spots + 3SD).

Samples	Diagnostic	Mean of normalized intensity data of EBNA spots	Nº of EBNA spots > mean pCite+3SD
51	Healthy donor	1.33	4/14
52	Healthy donor	1.02	0/14
53	Healthy donor	0.73	2/14
54	Healthy donor	0.66	2/14
55	Healthy donor	0.70	0/14
56	Healthy donor	0.88	0/14
57	Healthy donor	1.16	1/14
1	Metastatic sCRC	1.06	1/14
2	Metastatic sCRC	3.74	5/14
3	Metastatic sCRC	0.80	2/14
4	Metastatic sCRC	3.42	1/14
7	Metastatic sCRC	0.49	4/14
13	Metastatic sCRC	0.65	0/14
15	Metastatic sCRC	0.57	1/14
16	Metastatic sCRC	0.36	0/14
20	Metastatic sCRC	1.77	0/14
25	Metastatic sCRC	0.57	0/14
35	Metastatic sCRC	1.20	0/14
45	Metastatic sCRC	0.73	1/14

5	Non-metastatic sCRC	2.30	1/14
6	Non-metastatic sCRC	3.46	1/14
8	Non-metastatic sCRC	1.25	2/14
9	Non-metastatic sCRC	1.85	2/14
10	Non-metastatic sCRC	0.15	0/14
11	Non-metastatic sCRC	0.25	0/14
12	Non-metastatic sCRC	0.88	3/14
14	Non-metastatic sCRC	0.15	0/14
17	Non-metastatic sCRC	0.22	4/14
18	Non-metastatic sCRC	0.15	1/14
19	Non-metastatic sCRC	1.36	0/14
21	Non-metastatic sCRC	2.70	5/14
22	Non-metastatic sCRC	0.73	4/14
23	Non-metastatic sCRC	1.18	0/14
24	Non-metastatic sCRC	1.50	4/14
26	Non-metastatic sCRC	1.28	2/14
27	Non-metastatic sCRC	1.72	0/14
28	Non-metastatic sCRC	0.53	2/14
29	Non-metastatic sCRC	2.29	0/14
30	Non-metastatic sCRC	1.12	0/14
31	Non-metastatic sCRC	1.24	3/14
32	Non-metastatic sCRC	1.44	0/14
33	Non-metastatic sCRC	1.57	2/14
34	Non-metastatic sCRC	1.74	0/14
36	Non-metastatic sCRC	1.71	0/14
37	Non-metastatic sCRC	0.50	3/14
38	Non-metastatic sCRC	1.17	0/14
39	Non-metastatic sCRC	1.42	1/14
40	Non-metastatic sCRC	1.32	1/14
41	Non-metastatic sCRC	1.25	0/14
42	Non-metastatic sCRC	0.32	0/14

43	Non-metastatic sCRC	1.88	1/14
44	Non-metastatic sCRC	1.64	0/14
46	Non-metastatic sCRC	0.93	0/14
47	Non-metastatic sCRC	1.09	0/14
48	Non-metastatic sCRC	0.43	0/14
49	Non-metastatic sCRC	1.44	0/14
50	Non-metastatic sCRC	0.44	0/14

Table S3. aAbs profile against TAAs that exhibited implication to discriminate healthy donors ($n = 7$) vs. sCRC patients ($n = 50$). None of these TAAs have been identified as positive aAb in healthy donors.

TAA ID	*p*-value (U-Mann-Withney)	Nº sCRC (*n* = 50) with aAb positive	Fold change (FC)	
			Median FC sCRC	% sCRC > FC1
TEX11	0.0005	8/50	2.6	90
GORASP2	0.0007	2/50	3.1	82
C9orf80	0.0007	3/50	3.9	92
HMOX2	0.0008	13/50	3.0	92
KIF9	0.0008	14/50	2.7	92
ICAM2	0.0012	16/50	2.4	94
MARCKSL1	0.0012	10/50	2.4	88
RB1	0.0012	2/50	2.6	92
SPP1	0.0012	3/50	2.8	86
STC2	0.0013	10/50	2.0	84
BHMT2	0.0014	10/50	2.2	86
D21S2056E	0.0016	10/50	2.2	84
DLAT	0.0016	2/50	2.3	86
CKMT1B	0.0017	27/50	2.6	86
GTF2H1	0.0017	8/50	2.3	82
ALDOA	0.0019	8/50	2.0	88
COX11	0.0020	14/50	2.4	88
RPL11	0.0020	2/50	2.4	86
ASB3	0.0020	8/50	2.3	84
PRCP	0.0024	21/50	1.9	86
PDEF	0.0024	10/50	2.2	86
ANP32A	0.0024	2/50	2.3	82
GNAI3	0.0026	5/50	2.5	86
HBG1	0.0026	2/50	3.5	84
ARHI	0.0028	11/50	1.9	86
RCV1	0.0028	3/50	2.9	86
RAB8A	0.0028	5/50	2.4	84
GDEP	0.0030	11/50	2.2	82
PLAC1	0.0030	7/50	2.0	84
HSD17B3	0.0030	2/50	2.3	80
SH3BP1	0.0033	3/50	2.0	84
USP5	0.0033	2/50	2.1	82
TCEAL1	0.0033	5/50	2.5	84
KPNA6	0.0035	18/50	2.3	84
Progranulin	0.0038	4/50	1.8	86
CHODL	0.0038	4/50	2.2	86
KCNE2	0.0041	13/50	2.0	82
SERPINA5	0.0041	5/50	2.2	80
SLCO4A1	0.0041	4/50	2.5	80
SDPR	0.0041	10/50	1.8	86
JUP	0.0041	3/50	2.5	82
FRK	0.0041	6/50	2.1	84
DDX39	0.0044	2/50	2.1	80

PSAP	0.0048	2/50	2.1	84
SCARB1	0.0048	2/50	2.6	84
AMY2A	0.0052	9/50	2.1	84
BECN1	0.0056	16/50	2.0	84
ST14	0.0056	16/50	1.7	80
LDHB	0.0056	8/50	2.3	84
SNX10	0.0056	2/50	2.1	82
PSTPIP1	0.0060	17/50	2.1	84
SLC6A1	0.0065	6/50	2.0	78
HM13	0.0070	10/50	1.7	84
RHOH	0.0070	7/50	1.9	82
KIF22	0.0075	5/50	2.4	78
SYTL1	0.0075	4/50	2.1	80
Mage3	0.0075	10/50	1.9	82
HIST1H3D	0.0075	4/50	2.3	82
RWDD1	0.0080	9/50	2.1	80
RILP	0.0080	6/50	2.0	76
RPL35	0.0080	4/50	1.9	82
PLAGL1	0.0086	21/50	1.6	82
DDX56	0.0086	9/50	1.7	84
CTNNA1	0.0086	9/50	2.2	82
BET1	0.0086	4/50	2.0	78
HLA-DOB	0.0093	28/50	1.6	80
HSPC047	0.0093	4/50	1.9	80
PLEKHA8	0.0100	14/50	2.4	78
ANXA9	0.0100	2/50	1.7	80
GMDS	0.0100	7/50	1.7	84
CNP	0.0100	13/50	1.8	82
HSPA2	0.0100	2/50	2.2	76
FSCN1	0.0100	4/50	2.2	80
SERPING1	0.0107	5/50	2.2	80
TNIP1	0.0107	22/50	1.7	84
BCL2L13	0.0107	10/50	1.6	82
COPS5	0.0115	2/50	1.9	76
SKIP	0.0115	4/50	2.1	82
MAGEA9	0.0123	6/50	1.8	84
MGC26597	0.0131	16/50	1.8	80
OR2C3	0.0131	16/50	2.3	78
LOXL2	0.0131	6/50	2.1	80
SNCB	0.0131	7/50	1.8	78
SLC44A5	0.0131	4/50	1.8	80
RPS28	0.0131	4/50	2.2	80
PKNOX1	0.0141	13/50	1.6	80
PITX3	0.0141	2/50	1.8	80
C5AR1	0.0141	8/50	2.0	78
FHIT	0.0150	17/50	1.7	74
SND1	0.0150	6/50	1.7	80
ECRG4	0.0150	3/50	1.8	78
TCL1A	0.0161	20/50	1.6	80
NUP54	0.0161	11/50	2.3	78
SLC6A8	0.0161	16/50	1.9	76
DRD2	0.0172	8/50	1.6	78
FKRP	0.0172	6/50	1.6	78
PEX10	0.0172	2/50	1.7	82
COPB1	0.0172	3/50	1.8	78
YBX1	0.0184	9/50	1.5	76
NAT5	0.0184	15/50	1.7	76
CBLB	0.0184	3/50	1.7	76
SGTA	0.0196	6/50	1.6	76
BBS2	0.0209	4/50	1.6	76
CPSF1	0.0223	28/50	1.8	76
UGDH	0.0223	22/50	1.9	74
BGN	0.0223	10/50	1.6	76

ARRB2	0.0238	8/50	1.5	82
RPL18	0.0253	12/50	1.6	74
RPS9	0.0253	11/50	1.7	76
IFNGR2	0.0269	16/50	1.7	78
ANXA13	0.0269	13/50	1.6	76
OLR1	0.0269	7/50	1.9	70
RSU1	0.0287	14/50	1.6	76
LYZ	0.0287	8/50	1.5	74
P2RY6	0.0305	6/50	1.6	76
SAE1	0.0324	17/50	1.5	76
EIF3S8	0.0324	16/50	1.7	74
PENK	0.0324	15/50	1.7	72
H3F3A	0.0324	4/50	1.5	76
EBAG9	0.0324	2/50	1.9	70
C21orf66	0.0344	13/50	1.6	74
TSSK6	0.0344	11/50	1.6	74
ALDH1A2	0.0344	13/50	1.6	76
GNL1	0.0344	5/50	1.5	74
TOLLIP	0.0344	3/50	1.7	74
CXADR	0.0365	23/50	1.8	78
LASP1	0.0388	5/50	1.7	74
SERF2	0.0388	2/50	1.7	70
RAB2B	0.0411	14/50	1.5	72
ML-IAP	0.0411	8/50	1.5	76
ASPSCR1	0.0411	9/50	1.6	72
TEF	0.0436	12/50	1.7	76
STUB1	0.0436	18/50	1.6	74
VIL2	0.0436	8/50	2.1	72
EFNB1	0.0436	6/50	1.6	74
HPD	0.0436	3/50	1.9	72
TPTE	0.0462	3/50	1.6	74
KCTD14	0.0462	10/50	1.7	70
TRPV2	0.0489	11/50	1.6	70
C1orf62	0.0489	2/50	1.4	66
EML1	0.0489	2/50	1.7	72

Table S4. aAbs profile against TAAs with statistical significance ($p < 0.01$) that might distinguish metastatic sCRC ($n = 12$) vs. Non-metastatic sCRC ($n = 38$). None of these TAAs have been identified as positive aAb in healthy donors.

TAA ID	*p*-value (U-Mann-Withney)	Nº metastatic sCRC ($n = 12$) with aAb positive	Nº No metastatic sCRC ($n = 38$) with aAb positive	Fold change (FC)	
				Median FC	% sCRC > FC1
RPS28	0.0193	0/12	11/38	0.7	42
C9orf80	0.0327	8/12	5/38	0.9	50
OLR1	0.0387	0/12	18/38	0.6	50
FSCN1	0.0409	0/12	11/38	0.8	42
DLAT	0.0432	0/12	5/38	0.9	50
NUP54	0.0481	8/12	26/38	0.8	33

Table S5. Results from leave-one-out validation when the lasso algorithm selects only one variable.

Validation round	Protein selected	True positive	True negative	False positive	False negative
1	VTI_2	1	20	8	0
2	VTI_2	0	18	10	1
3	VTI_2	1	20	8	0
4	VTI_2	1	16	12	0
5	VTI_2	1	19	9	0
6	GJB2_1	1	17	11	0
7	VTI_2	1	18	10	0
8	VTI_2	1	21	7	0
9	CD151_1	1	15	13	0
10	VTI_2	1	19	9	0
11	VTI_2	1	19	9	0
12	VTI_2	1	16	12	0

Table S6. Identified proteins as potential aAbs biomarker panels in sCRC that appear in <10% of agarose based immunoprecipitation registered experiments in CRAPome.

User Input	Mapped Gene Symbol	Num of Expt. (found/total)	Ave SC	Max SC
DLAT	DLAT	38/411	6.5	24
VTI1B	VTI1B	2/411	1	1
P53	TP53	52/411	5.4	31
NUP54	NUP54	16/411	2.1	7
FSCN1	FSCN1	42/411	2.9	25
RPS28	RPS28	182/411	2.5	16
INIP	INIP	1/411	1	1
OLR1	OLR1	6/411	1.3	3
